# Integrative genome modeling platform reveals essentiality of rare contact events in 3D genome organizations

**DOI:** 10.1038/s41592-022-01527-x

**Published:** 2022-07-11

**Authors:** Lorenzo Boninsegna, Asli Yildirim, Guido Polles, Yuxiang Zhan, Sofia A. Quinodoz, Elizabeth H. Finn, Mitchell Guttman, Xianghong Jasmine Zhou, Frank Alber

**Affiliations:** 1 Institute of Quantitative and Computational Biosciences (QCBio), University of California, Los Angeles, Los Angeles, CA USA; 2 Department of Microbiology, Immunology, and Molecular Genetics, University of California, Los Angeles, Los Angeles, CA USA; 3 Department of Quantitative and Computational Biology, University of Southern California, Los Angeles, CA USA; 4 Division of Biology and Biological Engineering, California Institute of Technology, Pasadena, CA USA; 5 National Cancer Institute, National Institutes of Health, Bethesda, MD USA; 6 Department of Pathology, David Geffen School of Medicine, University of California, Los Angeles, Los Angeles, CA USA

**Keywords:** Computational models, Computational biophysics, Nuclear organization, Genomics, Statistical methods

## Abstract

A multitude of sequencing-based and microscopy technologies provide the means to unravel the relationship between the three-dimensional organization of genomes and key regulatory processes of genome function. Here, we develop a multimodal data integration approach to produce populations of single-cell genome structures that are highly predictive for nuclear locations of genes and nuclear bodies, local chromatin compaction and spatial segregation of functionally related chromatin. We demonstrate that multimodal data integration can compensate for systematic errors in some of the data and can greatly increase accuracy and coverage of genome structure models. We also show that alternative combinations of different orthogonal data sources can converge to models with similar predictive power. Moreover, our study reveals the key contributions of low-frequency (‘rare’) interchromosomal contacts to accurately predicting the global nuclear architecture, including the positioning of genes and chromosomes. Overall, our results highlight the benefits of multimodal data integration for genome structure analysis, available through the Integrative Genome Modeling software package.

## Abstract (web summary)

The Integrative Genome Modeling platform is a tool for population-based three-dimensional genome structure modeling and analysis by integrating various experimental data sources.

## Main

The spatial organization of eukaryotic genomes plays crucial roles in regulation of transcription, replication and cell differentiation, while malfunctions in chromatin structure is linked to disease, including cancer and premature aging disorders^1,2^. Advances in chromosome conformation capture (3C)-based^3–10^ and ligation-free methods^11–13^ and, most recently, live-cell and super-resolution microscopy^14–18^, have shed light onto key elements of genome structure organization, including the genome-wide detection of chromatin loops^19,20^, topologically associating domains (TADs)^21^ that modulate long-range promoter–enhancer interactions^12,22^ as well as the segregation of chromatin into nuclear compartments^8,10,23–26^. Each technology probes different aspects of genome architecture at different resolutions^1,27–29^.

These complementary methods provide a renewed opportunity to generate quantitative, highly predictive structural models of the entire nuclear organization^30^. Embedding data into three-dimensional (3D) structures is beneficial for a variety of reasons. First, all data itself originate from (often a large population of) 3D structures; so, reverse engineering that data and relating it back to an ensemble of representative 3D structures appears to be the natural way for integrating data from complementary methods via an appropriate representation of experimental errors and uncertainties. Second, generating structures consistent with multimodal data from heterogeneous and independent sources allows cross-validation of orthogonal data itself. Finally, 3D structures give access to features that are not immediately visible in the original input dataset, which can be compared with experimental data tailored to assess model predictivity. Yet, embedding data into 3D structures is a challenging task: not only is there no established protocol for data interpretation and modeling, but genome structures are dynamic in nature and can substantially vary between individual cells. A probabilistic description is thus needed surpassing traditional structural modeling that limits to a single equilibrium structure, or a small number of metastable structures.

There are several data-driven and mechanistic modeling strategies, which differ in the functional interpretation of data and sampling strategies, for generating an ensemble of 3D genome structures statistically consistent with it^23,25,26,31–50^. These 3D structures are then examined to derive structure–function correlations and make quantitative predictions about structural features of genomic regions, study their cell-to-cell variabilities and link these to functional observations. Most strategies have relied primarily on Hi-C data, which is abundant and straightforward to interpret in terms of chromatin contacts. However, data from a single experimental method cannot possibly capture all aspects of the spatial genome organization. Integrating data from a wide range of technologies, each with complementary strengths and limitations, will likely increase accuracy and coverage of genome structure models. Several methods were adapted to combine Hi-C with one other data source^14,37,39,49,51,52^; nevertheless, developing hybrid methods that can systematically integrate data from many different technologies to generate structural maps of entire diploid genomes remains a major challenge.

Here we present a population-based deconvolution method that provides a probabilistic framework for comprehensive and multimodal data integration. Our approach^30,36,44^ de-multiplexes ensemble data into a population of 3D structures, each governed by a unique pseudo-energy function, representing a subset of the data, hence explicitly factoring in the heterogeneity of structural features across different cells. The method produces highly predictive models of the folded states of complete diploid genomes, which are statistically consistent with all input data, and is therefore distinct from resampling methods^32,34,41,45,46^.

Our generalized framework generates fully diploid genome models from integration of four orthogonal data types: ensemble Hi-C^10^, lamin B1 DamID^24,53,54^, large-scale HIPMap 3D fluorescence in situ hybridization (FISH) imaging^55,56^ and data from single-cell split-pool recognition of interactions by tag extension (SPRITE) experiments^11^. Such models are capable of successfully predicting with good accuracy orthogonal experimental data from a variety of other genomics-based and super-resolution imaging experiments, such as data from SON TSA-seq experiments^57^ and DNA-MERFISH imaging^17^. Specifically, our structures predict with good accuracy gene distances to nuclear speckles, gene distances to the nuclear lamina and therefore allow an in-depth analysis of the nuclear microenvironment of genes at a genome-wide scale.

We further demonstrate that integration of all data modalities produces structures of maximal accuracy and show that different combinations of data types can lead to structures of comparable accuracy. For a given available data type, we can therefore propose which additional data types would maximize the prediction accuracy of the resulting structures. Also, our results highlight that relatively low-frequency interchromosomal contacts are essential to correctly predict whole-genome structure organizations: indeed, a modified Hi-C dataset with artificially underrepresented interchromosomal contacts severely fails at reproducing the correct global genome architecture. However, integrating additional data sources from other experiments can compensate for these biases and generate structure populations with still high predictivity accuracy. Our method is potentially applicable to other cell types and organisms, with different combinations of data as described here.

Our work represents the effort at integrating orthogonal data types from Hi-C, lamina DamID, 3D HIPMap FISH and DNA SPRITE experiments to produce highly predictive genome structure populations, which ultimately showcases the benefits of multimodal data integration in the context of whole-genome modeling. Due to its modular architecture, the method we propose can be easily adapted to incorporate other data types in the modeling pipeline, as we strive for even more realistic and predictive structures to dissect the genome structure–function relationship.

## Results

### Multimodal data-driven population modeling as an optimization problem

**Fig. 1:**
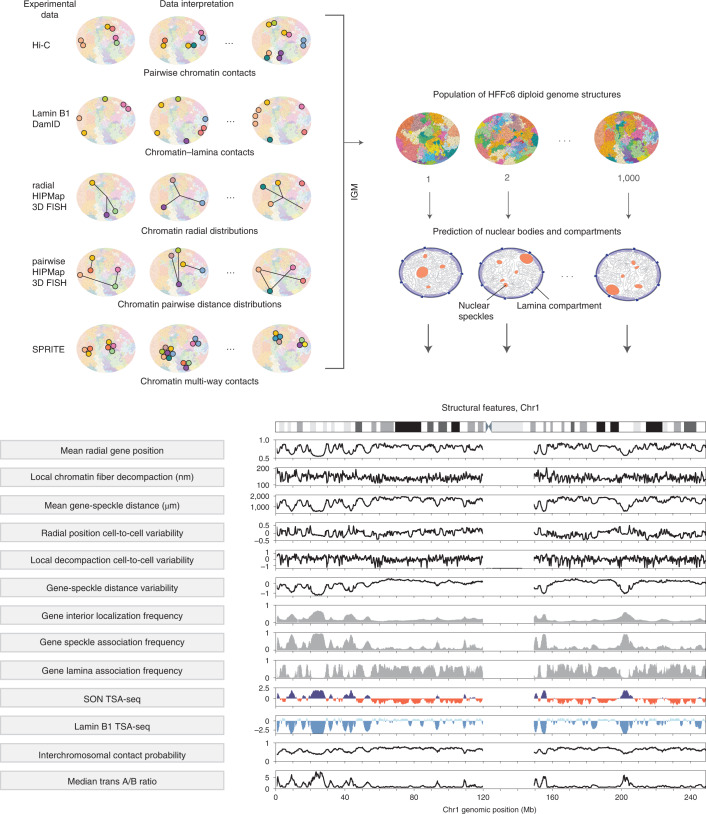
Prediction of the nuclear microenvironments of genes from genome structures. Top, schematic of the data-driven modeling approach. Information provided by orthogonal data modalities (Hi-C, lamina DamID, radial and pairwise HIPMap 3D FISH and DNA SPRITE) is used as input to the Integrative Genome Modeling (IGM) platform to generate a population of *S* = 1,000 diploid genome structures. Structures can be used to predict locations of nuclear bodies and compartments (nuclear speckles and lamina compartment), which can serve as reference points to describe locations of genes and the genome architecture. Bottom, the predicted genome structure population gives access to a large number of structural features (left), which collectively describe the nuclear microenvironment of genes on a genome-wide scale.

We expand our previous genome modeling framework^36,37,44^ and introduce a generalized formulation for the integration of a variety of orthogonal data to generate a population of full genome structures that simultaneously recapitulate all the data. Our method incorporates data types that relate to single genomic regions, such as lamin B1 DamID or radial 3D HIPMap FISH, to two genomic regions, such as Hi-C or pairwise 3D HIPMap FISH and several genomic regions, such as single-cell SPRITE experiments (Fig. 1). Our method incorporates both ensemble and single-cell data by deconvoluting ensemble data into a population of distinct single-cell genome structures, which cumulatively recapitulate all input information. Our model is defined as a population of *S* diploid genome structures $X=\left\{ {\boldsymbol{X}}_1, {\boldsymbol{X}}_2, \ldots, {\boldsymbol{X}}_S \right\}$, where each structure ***X***_*s*_ is represented by a set of 3D vectors representing the coordinates of all diploid chromatin regions. Given a collection of input data ${{{\mathcal{D}}}}_k$ from *K* different data sources, ${\frak{D}} = \left\{ {{{{\mathcal{D}}}}_k|k = 1, \ldots ,K} \right\}$, we aim to estimate the structure population ${{{\hat{\boldsymbol X}}}}$ such that the likelihood $P({\frak{D}}|{{{\boldsymbol{X}}}})$ is maximized. Because most experiments, such as Hi-C and lamina DamID, provide data that are averaged over a large population of cells, and often produce unphased data, they do not reveal which contacts coexist in which structure of the population or between which homologous chromosome copies. To represent this missing information at single-cell and diploid levels, we introduce data indicator tensors ${{{\mathcal{D}}}}_k^ \ast$ for each of the data sources ${\frak{D}}^ \ast = \left\{ {{{{\mathcal{D}}}}_k^ \ast |k = 1, \ldots ,K} \right\}$ as latent variables that augment all missing information in ${{{\mathcal{D}}}}_k$ (Methods and Supplementary Table 1). Thus, the latent variables ${\frak{D}}^ \ast$ are a detailed expansion of ${\frak{D}}$ at the diploid and single-structure representation. To determine a population of genome structures consistent with all experimental data, we therefore formulate a so-called hard expectation–maximization (EM) problem, where we jointly optimize all genome structure coordinates ***X*** and all latent variables.

$${\hat{\mathbf{X}}},{\hat{\frak{D}}} = argmax_{{\boldsymbol{X}},{\frak{D}}^*}\,log\,P({\frak{D}},{\frak{D}}^*\lceil{\boldsymbol{X}})$$

**Extended Data Fig. 1:**
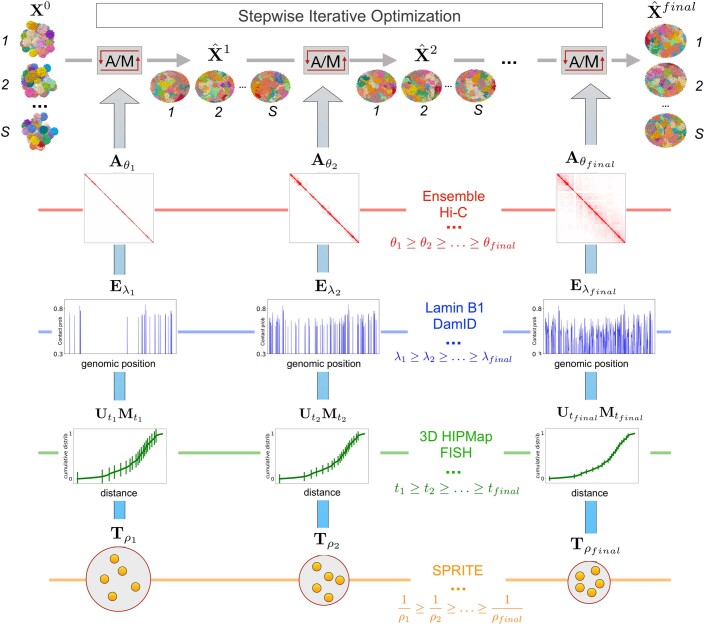
Flowchart of the Stepwise Iterative Optimization pipeline. Ensemble Hi-C, lamina DamID, 3D HIPMap FISH and SPRITE data are used as input to the Stepwise Iterative Optimization protocol which underlies the Integrated Genome Modeling platform. A randomly initialized diploid genome population with chromosome territories ***X***^0^ is first thermally relaxed subject to envelope and polymer restraints only (not shown). Then, genomic data are gradually added and structures are optimized via a sequence of iterative A/M optimization steps. Optimization hardness is gradually increased by adding batches of data and reducing the tolerance, as visually indicated (see also *Methods*). For example, at the end of i-th A/M step, all contacts with probability larger than ***θ***_***i***_ (that is, all matrix entries specified by ${{{\boldsymbol{A}}}}_{\theta _{{{i}}}}$), all lamina contacts with probability larger than $\lambda _{{{i}}}$ (that is, all entries ${{{\boldsymbol{E}}}}_{\lambda _{{{i}}}}$), all 3D HIPMap FISH distances with a tolerance equal to ${{{\boldsymbol{t}}}}_{{{i}}}$ (that is, ${{{\boldsymbol{U}}}}_{{{{t}}}_i}$and ${{{\boldsymbol{M}}}}_{{{{t}}}_i}$) and all SPRITE clusters with volume density ***ρ***_*i*_ (that is $\mathbf{T}_{\rho _i}$) are included (see *Methods*). Multiple sequential A/M iterations may be needed for a given set of optimization thresholds in order to generate an intermediate population ${{{\hat{\boldsymbol X}}}}^{({{{i}}})}$ which successfully incorporates all the data restraints that have been added up to that point. At the end of the pipeline, all data up to the final threshold values are included, and, after additional iterations lead to convergence (all data is satisfied), the optimized population ${{{\hat{\boldsymbol X}}}}^{({{{final}}})}$ is returned, together with the final violation statistics (see also Extended Data Fig. 2).

The solution of such a high-dimensional maximum likelihood problem requires extensive exploration of the space of all genome structure populations, which we achieve by using a series of optimization strategies for efficient and scalable model estimation (Methods, Supplementary Information and Extended Data Fig. 1)^36,37,44^. Convergence to an optimal solution $({{{\hat{\boldsymbol X}}}},\hat {\frak{D}}^ \ast )$ is reached when the models statistically reproduce all the input data (details of the mathematical formulation of data types, likelihood *P* and optimization strategy are provided in the Methods and Supplementary Information). The optimized structure population ***X̂*** is then used to determine locations of nuclear bodies in each single-cell model, which in turn serve as reference points to calculate a host of structural features. These features allow a thorough characterization of the nuclear microenvironment of each gene^30^ (Fig. 1).

### Comprehensive data-driven genome population structures of HFFc6 cell line

**Fig. 2:**
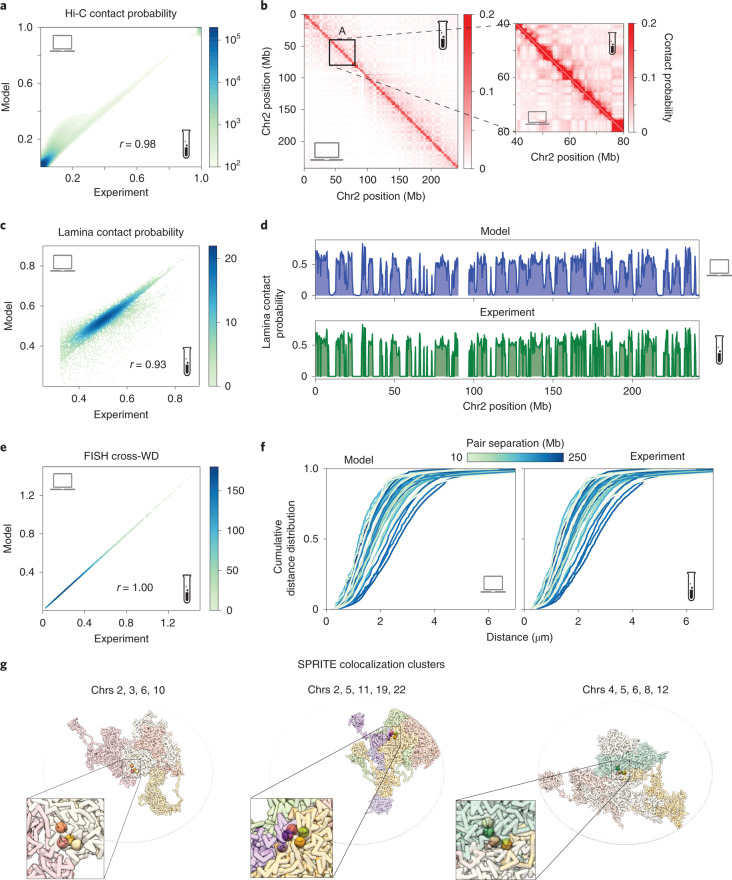
Input data are recapitulated in the genome structure population. **a**, Genome-wide correlation of Hi-C contact frequencies (interchromosomal and intra-chromosomal) between experiment^58^ and simulation (*r* = 0.98). **b**, Comparison between experimental (upper diagonal region) and simulated (lower diagonal region) contact frequency maps for chromosome 2 (left) and zoomed-in region (right). **c**,**d**, Correlation of lamin B1 DamID-derived contact probabilities between experiment^59^ and model genome wide (**c**) (*r* = 0.93) and visual comparison of both signals for chromosome 2 (**d**). **e**, Correlation of cross-Wasserstein distance (WD) between experimental FISH data and predictions (*r* = 1.00; Methods). **f**, Cumulative distributions of pairwise FISH distances for the set of 51 pairs of loci measured in 3D HIPMap FISH experiments^55^, plotted for both model (left) and experiment (right). Colors indicate the sequence separation in the chromosome between imaged loci pairs, with darker hues indicating larger sequence separations. **g**, Examples of single-cell SPRITE clusters from three different structures, showing colocalization of loci in a single-cell structure. Colors distinguish chromosomes, and homologs are shown in the same color. Loci in the same SPRITE cluster are also shown enlarged; left cluster: chr2: 150,927,500, chr3: 6,265,500, chr6: 93,928,500, chr10: 11,602,500; center cluster: chr2: 4,872,500, chr5: 23,208,500, chr11: 57,966,500, chr19: 51,314,500, chr22: 42,294,500; right cluster: chr4: 42,821,500, chr5: 68,438,500, chr6: 106,123,500, chr8: 85,891,500, chr12: 99,185,500. Clusters assayed experimentally^11^, including those shown, are reproduced in our structures.

**Extended Data Fig. 2:**
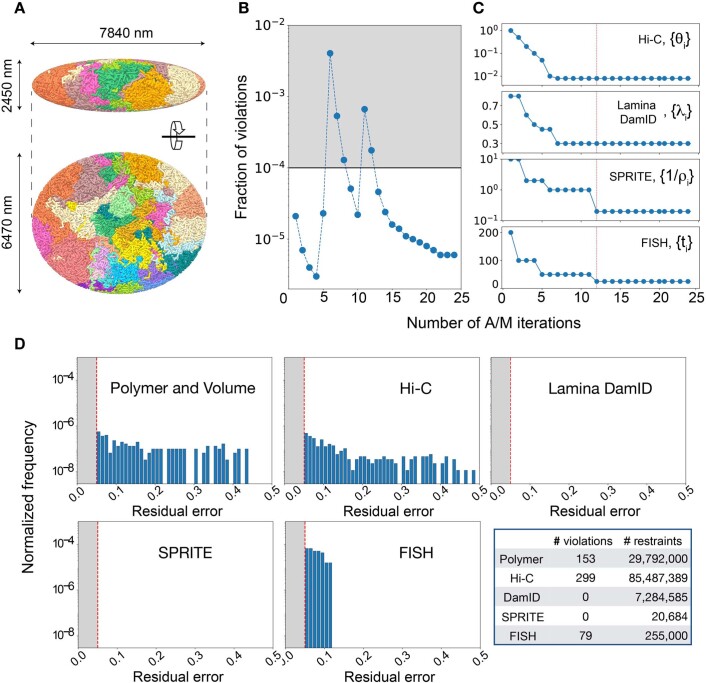
Optimization statistics for HFFc6 all-data genome model. (**A**) Top and side view of one full genome structure from the optimized HDSF population, with the ellipsoidal nuclear lamina axes annotated (in nm): the same color is used for homologous chromosomes. (**B**) Fraction of violations plotted as a function of A/M iterations during the HDSF population optimization: jumps in the curve (iterations 6 and 11) indicate the gradual addition of more data batches (that is data added at optimization thresholds (Methods)). All data are added by iteration 12, but additional iterations are run to ensure robust convergence with a violation fraction < 10^−5^. (**C**) Optimization thresholds (*θ*_*i*_,*λ*_*i*_,*t*_*i*_ and *ρ*_*i*_^−1^), which control the rate and size of data batches being added, shown as a function of the number of A/M iterations: a red vertical line indicates the iteration when all data points are added to the modeling. Final values are non-zero, which reproduces typical experimental setups where finite precision is only available. $\theta _{final} = \theta _{final}^{intra} = 0.008$ (Hi-C probability), *λ*_*final*_ = 0.3 (lamina DamID probability), *t*_*final*_ = 25*nm* (FISH distance tolerance), *ρ*_*final*_ = 0.005*nm*^−3^ (SPRITE volume density), see also *Methods* and Extended Data Fig. 1. (**D**) Final violation statistics broken down into the different restraint categories; each panel shows the normalized histogram of residual errors (*η* > 0.05, see *Supplementary Information*) associated with violations in a given data category. No bars are showing in the SPRITE panel because all applied SPRITE restraints are satisfied, and none is violated. The accompanying table details the number of applied restraints and the number of violations: over 99.999% of polymer restraints, over 99.999% of Hi-C restraints, 99.98% of FISH restraints, and 100% of both SPRITE and lamina DamID restraints are satisfied in the optimized population. The number of FISH and SPRITE restraints is orders of magnitude smaller than polymer, Hi-C and DamID restraints.

**Extended Data Fig. 3:**
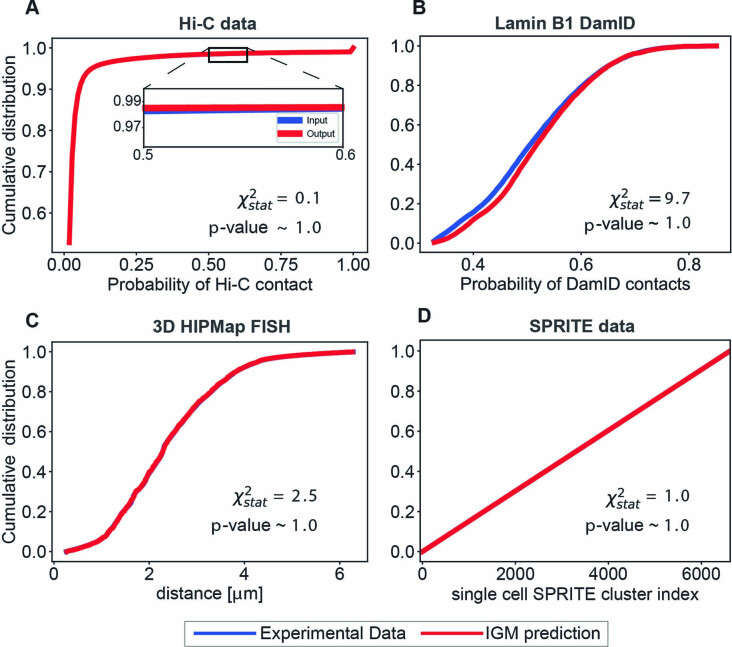
*χ*^2^ goodness-of-fit test between the predicted data from IGM HDSF populations and the input data from experiments. Each panel compares the cumulative probability distributions from experiments (blue) and simulation (red). For Hi-C (**A**) and laminB1 DamID data (**B**), the cumulative distributions of probability of contacts of a locus with another locus (Hi-C) or the nuclear envelope (DamID) are compared. (**C**) To demonstrate the good agreement between 3D HIPMap data from experiment and models, we show an example for a distribution of pairwise distances between loci 2.4 Mb and 273.5 Mb for chromosome 1. All the other distance distributions are also accurately reproduced with p-values ~1.0. (**D**) As for single cell SPRITE data, we assign a value of 1 or 0 to any of the 6617 SPRITE clusters from experiment that are or are not present in any of the structures of the population, by quantifying the SPRITE residual errors (*Methods and Supporting Information*). The resulting distribution of binary values is then compared with the experimental distribution, which only contain values of 1. The large p-values indicate that the null hypothesis can be accepted (confidence level *α* = 0.05) and that input and output are in fact drawn from the identical underlying probability distribution.

To showcase our data integration platform, we generated a population of 1,000 3D diploid genome structures of prolate ellipsoidal HFFc6 fibroblast cell nuclei (Extended Data Fig. 2a) at 200,000 base-pair resolution by integrating data from in situ Hi-C^58^, lamin B1 DamID^59^, HIPMap large-scale 3D FISH imaging^55^ and DNA SPRITE experiments^11^ (see Extended Data Fig. 2b–d for details of the optimization statistics). These structures are statistically consistent with all input data: (i) genome-wide Hi-C contact probabilities (genome-wide Pearson correlation: 0.98, average intra-chromosomal Pearson correlation: 0.98, average intra-chromosomal stratum-adjusted correlation coefficient^60^: 0.89; Fig. 2a,b and Supplementary Table 3); (ii) chromatin contact probabilities to the nuclear envelope (NE) from lamin B1 DamID experiments (Pearson correlation of 0.93; Fig. 2c,d); (iii) pairwise distance distributions for 51 pairs of loci from 3D HIPMap experiments (Pearson correlation of 1.0 of cross-Wasserstein distances Fig. 2e,f); and (iv) chromatin colocalizations for more than 6,600 chromatin clusters from SPRITE experiments (Fig. 2g and Extended Data Fig. 2d). Agreement between input experiments and predictions from optimized structures was further validated by *χ*^2^ goodness-of-fit tests (Methods and Extended Data Fig. 3).

**Fig. 3:**
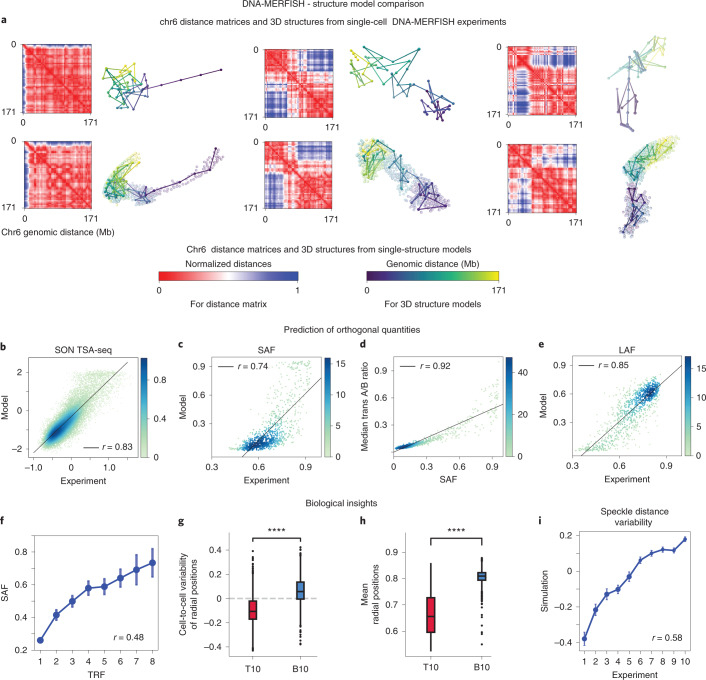
Genome structure population (from HDSF setup) correctly recapitulates imaging data, predicts a number of orthogonal quantities and provides interesting biological insights. **a**, Comparison of high scoring simulated structures of chromosome 6 and structures from the DNA-MERFISH dataset^17^. Each structure is plotted to the right of its normalized distance matrix; each row shows the corresponding structures in DNA-MERFISH experiments (top row) and IGM HDSF models (bottom row). Modeled structures at 200-kb base-pair resolution have a higher genomic coverage than the imaged genome structures. Genomic regions imaged in the experiment are shown in the models with opaque beads and are connected by opaque links, while genomic regions not imaged in the experiment are shown with translucent beads. **b**, Correlation between experimental^61^ and predicted SON TSA-seq data. **c**, Correlation between predicted and experimental SAF from DNA-MERFISH imaging; **d**, Experimentally observed correlation between SAF and *trans* A/B ratio from DNA-MERFISH imaging is nicely reproduced in our genome structures with high correlation. **e**, Correlation between experimental LAF from DNA-MERFISH imaging with predictions from our genome structure population. All scatterplots are colored according to the local density of points, and the Pearson correlation scores are annotated. TSA-seq correlations are genome wide, and DNA-MERFISH data correlations involve the 1,041 loci studied in the experiment by Su et al.^17^. **f**, Predicted SAFs of genomic regions show significant correlations (*r* = 0.49, *P* value ~ 0) with the transcription frequency (TRF) of genes from RNA-MERFISH imaging. TRF values are plotted in deciles. Error bars show mean values and standard deviations of predicted SAFs in each TRF range; number of SAF values used in the statistics (from left to right): 704, 79, 66, 61, 41, 27, 11, 10. **g**, Box plots of the cell-to-cell variability of radial positions for genomic regions containing actively transcribed genes with the 10% highest (T10) and 10% lowest (B10) transcription levels. **h**, Average radial positions of genomic regions containing actively transcribed genes with the 10% highest (T10) and 10% lowest (B10) transcription levels. Transcription levels for both **g** and **h** were taken from total RNA-sequencing experiments^62^. Comparison between T10 and B10 was performed using the Mann–Whitney two-sided tests, with *P* values ~ 0 for both **g** and **h**; the asterisks represent statistical significance of 0.0001. The box bounds indicate the interquartile range (Q3 − Q1) divided by the median, and Tukey-style whiskers extend to a maximum of 1.5 times the interquartile range beyond the box. Q3 and Q1 are the third and first quartiles of the distributions, respectively. Outliers are represented as dots. The number of B10 and T10 genomic regions used for the box plots is 1,253 and 1,296, respectively. **i**, Pearson correlation between experimental speckle distance cell-to-cell variability (Methods) from DNA-MERFISH imaging with predictions from our genome structure population, (*r* = 0.58, *P* value ~ 0). Error bars show standard deviations of speckle distance variability values in our models, in each experimental value decile. Number of values used in the statistics (from left to right): 99, 97, 103, 101, 94, 105, 100, 98, 101 and 101.

**Extended Data Fig. 4:**
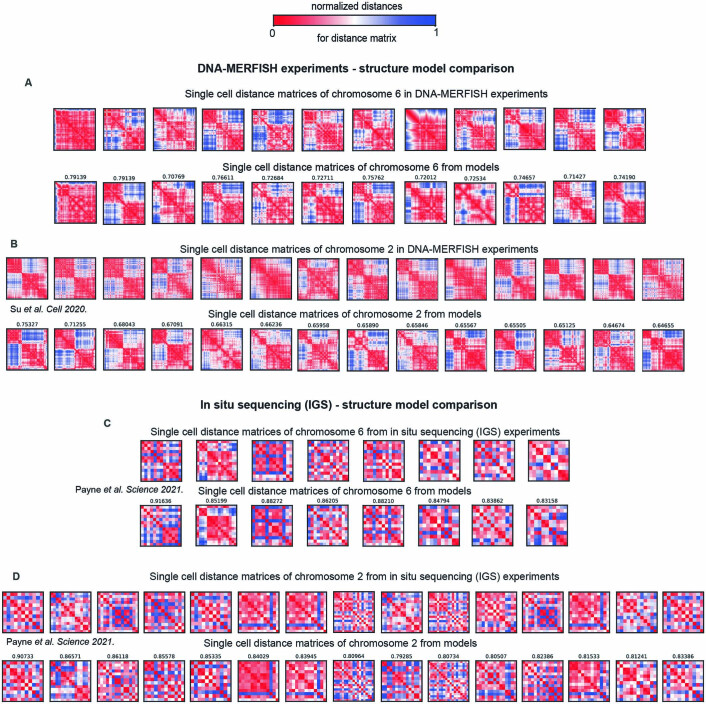
Validating chromosome structures from HDSF population with single cell structures from imaging experiments. (**AB**) Comparison of distance matrices of single cell chromosome 6 (**A**) and chromosome 2 (**B**) structures from simulated models and DNA-MERFISH imaging data^17^. Models reproduce a variety of folding patterns observed in experiment very efficiently. Numbers above the distance matrix indicate Pearson correlation between simulated and experimental distance matrices. (**CD**) Comparison of distance matrices of single cell chromosome 6 (**C**) and chromosome 2 (**D**) structures from simulated models and fibroblast in situ genome sequencing (IGS) imaged single cells^68^. Models reproduce a variety of folding patterns observed in experiment very efficiently. Numbers above the distance matrix indicate Pearson correlation between simulated and experimental distance matrices.

To evaluate the predictive value of our models, we must assess how well they predict independent experimental data, which were not used as input information. We first compared our chromosome structures with those from multiplex FISH imaging in a related IMR90 cell type^17^. Individual chromosome structures from DNA-MERFISH imaging^17^ show large structural variability, with distinctly different folding patterns between single-cell and homologous copies (Fig. 3a and Extended Data Fig. 4). We found good agreement between chromosome structures from our calculations and experiment (Methods), with several single-cell chromosome conformations found in our models with very similar distance matrix patterns. The range of conformational variability for chromosome 6 and chromosome 2 is nicely matched in our models for selected structures, as shown by the similarities for a range of distance matrices from the experiment and models (see Extended Data Fig. 4 for a more comprehensive comparison). For example, 72% of chromosome 6 structures in our models match to a structure from DNA-MERFISH experiments with an average distance matrix correlation of at least 0.5 or larger.

Next, we predicted the locations of nuclear speckles in each single-cell structure, following a previously described procedure^30^ (Methods). Based on the chromatin structural features, we first identified those chromatin regions with high propensity to be associated with nuclear speckles. We then determined in each model the highly connected spatial partitions formed by these chromatin regions. As we previously discovered, the geometric centers of each partition in a model serve as excellent approximations of nuclear speckle locations^30^.

The locations of predicted speckles together with the folded genome models were then used to predict experimental SON TSA-seq data (Methods and Fig. 1). SON TSA-seq is an experimental mapping method that determines, on a genome-wide scale, the median distances between any chromatin region and nuclear speckles^57^. Predicted SON TSA-seq data from our models agree remarkably well with experimental data^61^ (Pearson correlation 0.83; Fig. 3b). Moreover, our models confirm the previously described relationship between a chromatin region’s experimental SON TSA-seq value and its mean distance to the nearest speckle^57^.

We then used the predicted speckle locations to determine a gene’s speckle association frequency (SAF), defined as the fraction of models in which a chromatin region is in spatial association to a speckle (Methods and Fig. 1). A recent super-resolution microscopy study detected the same quantity for approximately 1,000 loci by DNA-MERFISH imaging^17^. The SAF prediction for these loci from our models shows excellent agreement with the experiments (Pearson correlation 0.71; Fig. 3c).

Moreover, we predicted for each chromatin region the median *trans* A/B ratio (Methods), defined as the ratio of A and B compartment chromatin forming interchromosomal interactions with the target loci. Predicted *trans* A/B ratios show good agreement with those determined by DNA-MERFISH experiments (Pearson correlation 0.66) and a strong correlation with the SAF (Pearson correlation 0.92; Fig. 3d), again confirming previous findings^17,30^.

The lamina-associated repressive chromatin compartment is usually located at the NE; thus, we used the location of the NE as a reference point to simulate lamin B1 TSA-seq data (Methods), which measures the mean distances of genomic regions to the nuclear lamina^57^. Moreover, we also calculated the lamina association frequency (LAF) for each genomic region (Fig. 1), which also shows excellent agreement with the LAF determined by super-resolution DNA-MERFISH imaging^17^ (Pearson correlation 0.84 for LAF; Fig. 3e). We also observed an inverse correlation between LAF and SAF (Pearson −0.77), confirming previous experimental observations.

**Extended Data Fig. 5:**
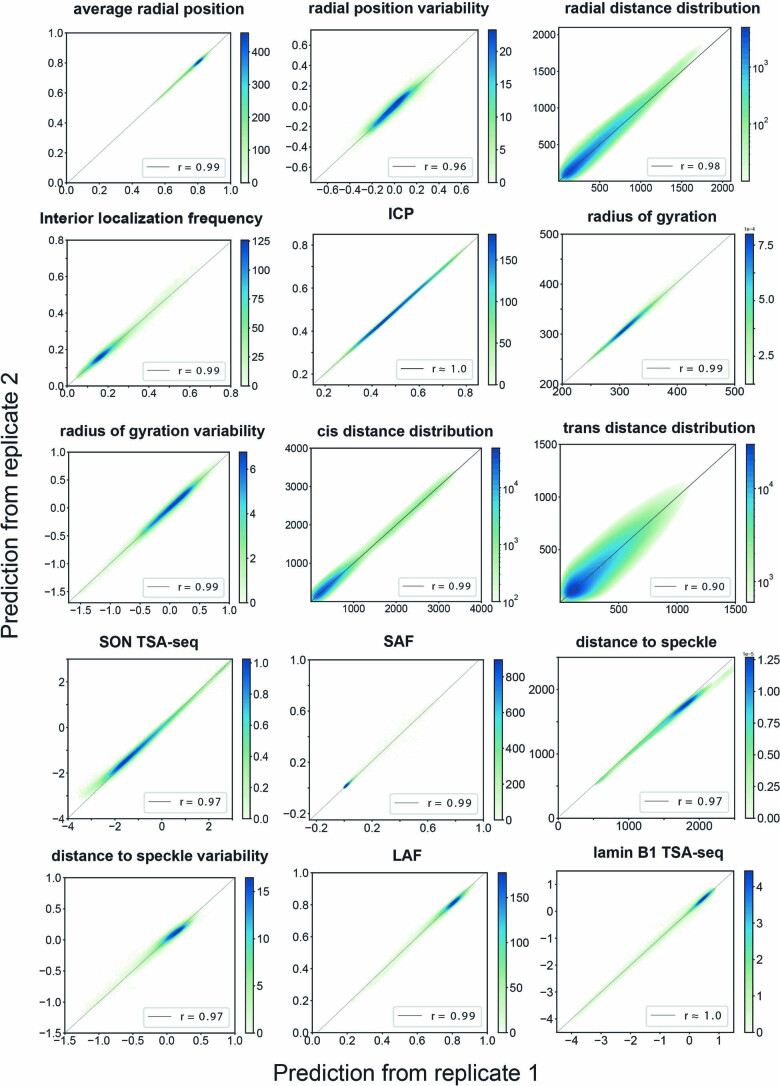
Reproducibility across IGM replicates. Reproducibility of 15 structural features in independent HDSF replicate calculations starting from different random starting configurations, see *Methods*. These features also include the reproducibility of cell-to-cell variability of several features from two independent population replicates. The high Pearson’s correlation values in each panel validate the robust reproducibility of all features (ICP = interchromosomal contact probability, SAF = speckle association frequency, LAF = lamina association frequency).

Overall, the accurate prediction of orthogonal observables assayed in independent experiments highlights the predictive power of our genome structures. We therefore can describe the nuclear microenvironment of each chromatin region by several structural features calculated from the models (Fig. 1 and Methods), namely: a chromatin region’s average radial position in the nucleus, the variability of its radial positions between single cells, the interior localization probability of a genomic region, the interchromosomal contact probability, the average local chromatin decompaction of the chromatin fiber and its variability across the population of models. Together with predicted SAF, LAF, *trans* A/B ratio and SON TSA-seq (Methods), we characterized each chromatin region by a total of 13 structural features, which define the structural microenvironment of each genomic region in the nucleus (Fig. 1). All structural features and chromosome structures are highly reproducible in independent replicate optimizations (Methods and Extended Data Fig. 5). For example, 80% of all structures of chromosome 6 in two replicate populations show almost identical structures with a correlation of at least 0.8 or larger between their corresponding distance matrices.

Studying the nuclear microenvironment of genomic regions (even at 200-kb resolution) provides useful information about the role of nuclear positions in gene function, information that is not otherwise easily accessible. For instance, we analyzed the link between a genomic region’s structural environment, in particular its nuclear location, with its gene expression propensity. We observed a significant correlation (Pearson 0.46, *P* value ~ 0) between the fraction of models a genomic region is in direct proximity to a nuclear speckle (SAF) and the fraction of single cells that show nascent mRNA transcripts for the corresponding genes in RNA-MERFISH experiments^17^; that is, its transcription frequency (TRF; Fig. 3f). This observation points to a favorable transcriptional microenvironment in the vicinity of nuclear speckles, and thus, confirms previous observations that point to a role of nuclear speckles in gene expression^11,57^.

We can then relate cell-to-cell variabilities of these features to functional properties. We observed a connection between the cell-to-cell variability of a genomic region’s nuclear position (Methods) with the expression level of genes located in these regions^30^. For instance, genomic regions containing the top 10% most highly transcribed genes showed substantially lower structural variability than regions containing the bottom 10% of transcribed genes (Fig. 3g; Mann–Whitney two-sided test, *P* value ~ 0, transcription data from RNA sequencing^62^). Thus, the most highly transcribed genes are located in genomic regions with the most stable nuclear structure. These regions also showed notably lower (more interior) average radial positions than genes present at low expression levels (Fig. 3h). We also found a significant correlation (Pearson 0.58, *P* value ~ 0) between our predicted cell-to-cell variability of a genomic region’s distance to the nearest speckle with that observed in DNA-MERFISH experiments (Fig. 3i).

Thus, structural features about nuclear locations of genomic regions can be directly linked to their functional potential in gene transcription. None of these structure-based findings would be possible through analysis of the input data alone.

### Multimodal data integration improves predictive power

**Fig. 4:**
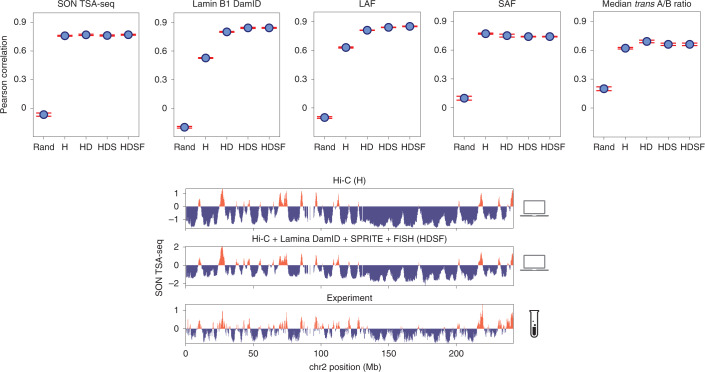
Predictive power and assessment of genome structures increases with integration of more data modalities. Top, Model accuracy for five different genome structure populations generated from different combinations of experimental input datasets: random chromosome territory (rand), Hi-C only (H), Hi-C + lamina DamID data (HD), Hi-C + lamina DamID + SPRITE (HDS) and Hi-C + lamina DamID + SPRITE + FISH (HDSF). The first and second plots show genome-wide Pearson correlation coefficients between model predictions and experimental data for experimental SON TSA-seq data and lamin B1 DamID. The third to fifth plots show Pearson correlations between experimental and predicted data for LAF, SAF and *trans* A/B ratio for 1,041 imaged loci from DNA-MERFISH imaging experiments^17^. Error bars were computed as the standard deviation of the Pearson correlation across three independent population replicates (Methods). Data are presented as mean values ± standard deviation. Bottom plots show the comparison between experimental^61^ and predicted SON TSA-seq profiles of chromosomes 2 (top and bottom, respectively). Predicted profiles are shown for structure populations generated with setups H and HDSF (Methods).

We next investigated how different combinations of data influence model accuracy. We generated four genome populations, each with different combinations of experimental data, and assessed their accuracy by comparing predicted SON TSA-seq data, lamina DamID data, SAF, LAF and median *trans* A/B ratios with those available from experiments (Methods and Fig. 4). For reference, we also assessed a population of random chromosome territories constrained within the nuclear volume.

Interestingly, models from Hi-C data alone (setup H) reproduce SON TSA-seq data and SAF already with high accuracy, while lamin B1 DamID and LAF show relatively poor performance (Fig. 4), which is likely related to the flat ellipsoidal shape of the HFF nucleus. Our previous studies using GM12878 cells, with a spherical nucleus, could predict both lamina TSA-seq and lamin B1 DamID data with higher accuracy from Hi-C data alone^30^. When Hi-C and Lamina DamID data (setup HD) were combined, predictions of TSA-seq, DamID data, SAF and LAF greatly improve (Fig. 4).

**Extended Data Fig. 6:**
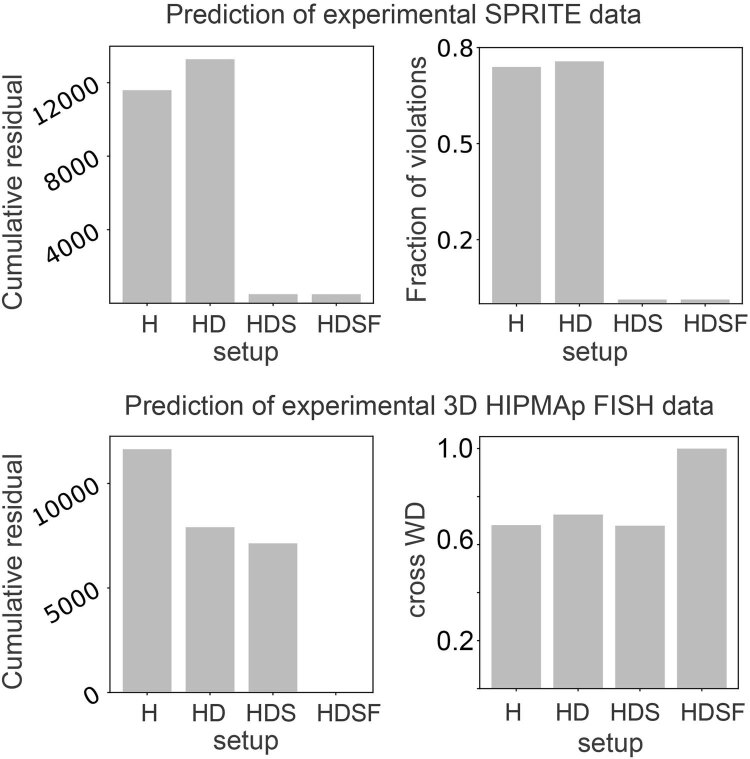
Prediction of experimental SPRITE and FISH data in HFFc6 H, HD, HDS, HDSF populations. (Top panels) SPRITE^11^ cumulative residual (left) and fraction of violated SPRITE restraints (right) for each of the data-driven populations discussed in Fig. 4. Lamina DamID restraints tend to stretch the genome towards the lamina, whereas SPRITE restraints squeeze the targeted loci close to one another: an optimal balance is only found when both data modalities are simultaneously integrated, for example, populations HDS and HDSF. (Bottom) FISH cumulative residual (left) and cross WD score (right). The cumulative residual is defined as the sum of the residual errors *η* for all violations; the cross WD score is the Pearson correlation between two cross WD sets (see *Methods* and *Supporting Information*). FISH distributions^55^ are gradually better predicted with increasing amount of data and most efficiently recapitulated in population HSDF only, as suggested by a cross WD score of 0.999 and the smallest cumulative residual.

Combining SPRITE colocalization clusters and 3D FISH distance distributions with Hi-C and lamin B1 DamID, input information slightly improved correlation scores for TSA-seq and DamID data, even though the total number of spatial restraints from DNA SPRITE and FISH data were an order of magnitude smaller than those from Hi-C and lamina DamID (Extended Data Fig. 2d). Models HDS and HDSF recapitulated MERFISH imaging data well, recapitulated 3D FISH and SPRITE data, while also showing excellent predictability for TSA-seq and DamID data (Fig. 4 and Extended Data Fig. 6). Overall, the steady improvement of model accuracy with an increasing amount of input data highlights the benefits of multimodal over unimodal data integration in generating realistic and highly predictive structures.

### Systematic assessment of comprehensive data integration using synthetic data

**Fig. 5:**
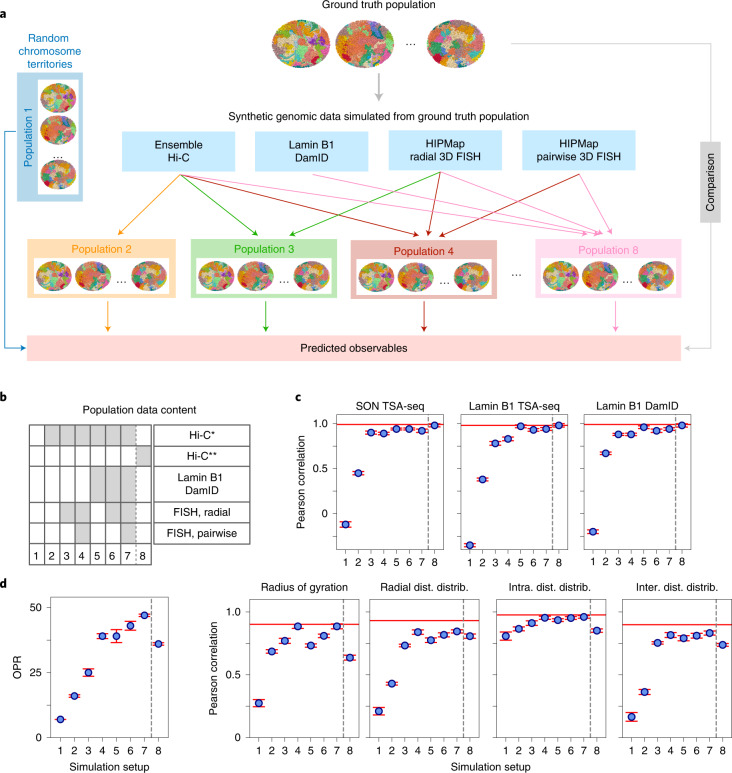
Systematic data integration via synthetic genomic data. **a**, Schematic of the assessment process. Information corresponding to Hi-C, lamina DamID and radial and pairwise FISH data was simulated from a structure population that serves as a reference ground truth. Eight different genome structure populations were calculated from different combinations of synthetic data. Independent structural features were calculated from each population and compared with the ground truth reference to assess the accuracy of the models. **b**, Combinations of synthetic data included in the eight different input setups (columns). Gray boxes indicate the presence of a synthetic data type in the input setup. Hi-C* and Hi-C** indicate two differently perturbed Hi-C maps. In Hi-C* only, interchromosomal contact frequencies were scaled down by a factor of 2. In Hi-C** only, intra-chromosomal contact frequencies were scaled down by a factor of 2. **c**, Accuracy of models was estimated for each input setup using the Pearson correlations between predicted structural features and those in the ground truth reference. Structural features included SON and lamin B1 TSA-seq data, lamin B1 DamID data, the radius of gyration for chromosomes, distributions of chromatin radial positions, distributions of intra-chromosomal distances, and distributions of inter-chromosomal distances. Baseline predictions from the correct (non-perturbed) Hi-C-only simulation are indicated with a red horizontal line. **d**, OPRs for all setups. The OPR for setup *s* was calculated as follows: $\mathrm{OPR}_s = \mathop {\sum}\nolimits_{f = 1}^7 {(9 - \mathrm{rank}_f^s)}$, where rank_*f*_^*s*^ is the rank of setup *s* in assessment of feature *f*. rank_*f*_^*s*^ is 1 for the top-ranking setup, and 8 for the poorest performing setup for feature *f*. Therefore, OPRs can range from 56 (best performance in all feature assessments) to 8 (poorest performance in all feature assessments). Error bars in **b**–**d** for each setup were estimated from three independent population replicates (Methods). Data are presented as mean values ± standard deviation.

To perform a thorough assessment of multimodal data integration, we regarded a structural population as a ‘ground truth’ reference, from which a variety of synthetic data can be simulated (Methods and Fig. 5a). Models were then generated from different combinations of synthetic data, to facilitate the comparison of their predictive power on 3D genome architecture. Note that model assessment depends on the structural features being explored, and a ground truth allows a more comprehensive model validation based on a larger number of structural observables that are accessible. Moreover, we can simulate different input data at variable levels of information content to better assess their influence on model quality.

We chose population H (Fig. 4) as the ground truth structure population, from which we generated the synthetic datasets, including genome-wide contact frequencies (that is, Hi-C data), contact frequencies between loci and the NE (that is, lamin B1 DamID data), and a randomly chosen subset of 1,000 radial and 1,000 pairwise distance distributions (that is, HIPMap 3D FISH datasets; Methods and Fig. 5a). These datasets represent idealized data sources, and were combined into seven different input data setups. Models were then generated for all data setups, each containing different combinations of synthetic data (Fig. 5b).

We quantitatively assessed model accuracy with the following structural properties (Fig. 5c): (i) the distribution of radial positions for each chromatin region, (ii) the distributions of pairwise distances between chromatin loci in *cis* and *trans*; (iii) the distribution of the radius of gyration for each chromosome; (iv) SON TSA-seq data; (v) lamin B1 TSA-seq data; and (vi) lamin B1 DamID data. We used the cross-Wasserstein distance to measure the similarity between two probability distributions (for features i–iii); quantities (iv–vi) were assessed by their Pearson correlations with the corresponding ground truth features (Methods). Finally, for each setup, an overall performance rank (OPR) was determined as the total sum of ranks for all individual feature assessments (Fig. 5d).

Models generated from simulated contact frequencies naturally reproduce with high accuracy the ground truth features. To better substantiate our assessment of data integration performance, we manipulated the simulated Hi-C data by scaling down the interchromosomal contact probabilities by a factor of two and used the resulting ‘perturbed’ contact map (labelled Hi-C*) as input for all model populations instead.

Structures generated from perturbed Hi-C^*^ data alone (setup 2) showed poor performance with low correlations of ground truth features, except for intra-chromosomal distance distributions (Pearson correlation 0.79; Fig. 5c). We then generated another perturbed Hi-C** dataset, in which interchromosomal interactions remain untouched, while probabilities of intra-chromosomal interactions were scaled down by a factor of 2 (setup 8). Models generated using this dataset predicted with good accuracy all ground truth features related to the global nuclear architecture, such as SON TSA-seq, lamin B1 TSA-seq and lamina DamID signals (Pearson correlations > 0.98) as well as radial distributions of chromatin regions with substantially higher accuracy than setup 2 Hi-C* (Fig. 5c). In contrast, setup 8 showed slightly higher accuracy than setup 2 for chromosomal properties, such as the radius of gyration. It is noteworthy that intra-chromosomal distance distributions were still well reproduced in comparison to setup 2, which indicates that scaling down intra-chromosomal contacts has a less detrimental effect than interchromosomal contacts. These results showcase the surprisingly dramatic loss of information when *trans* contact probabilities are underestimated in Hi-C data, which generally have very low contact probabilities to begin with. Reducing interchromosomal interactions further will lead to the loss of information about the global genome architecture. Reducing relatively high-frequency intra-chromosomal contact probabilities will have a smaller impact, as sufficient information about intra-chromosomal chromatin interactions is still retained in the dataset.

**Fig. 6:**
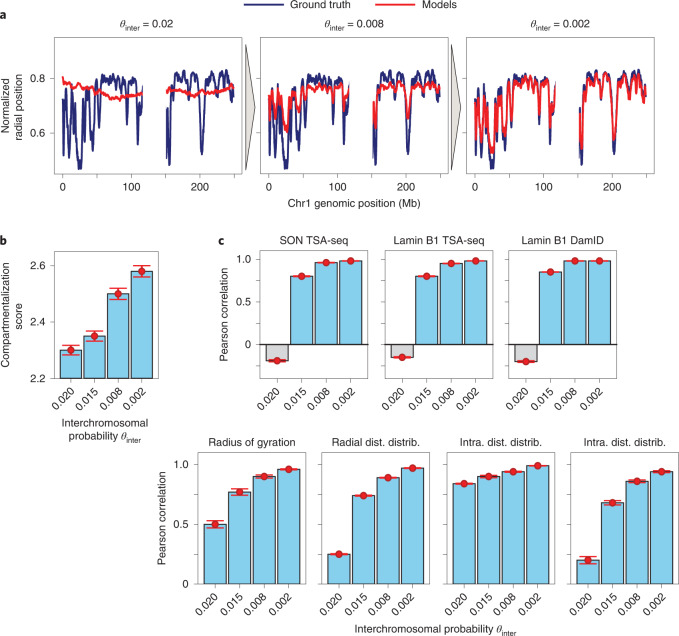
Low-probability interchromosomal contacts greatly affect model predictivity. We compared the accuracy of a structure population generated from unperturbed Hi-C data as a function of the lowest interchromosomal contact probability value included in the modeling. The probabilities are labeled as *θ*_inter_ (Methods). **a**, Mean radial positions plotted for chromatin regions in chromosome 1 from structures in the ground truth reference population (dark blue) and structures calculated from three representative setups (red) that included interchromosomal contacts with gradually decreasing contact probabilities: *θ*_inter_ = 0.02, 0.008 and 0.002. Characteristic radial profiles seen in the ground truth (Fig. 1) were only correctly reproduced when contacts were included with probabilities of at least 0.2%. From left to right, *θ*_intra _= 0.02, 0.008 and 0.002. **b**, The A/B compartmentalization score for each setup, with error bars representing the standard deviation of the underlying distribution (Methods): compartmentalization increased as more low-frequency interchromosomal contacts were included in the modeling. **c**, The Pearson correlation value between the ground truth and simulations of the same seven structural observables discussed in Fig. 5 for *θ*_inter_ = 0.020, 0.015, 0.008 and 0.002. Gray boxes indicate negative correlation values. Structural quantities experienced a substantial correlation increase when low-probability contacts were included, indicating that overall model predictivity increases dramatically. Error bars for each setup were estimated from three independent replicate calculations (Methods); data in **b** and **c** are presented as mean values ± standard deviation.

To further assess the relevance of interchromosomal interactions, we generated four structure populations from (unperturbed) Hi-C data that included interchromosomal contacts only if their contact probability was larger than a given cutoff *θ*_inter_, which is gradually decreased (Methods). Interestingly, good predictive models can only be generated when interchromosomal contacts with very low probabilities are included (Fig. 6). For instance, radial profiles are only reproduced with low residual errors if relatively ‘rare’ contact events are included, that is, probabilities corresponding to only 2 contact events per 1,000 structures (Fig. 6a). The chromatin compartmentalization score, which measures the spatial segregation between chromatin in the active A compartment from the inactive B compartment^63^ (Methods), also steadily increased when interchromosomal contacts with low contact probabilities were added (Fig. 6b). Thus, the large number of low-probability interchromosomal interactions, which define relatively ‘rare’ contact events per chromatin region, are essential for accurate genome structure modeling and for correct predictions of genome-wide SON TSA-seq, lamin B1 TSA-seq and lamin B1 DamID data (Fig. 6c). Overall, these results further underline the important role of *trans* interactions in predicting the correct global genome architecture in our models. Hi-C experimental conditions can influence fragment lengths, ligation efficiencies and thus the amount of informative interchromosomal proximity information captured by ligations. Hi-C variants, such as MicroC^6^, capture local short-range chromatin interactions at higher resolution, while the fraction of long-range and interchromosomal interactions is reduced. It is therefore of interest to test if additional orthogonal data sources can compensate for reduced levels of informative interchromosomal interactions.

**Extended Data Fig. 7:**
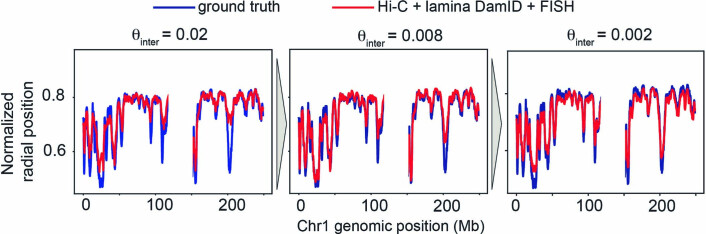
Relevance of low frequency inter-chromosomal contacts. (Unperturbed) Hi-C, lamina DamID and 1000 radial and 1000 pairwise FISH distance distributions extracted from the ground truth (Fig. 5) are used to generate a population of structures. The predicted radial profiles for chromosome 1 are compared with the underlying ground truth at different stages of the optimization process. Specifically, lamina DamID and FISH data have been all added up to the final thresholds *λ*_*final*_ and *t*_*final*_, and low frequency inter chromosomal contacts added up to probability *θ*_*inter*_ = 0.02 (left) and *θ*_*inter*_ = 0.008 (right). Radial profiles are better reproduced in multi-modal Hi-C + lamina DamID + FISH models at *θ*_*inter*_ = 0.02 than they are in Hi-C only models with the same setup (Fig. 6A**)**, and then refined by lowering the contact probability *θ*_*inter*_. This provides alternative evidence that independent data sources can account for missing information; here, inter chromosomal contacts with probability smaller than 0.008. (*θ*_*inter*_ = 0.02, 0.008).

Combining lamin B1 DamID as well as radial and pairwise distance distributions from 3D FISH experiments with the biased Hi-C* data (setup 7) produced models with high predictive power and similar accuracy for all structural features as models generated with unmodified original Hi-C data (Fig. 5c). The OPR increased monotonically with increasing amounts of added data (setups 3–7; Fig. 5d). Therefore, orthogonal data modalities appear to compensate for systematic errors affecting one of the data types (here, underrepresentation of interchromosomal contacts; Extended Data Fig. 7).

The steady improvement in model accuracy with increasing data is not only due to those features being directly restrained by the added data (which is only a small portion of all degrees of freedom), but also due to cooperative effects acting on the entire genome: each newly added data modality makes already included data more informative. This is due to the specific nature of our iterative optimization process, which reduces data ambiguity by selecting the best of a set of alternative restraints assignments, based on the current genome structures at a given iteration (Methods and Supplementary Information). For instance, if newly added information about a gene’s radial position restricts its nuclear locations, it will also make certain non-native chromatin contacts less likely, which in turn will lower the change for that gene to be wrongly selected in non-native Hi-C contact-restraint assignments. An analogy is a crossword puzzle, where gradually filling in interconnected words reduces the ambiguity of missing word solutions. Adding a data modality to our modeling process reduces, in a similar way, the ambiguity of restraints assignments of all other data types, thus making these data more informative.

Our simulations showed that adding FISH radial distributions for 1,000 loci (setup 2 to setup 3) improved prediction accuracy of radial distributions for all genes (not only those being actively restrained), as well as genome-wide SON and lamin B1 TSA-seq signals, and even interchromosomal gene distance distributions, although the radial FISH data did not contain any bivariate information (Fig. 5c).

Models generated from Hi-C* and simulated DamID data (setup 5) outperformed models from Hi-C* data and FISH radial distributions of 1,000 loci (setup 3). However, adding information for 1,000 pairwise FISH distance distributions (setup 4) produced models as accurate as those in setup 5.

**Extended Data Fig. 8:**
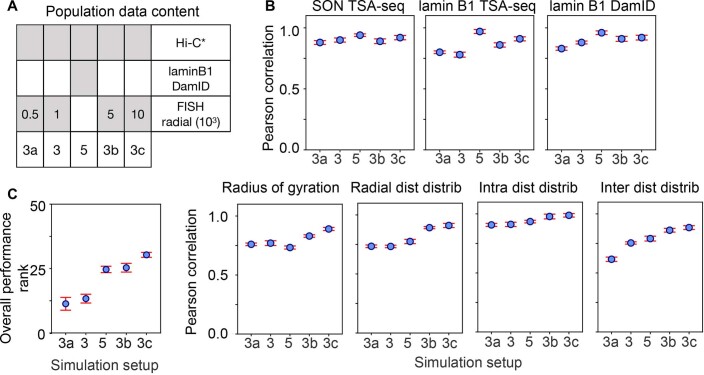
Comparing information content of lamina DamID data against increasingly larger radial distance distribution FISH data sets. Additional Hi-C* and radial FISH only populations (3a, 3b and 3c) are analyzed and compared with previous Hi-C*-radial FISH population 3 and Hi-C*-DamID only population 5 from Fig. 5. (**A**) The four populations with FISH data differ in the number of radial distributions used in the input (500, 1,000, 5,000 and 10,000). (**B**) The seven quantities from Fig. 5C are predicted for each population and compared with the ground truth. (**C**) The overall performance rank for these five populations indicates that a sufficiently large sample of radial distance distributions can match and outperform the information provided by lamina DamID data. Error bars for each setup were estimated from three independent population replicates (see *Methods*); data in panels (B) and (C) are presented as mean values +/− standard deviation.

The information equivalence of datasets depends naturally on the amount of data. For instance, using radial distributions of all chromatin loci would render lamina DamID data redundant. We therefore assessed (Hi-C* + radial FISH data) class models that contain increasing numbers of FISH probes. Our results confirm that, at a critical number of probes, models from Hi-C* and radial FISH data become more informative than those from Hi-C* and lamina DamID data (setup 5; Extended Data Fig. 8). Of course, these observations are made in an idealized case, and only serve as a conceptual point. The true information content of data depends on systematic errors in the experimental data, such as potential distortions due to cell fixations and other treatments in FISH experiments, as well as the base-pair resolution of the chromatin fiber representation. Also, radial positions (instead of distance to the nuclear lamina) may be an inadequate description for highly irregular nuclear shapes that vary in size. In future, actual microcopy 3D images, instead of positional metadata, should be used in the modeling process to overcome some of these issues.

## Discussion

We introduced a robust pipeline for multimodal data integration to determine 3D structures of whole diploid genomes. These structures revealed a wealth of information about the structural organization of genomes over multiple length scales, along with dynamic variabilities of structural features between individual cells. Collectively these features define the nuclear microenvironment of genes on a genome-wide scale, which can be directly linked to their functional potential in gene transcription and subnuclear compartmentalization^43^. Our method therefore provides a useful analytical tool for comparative genome structure analysis, which could link changes in a gene’s structural organization between different cell types (or during developmental processes) with underlying functional changes. Moreover, the structures generated by our method also predict a host of orthogonal experimental data, including SON TSA-seq data, speckle and lamina association frequencies and *trans* A/B ratios as determined by DNA-MERFISH experiments, and reproduce chromosomal structures from super-resolution imaging experiments. These predictions could serve as first approximations to data otherwise only available through experiments with considerable added effort.

We tested the proficiency of our approach by studying the diploid genome structures of human HFFc6 cells by integrating data from Hi-C, lamin B1 DamID, 3D HIPMap FISH and SPRITE experiments. We systematically assessed the accuracy of models generated from different combinations and amount of data types. Model accuracy steadily improves with increasing amounts of data and is maximal when data integration is multimodal, indicating that single data sources might not fully capture all information about a genome’s structural organization. Moreover, orthogonal data sources can compensate for systematic biases and missing information in some data types. For instance, a biased Hi-C dataset with artificially reduced chromatin interaction frequencies shows substantially lowered accuracy. However, combining this biased dataset with additional information from lamina DamID and 3D FISH experiments recovers structures with almost identical accuracy to those generated by the unbiased Hi-C data. The improvement of performance can partly be explained by cooperative effects. Adding a complementary data type to the input set can reduce ambiguity in other data, thus making already included data more informative.

Also, different combinations of orthogonal data sources can produce models with similar levels of high accuracy and thus share similar information content. For instance, the combination of Hi-C with lamina DamID data can produce similarly accurate structures than a combination of data from Hi-C and 3D FISH experiments, given that a critical number of FISH probes is considered. Therefore, the method does not rely on a specific combination of data to produce models with high predictive values.

Interestingly, our work also underlines the essential role of low-probability interchromosomal interactions for accurate data-driven predictions of genome organizations. The multitude of relatively ‘rare’ contact events are crucial for accurate predictions of radial gene positions and overall chromatin compartmentalization. It is not sufficient to consider only the most frequent interactions in the modeling process. However, if datasets are compromised by a lack of sufficient information about *trans* interactions, additional orthogonal data sources can compensate for a reduced level of information.

In future, our approach will be expanded to incorporate 3D imaging data into the modeling process also, which will consider variations in nuclear shapes between individual cells and exclude volumes for some nuclear bodies. We expect that these additions will further improve the quality of models. Due to its modular organization, our software platform is readily suited for incorporating new volumetric microscopy data

In summary, here we showed that our method provides a useful tool for multimodal data integration to produce genome structure models with high predictability. Our software implementation is publicly available, widely applicable to other cell types and can be tailored to include new experimental data types.

## Methods

Our population-based modeling approach uses a probabilistic framework to generate a large number of 3D genome structures (that is, the structure population) statistically consistent with all input data (that is, Hi-C, lamin B1 DamID, 3D FISH and SPRITE). Structures are generated by a deconvolution of ensemble data (Hi-C, lamin DamID and 3D FISH) and incorporation of single-cell data (SPRITE) into a population of individual diploid genome structures that represent the most likely approximation of the true population of genome structures, given all the available data. The structure optimization problem is formulated as a maximum likelihood estimation problem using an iterative optimization scheme.

### Genome representation

Chromosomes are segmented into genomic regions of 200-kb DNA sequence length, each represented by chromatin domains with spherical volume. Each chromatin domain is defined by an excluded volume with a sphere radius *r*_0_ = 118 nm, which guarantees a 40% volume occupancy of the diploid genome in the nucleus. In a diploid genome, each autosome genomic region has two homologous chromatin domain copies. Overall, the diploid genome is represented by a total of *N* = 29,838 chromatin domains. The nuclear shape is modeled as a prolate ellipsoid of semiaxes (*a*, *b*, *c*) = (7,840 nm; 6,470 nm; 2,450 nm); Extended Data Fig. 2a). The semiaxes’ lengths are based on the estimates from Seaman et al.^64^.

Our model, the structure population, is defined as a set of *S* diploid genome structures ***X*** = {***X***_1_,…,***X***_*S*_}; a genome structure ***X***_*S*_ is a set of 3D vectors representing the center coordinates of each chromatin domain ${{{\boldsymbol{X}}}}_s = \{ {{{\vec{\boldsymbol x}}}}_{is}:{{{\vec{\boldsymbol x}}}}_{is} \in {\Bbb R}^3,i = 1,2, \ldots ,N\}$, with *N* as the total number of all chromatin domains in the diploid genome. The variable *H* indicates the total number of genomic regions, that is, the number of domains when homologous copies are not distinguished.

Note that capital letter indices, such as *I* and *J*, relate to domains without distinguishing between two homologous copies, while lowercase indices *i*, *i’* and *j*, *j’* distinguish between the two copies, when applicable (sex chromosomes only come in one copy).

### Data source representation

We integrate data from four experimental methods, namely in situ Hi-C^58^ and lamin B1 DamID^59^, high-throughput HIPMap 3D FISH^55^ and SPRITE^11^.

Data types are categorized into three classes depending on the number of genomic loci involved. For instance, data that inform on the coordinates of only a single genomic locus will be univariate, such as the radial distance of a locus from radial FISH data or a normal distance to the nuclear lamina from lamina DamID data. Bivariate data inform on pairs of genomic loci, for instance, distances between pairs of loci from 3D FISH experiments or contacts between pairs of loci from Hi-C experiments. Multivariate data define relationships between more than two loci, for example, knowledge about colocalization of a set of loci in single cells from SPRITE experiments.

Most experiments, such as Hi-C and Lamina DamID, provide data that are averaged over a large population of cells, and so they cannot reveal which contacts coexist in which single-cell structure. Moreover, unphased data cannot discriminate between homologous chromosome copies. To represent the missing information at single-cell level and to distinguish homologous chromatin domain copies, we introduce indicator tensors ${\frak{D}}^ \ast = \left\{ {{{{\mathcal{D}}}}_k^ \ast |k = 1, \ldots ,K} \right\} = \{ {{{\boldsymbol{B}}}},{{{\boldsymbol{V}}}},{{{\boldsymbol{F}}}},{{{\boldsymbol{W}}}},{{{\boldsymbol{R}}}}\}$ as latent variables that augment missing information in data variables ${\frak{D}} = \left\{ {{{{\mathcal{D}}}}_k|k = 1, \ldots ,K} \right\} = \{ {{{\boldsymbol{U}}}},{{{\boldsymbol{E}}}},{{{\boldsymbol{M}}}},{{{\boldsymbol{A}}}},{{{\boldsymbol{T}}}}\}$, respectively (Supplementary Table 1).

#### Chromosome conformation capture

Hi-C data are expressed as a contact probability matrix ***A*** = (*a*_*IJ*_)_*H*×*H*_ where 0 ≤ *a*_*IJ* _≤ 1 is the contact probability between the genomic regions *I* and *J*^44^. The contact probability matrix ***A*** is incomplete and does not contain the detailed information about which of the homologous domain copies (*i* and *i*′ for genomic region *I*, and *j* and *j*^'^ for *J*) are in contact, nor does it provide information about structures of the population in which a contact is present. To complement every cell’s contact information, we introduce the contact indicator tensor ***W*** = (*w*_*ijs*_)_*N*×*N*×*S*_, which is a latent binary-valued third-order tensor specifying the contacts between chromatin domains *i* and *j* for each homologous copy in each structure of the population. *w*_*ijs* _= 1 indicates that a contact between chromatin loci *i* and *j* is present in structure *s*, while *w*_*ijs* _= 0 indicates that such a contact is not present. ***W*** is a detailed expansion of ***A*** at the diploid representation and single-cell level with a dependence relationship ***X*** → ***W*** → ***A***.

#### Lamina DamID

Lamina DamID data are expressed by the tensor ***E*** = (*e*_*I*_)_*H*_, where 0 ≤ *e*_*I* _≤ 1 is the probability that genomic region *I* is in contact with the lamina at the NE, which is derived from lamin B1 DamID data, following a similar notation as used by Li et al.^37^.

To complement information about homologous domains in single structures, we introduce the binary-valued latent tensor ***V*** = (*v*_*is*_)_*N*×*S*_, which indicates whether the *i*-th chromatin domain is in contact with nuclear lamina in the *s*-th structure (*v*_*is* _= 1) or not (*v*_*is* _= 0). ***V*** is a detailed expansion of ***E*** at the diploid representation and single-cell level with a dependence relationship ***X*** → ***V*** → ***E***.

#### 3D FISH HIPMap

Data from 3D FISH HIPMap experiments are divided into two sets of data: (i) univariate data about the radial positions of genomic loci, and (ii) bivariate data providing information about the distributions of distances between pairs of genomic loci. Large-scale FISH data provide the probability distributions of pairwise distances between genomic loci and probability distributions of radial positions of genomic loci in the nucleus. Probability distributions of both radial and pairwise distances are discretized into *Q* bins, which equally span the nuclear dimension. For convenience, we can assume bins are disjoint and that any distance can be assigned to only one bin.

#### 3D FISH radial positions

We express radial 3D FISH data with the tensor ***U*** = (*u*_*Iq*_)_*H*×*Q*_, with *H* as the number of genomic regions and *Q* as the total number of distance bins. *u*_*Iq*_ is the probability that the radial position of genomic locus *I* falls into the range defined by ${{{\mathcal{B}}}}_q = \left[ {d_q,d_{q + 1}} \right)$, with *d*_*q*_ as the lower bound and *d*_*q+1*_ as the upper bound for radial positions in bin *q*.

To complement missing information about single-cell structures and homologous domain copies, we introduce the binary-valued latent tensor ***B*** = (*b*_*iqs*_)_*N*×*Q*×*S*_, which indicates whether the *i*-th chromatin domain in structure *s* has a radial position in the range defined by bin ${{{\mathcal{B}}}}_q = \left[ {d_q,d_{q + 1}} \right)(b_{iqs} = 1)$ or not (*b*_*iqs*_ = 0). ***B*** is a detailed expansion of *U* at the diploid representation and single-cell level with a dependence relationship ***X*** → ***B*** → ***U***.

#### 3D FISH distance distributions

We express 3D FISH pairwise distance data by the tensor ***M*** = (*m*_*IJq*_)_*H*×*H*×*Q*_, where *m*_*IJq*_ is the probability that genomic loci *I* and *J* have a distance in the range defined by bin ${{{\mathcal{B}}}}_q = [d_q,d_{q + 1})$. The binary-valued tensor ***F*** = (*f*_*ijqs*_)_*N*×*N*×*Q*×*S*_ complements the missing information about homologous domain copies and single cells and thus indicates whether the spatial distance between the *i*-th and *j*-th chromatin domains in structure *s* falls in the range of ${{{\mathcal{B}}}}_q = \left[ {d_q,d_{q + 1}} \right)$ (*f*_*ijqs*_ = 1) or not (*f*_*ijqs*_ = 0). ***F*** is a detailed expansion of ***M*** at the diploid representation and single-cell level with a dependence relationship ***X*** → ***F*** → ***M***.

#### SPRITE

The SPRITE data provide information about the number and identity of genomic regions colocalized in a single-cell structure. We expressed these SPRITE clusters by a collection of tensors {***T***^*n*^} = $\left(t_{I_1, \ldots , I_n} \right)_{H^n}$, where *n* is the number of genomic regions in a SPRITE cluster. Each tensor entry $t_{I_1, \ldots, I_n}$, derived from single-cell SPRITE data is the probability of genomic regions *I*_1_,…,*I*_*n*_ to be colocalized in a single structure of the population $t_{I_1, \ldots, I_n} = 1$ or not $t_{I_1, \ldots, I_n} = 0$. All clusters of *n* regions are described by the multidimensional tensor ***T***^*n*^, and we will use the notation *C*_*n*_ to indicate any of those clusters *n* genomic loci. Summing all the clusters of any size is indicated then by the notation $\mathop {\sum}\nolimits_n {{\sum} {C_n} }$.

The latent indicator tensor ***R***^*n*^ = $\left( r_{i_1, \ldots , i_n,s}\right)_{N^n \times S}$, where $r_{i_1, \ldots , i_n,s}$ distinguishes homologous domain copies, complements the information by indicating whether chromatin domains (different copies are distinguished) {*i*_1_,…,*i*_*n*_} are colocalized in structure *s* $r_{i_1, \ldots , i_n,s} = 1$ or not $r_{i_1, \ldots , i_n,s} = 0$. ***R***^*n*^ is a detailed expansion of ***T***^*n*^ at the diploid representation and single-cell level with a dependence relationship ***X*** → ***R***^*n* ^→ ***T***^*n*^

In the following, we will collectively indicate the family of ***T***^*n*^ and ***R***^*n*^ tensors with ***T*** and ***R***, respectively, as ***T*** = {***T***^*n*^} and ***R*** = {***R***^*n*^}.

### Probabilistic formulation of maximum likelihood problem

We introduced a set of data variables $\left\{ {{{{\mathcal{D}}}}_k|k = 1, \ldots 5} \right\} = \{ {{{\boldsymbol{U}}}},{{{\boldsymbol{E}}}},{{{\boldsymbol{M}}}},{{{\boldsymbol{A}}}},{{{\boldsymbol{T}}}}\}$ and a set of indicator tensors $\left\{ {{{{\mathcal{D}}}}_k^ \ast |k = 1, \ldots ,5} \right\} = \{ {{{\boldsymbol{B}}}},{{{\boldsymbol{V}}}},{{{\boldsymbol{F}}}},{{{\boldsymbol{W}}}},{{{\boldsymbol{R}}}}\}$ as latent variables that augment missing information in data variables to distinguish homologous chromatin domain copies and in single cells. Given $\left\{ {{{{\mathcal{D}}}}_k} \right\}$, we aimed to estimate the structure population model ***X*** such that the likelihood $P\left( {\left\{ {{{{\mathcal{D}}}}_k} \right\},\left\{ {{{{\mathcal{D}}}}_k^ \ast } \right\}|{{{\boldsymbol{X}}}}} \right) = P\left( {{{{\boldsymbol{U}}}},{{{\boldsymbol{E}}}},{{{\boldsymbol{M}}}},{{{\boldsymbol{A}}}},{{{\boldsymbol{T}}}},{{{\boldsymbol{B}}}},{{{\boldsymbol{V}}}},{{{\boldsymbol{F}}}},{{{\boldsymbol{W}}}},{{{\boldsymbol{R}}}}|{{{\boldsymbol{X}}}}} \right)$ is maximized. The statistical dependence relationship between data sources and latent variables in an optimized structure population is ${{{\boldsymbol{X}}}} \to {{{\mathcal{D}}}}_k^ \ast \to {{{\mathcal{D}}}}_k,\forall k$, because $\left\{ {{{{\mathcal{D}}}}_k^ \ast } \right\}$ is a detailed expansion of $\left\{ {{{{\mathcal{D}}}}_k} \right\}$ at the diploid and single-structure representation of the data and ***X*** is the structure population consistent with $\left\{ {{{{\mathcal{D}}}}_k^ \ast } \right\}$. Therefore, the likelihood $P\left( {\left\{ {{{{\mathcal{D}}}}_k} \right\},\left\{ {{{{\mathcal{D}}}}_k^ \ast } \right\}|{{{\boldsymbol{X}}}}} \right)$ can be expanded to $P\left( {\left. {\left\{ {{{{\mathcal{D}}}}_k} \right\}|\left\{ {{{{\mathcal{D}}}}_k^ \ast } \right\},{{{\boldsymbol{X}}}}} \right)P\left( {\left\{ {{{{\mathcal{D}}}}_k^ \ast } \right\}|{{{\boldsymbol{X}}}}} \right.} \right)$ and therefore

$$\begin{array}{l}P\left( {{{{\boldsymbol{U}}}},\boldsymbol{E},{{{\boldsymbol{M}}}},{{{\boldsymbol{A}}}},{{{\boldsymbol{T}}}},{{{\boldsymbol{B}}}},{{{\boldsymbol{V}}}},{{{\boldsymbol{F}}}},{{{\boldsymbol{W}}}},{{{\boldsymbol{R}}}}|{{{\boldsymbol{X}}}}} \right) = P({{{\boldsymbol{U}}}},\boldsymbol{E},{{{\boldsymbol{M}}}},{{{\boldsymbol{A}}}},{{{\boldsymbol{T}}}}|{{{\boldsymbol{B}}}},{{{\boldsymbol{V}}}},{{{\boldsymbol{F}}}},{{{\boldsymbol{W}}}},{{{\boldsymbol{R}}}},{{{\boldsymbol{X}}}})\\P({{{\boldsymbol{B}}}},{{{\boldsymbol{V}}}},{{{\boldsymbol{F}}}},{{{\boldsymbol{W}}}},{{{\boldsymbol{R}}}}|{{{\boldsymbol{X}}}})\end{array}$$

We assumed, as a first approximation, that $P\left( {\left. {\left\{ {{{{\mathcal{D}}}}_k} \right\}|\left\{ {{{{\mathcal{D}}}}_k^ \ast } \right\},{{{\boldsymbol{X}}}}} \right)P\left( {\left\{ {{{{\mathcal{D}}}}_k^ \ast } \right\}|{{{\boldsymbol{X}}}}} \right.} \right) = \mathop {\prod}\limits_k P \left( {{{{\mathcal{D}}}}_k|{{{\mathcal{D}}}}_k^ \ast ,{{{\boldsymbol{X}}}}} \right) \cdot \mathop {\prod}\limits_k P ({{{\mathcal{D}}}}_k^ \ast |{{{\boldsymbol{X}}}})$ with *k* as the data source index, and ${{{\mathcal{D}}}}_k$ and ${{{\mathcal{D}}}}_k^ \ast$ as the data source *k* (Supplementary Table 1) and its associated latent variable, respectively. Subsequently, the conditional probability function is given according to equation (1):

1 $$\begin{array}{l}P\left( {{{{\boldsymbol{U}}}},\boldsymbol{E},{{{\boldsymbol{M}}}},{{{\boldsymbol{A}}}},{{{\boldsymbol{T}}}},{{{\boldsymbol{B}}}},{{{\boldsymbol{V}}}},{{{\boldsymbol{F}}}},{{{\boldsymbol{W}}}},{{{\boldsymbol{R}}}}|{{{\boldsymbol{X}}}}} \right) = P\left( {{{{\boldsymbol{U}}}}|{{{\boldsymbol{B}}}},{{{\boldsymbol{X}}}}} \right){{{\boldsymbol{P}}}}\left( {\boldsymbol{E}|{{{\boldsymbol{V}}}},{{{\boldsymbol{X}}}}} \right)\\ {{{\boldsymbol{P}}}}\left( {{{{\boldsymbol{M}}}}{{{\mathrm{|}}}}{{{\boldsymbol{F}}}},{{{\boldsymbol{X}}}}} \right){{{\boldsymbol{P}}}}\left( {{{{\boldsymbol{A}}}}|{{{\boldsymbol{W}}}},{{{\boldsymbol{X}}}}} \right){{{\boldsymbol{P}}}}\left( {{{{\boldsymbol{T}}}}|{{{\boldsymbol{R}}}},{{{\boldsymbol{X}}}}} \right){{{\boldsymbol{P}}}}\left( {{{{\boldsymbol{B}}}},{{{\boldsymbol{V}}}},{{{\boldsymbol{F}}}},{{{\boldsymbol{W}}}},{{{\boldsymbol{R}}}}|{{{\boldsymbol{X}}}}} \right)\end{array}$$

We aimed to maximize the conditional probability function equation (1): namely, we wanted to find the optimal structures and the optimal latent variables that satisfy:

$${{{\hat{\boldsymbol X}}}},\hat {{\frak{D}}}^ \ast = \arg \mathop {{\max }}\limits_{{{{\mathbf{X}}}},{{{\frak{D}}}}^ \ast } P\left( {{\frak{D}},{\frak{D}}^ \ast |{{{\boldsymbol{X}}}}} \right)$$

$${{{\hat{\boldsymbol X}}}},{{{\hat{\boldsymbol B}}}},{{{\hat{\boldsymbol V}}}},{{{\hat{\boldsymbol F}}}},{{{\hat{\boldsymbol W}}}},{{{\hat{\boldsymbol R}}}} = \arg \mathop {{\max }}\limits_{{{{\mathbf{X}}}},{{{\mathbf{V}}}},{{{\mathbf{B}}}},{{{\mathbf{W}}}},{{{\mathbf{F}}}},{{{\mathbf{R}}}}} P\left( {{{{\boldsymbol{U}}}},\boldsymbol{E},{{{\boldsymbol{M}}}},{{{\boldsymbol{A}}}},{{{\boldsymbol{T}}}},{{{\boldsymbol{B}}}},{{{\boldsymbol{V}}}},{{{\boldsymbol{F}}}},{{{\boldsymbol{W}}}},{{{\boldsymbol{R}}}}|{{{\boldsymbol{X}}}}} \right)$$

and thus

$$\begin{array}{l}{{{\hat{\boldsymbol X}}}},{{{\hat{\boldsymbol B}}}},{{{\hat{\boldsymbol V}}}},{{{\hat{\boldsymbol F}}}},{{{\hat{\boldsymbol W}}}},{{{\hat{\boldsymbol R}}}} = \arg \mathop {{\max }}\limits_{{{{\mathbf{X}}}},{{{\boldsymbol{B}}}},{{{\boldsymbol{V}}}},{{{\boldsymbol{F}}}},{{{\boldsymbol{W}}}},{{{\boldsymbol{R}}}}} {{{\boldsymbol{P}}}}\left( {{{{\boldsymbol{U}}}}|{{{\boldsymbol{B}}}},{{{\boldsymbol{X}}}}} \right){{{\boldsymbol{P}}}}\left( {\boldsymbol{E}|{{{\boldsymbol{V}}}},{{{\boldsymbol{X}}}}} \right){{{\boldsymbol{P}}}}\left( {{{{\boldsymbol{M}}}}|{{{\boldsymbol{F}}}},{{{\boldsymbol{X}}}}} \right)\\ {{{\boldsymbol{P}}}}\left( {{{{\boldsymbol{A}}}}|{{{\boldsymbol{W}}}},{{{\boldsymbol{X}}}}} \right){{{\boldsymbol{P}}}}\left( {{{{\boldsymbol{T}}}}|{{{\boldsymbol{R}}}},{{{\boldsymbol{X}}}}} \right){{{\boldsymbol{P}}}}\left( {{{{\boldsymbol{B}}}},{{{\boldsymbol{V}}}},{{{\boldsymbol{F}}}},{{{\boldsymbol{W}}}},{{{\boldsymbol{R}}}}|{{{\boldsymbol{X}}}}} \right)\\ = \arg \mathop {{\max }}\limits_{{{{\mathbf{X}}}},{{{\mathcal{D}}}}^ \ast } \mathop {\prod}\limits_k P \left( {{{{\mathcal{D}}}}_k|{{{\mathcal{D}}}}_k^ \ast ,{{{\boldsymbol{X}}}}} \right) \cdot \mathop {\prod}\limits_k P ({{{\mathcal{D}}}}_k^ \ast |{{{\boldsymbol{X}}}})\end{array}$$

In addition to the five data sources from four experimental methods (Supplementary Table 1), we also included a set of spatial constraints based on additional information about the genome organization. These data were included in the form of general spatial constraints acting on *N* chromatin domains: (i) a nuclear volume confinement restraint that forces all chromatin domains to be inside the nuclear volume, (ii) excluded volume restraints that prevent ‘hard-core’ overlap between any two chromatin domains and (iii) a polymer chain connectivity restraint between chromatin domain neighbors in a chromosome, which guarantees the structural integrity of the chromosomal chains. Additional information about these restraints is available in the Supplementary Information.

In summary, the maximum likelihood problem is formally expressed by equation (2):

2 $${{{\hat{\boldsymbol X}}}},{{{\hat{\boldsymbol B}}}},{{{\hat{\boldsymbol V}}}},{{{\hat{\boldsymbol F}}}},{{{\hat{\boldsymbol W}}}},{{{\hat{\boldsymbol R}}}} = {{{\mathrm{arg}}}}\mathop {{{{{\mathrm{max}}}}}}\limits_{{{{\mathbf{X}}}},{{{\mathbf{V}}}},{{{\mathbf{B}}}},{{{\mathbf{W}}}},{{{\mathbf{F}}}},{{{\mathbf{R}}}}} \left\{ {\log P\left( {{{{\boldsymbol{U}}}},\boldsymbol{E},{{{\boldsymbol{M}}}},{{{\boldsymbol{A}}}},{{{\boldsymbol{T}}}},{{{\boldsymbol{B}}}},{{{\boldsymbol{V}}}},{{{\boldsymbol{F}}}},{{{\boldsymbol{W}}}},{{{\boldsymbol{R}}}}|{{{\boldsymbol{X}}}}} \right)} \right\}$$

$$\mathrm{Subject}\,\mathrm{to}\left\{ {\begin{array}{*{20}{c}} \mathrm{nuclear}\,\mathrm{volume}\,\mathrm{constraint} \\ \mathrm{excluded}\,\mathrm{volume}\,\mathrm{constraint} \\ \mathrm{chain}\,\mathrm{connectivity}\,\mathrm{restraint} \end{array}} \right.$$

### Optimization procedure

We adapted our previously developed iterative optimization procedure to solve this maximum likelihood estimation problem for determining a population of genome structures consistent with all data modalities^36,37,44^. Because there is no closed-form solution to this optimization problem (equation (2)), we developed a variant of the EM method to iteratively optimize local approximations of the log likelihood function^37,44,65^. We use an iterative solver to alternately optimize the latent variables and model parameters in a sequence of so-called modeling (M) and assignment (A) steps until joint convergence was reached.

- Initialization step: an initial model estimate ***X***^0^ is needed to start the first iteration. ***X***^0^ is generated by using random chromatin domain positions that satisfy the three spatial constraints in equation (2), that is, nuclear volume, excluded volume and chain connectivity. Chromatin regions are randomly placed in a bounding sphere proportional to its chromosome territory size and randomly placed within the nucleus followed by a short optimization to eliminate excluded volume steric clashes in the structures.
  Each iteration consists of two steps:
- (1) Assignment step (A-step): given the current estimated population of genome structures ***X***^(*t*)^, which resulted from the previous A/M optimization iteration at step *t*, the optimal latent variables ***B***^*t* + 1^, ***V***^*t* + 1^, ***F***^*t* + 1^, ***W***^*t* + 1^, ***R***^*t* + 1^ are determined by solving the following log likelihood. We use an efficient heuristic strategy to estimate all latent variables (Supplementary Information).
  $$\begin{array}{l}{{{\boldsymbol{B}}}}^{t + 1},{{{\boldsymbol{V}}}}^{t + 1},{{{\boldsymbol{F}}}}^{t + 1},{{{\boldsymbol{W}}}}^{t + 1},{{{\boldsymbol{R}}}}^{t + 1} = \arg max_{{{{\boldsymbol{B}}}},{{{\boldsymbol{V}}}},{{{\boldsymbol{F}}}},{{{\boldsymbol{W}}}},{{{\boldsymbol{R}}}}}\\ \log \left[ \begin{array}{l}P\left( {{{{\boldsymbol{U}}}}|{{{\boldsymbol{B}}}},{{{\boldsymbol{X}}}}^t} \right)P\left( {\boldsymbol{E}|{{{\boldsymbol{V}}}},{{{\boldsymbol{X}}}}^t} \right)P\left( {{{{\boldsymbol{M}}}}|{{{\boldsymbol{F}}}},{{{\boldsymbol{X}}}}^t} \right)P\left( {{{{\boldsymbol{A}}}}|{{{\boldsymbol{W}}}},{{{\boldsymbol{X}}}}^{{{\boldsymbol{t}}}}} \right)\\ P\left( {{{{\boldsymbol{T}}}}|{{{\boldsymbol{R}}}},{{{\boldsymbol{X}}}}^t} \right)P\left( {{{{\boldsymbol{B}}}},{{{\boldsymbol{V}}}},{{{\boldsymbol{F}}}},{{{\boldsymbol{W}}}},{{{\boldsymbol{R}}}}|{{{\boldsymbol{X}}}}^t} \right)\end{array} \right]\end{array}$$
- (2) Modeling step (M-step): given the current latent variables ***B***^*t* + 1^,***V***^*t* + 1^,***F***^*t* + 1^,***W***^*t* + 1^,***R***^*t* + 1^, determined in the A-step, find the genome structure population ***X***^*t* + 1^ that maximizes the log likelihood of all data. A new structure population ***X***^*t* + 1^ is generated in which data assignments in latent variables will be physically present in the structure population ***X***. Optimization is performed in an efficient parallel platform (Supplementary Information).
  $${{{\boldsymbol{X}}}}^{t + 1} = \arg \mathop {{\max }}\limits_{{{\mathbf{x}}}} \log \left[ \begin{array}{l}P\left( {{{{\boldsymbol{U}}}}|{{{\boldsymbol{B}}}}^{t + 1},{{{\boldsymbol{X}}}}} \right)P\left( {\boldsymbol{E}|{{{\boldsymbol{V}}}}^{t + 1},{{{\boldsymbol{X}}}}} \right)P\left( {{{{\boldsymbol{M}}}}|{{{\boldsymbol{F}}}}^{t + 1},{{{\boldsymbol{X}}}}} \right)P\left( {{{{\boldsymbol{A}}}}|{{{\boldsymbol{W}}}}^{t + 1},{{{\boldsymbol{X}}}}} \right)\\ P\left( {{{{\boldsymbol{T}}}}|{{{\boldsymbol{R}}}}^{t + 1},{{{\boldsymbol{X}}}}} \right)P\left( {{{{\boldsymbol{B}}}}^{t + 1},{{{\boldsymbol{V}}}}^{t + 1},{{{\boldsymbol{F}}}}^{t + 1},{{{\boldsymbol{W}}}}^{t + 1},{{{\boldsymbol{R}}}}^{t + 1}|{{{\boldsymbol{X}}}}} \right)\end{array} \right]$$
- Iterate A/M steps until convergence is reached (see Supplementary Information for convergence criteria). This iterative procedure ensures that all data allocations are re-evaluated using the current structure population.

#### Stepwise optimization strategy

We used a stepwise optimization strategy to gradually increase the optimization hardness (Extended Data Fig. 1). An initial model that already fits a portion of the data $\left\{ {{{{\mathcal{D}}}}_k} \right\}$ can guide a more efficient search for the optimum latent variables $\left\{ {{{{\mathcal{D}}}}_k^\prime } \right\}$ than a random structure population. Thus, gradually fitting an increasing number of data points starting from the highest to the lowest data probabilities (that is, domain contacts and domain distances from Hi-C and DamID data), or starting from largest to lowest distance tolerances (for SPRITE and 3D FISH data; Supplementary Information) will effectively guide the search of the optimal solution. In the initial step, we first calculated a structure population ${\boldsymbol{X}}^{{\mathrm{step}}_{1}}$ that integrates only data with the highest probabilities (for Hi-C and DamID data) and performed several rounds of iterative A/M optimizations until convergence is reached. At each following step, we added further data batches with gradually lower probabilities (for Hi-C and lamina DamID), and decreasing tolerances (for SPRITE and FISH data), and performed iterative rounds of A/M optimizations each time until full convergence for all data was reached (that is, all data are reproduced in the models; Extended Data Fig. 2b,c).

How the data are added to the optimization at each step and at what accuracy is controlled by a sequence of nonzero threshold values, and each data type is associated with its own sequence.

- *θ*_1_≥…≥*θ*_final_ indicates the list of gradually decreasing Hi-C probability thresholds, such that the *k*-th step incorporates only those chromatin contacts in ${{{\boldsymbol{A}}}}_{\theta _k}$ with higher probability than *a*_*IJ*_≥*θ*_*k*_, thus ${\boldsymbol{A}}_{\theta_k}=[{\boldsymbol{A}} \ge \uptheta_k]$.
- *λ*_1_≥…≥*λ*_final_ indicates the list of gradually decreasing DamID contact probability thresholds, such that the *k*-th step incorporates those chromatin–NE contacts in $\mathbf{E}_{\lambda _k}$ with higher probabilities than *e*_*I* _≥ *λ*_*k*_, thus ${\mathbf{E}}_{\lambda_{k}} = {\mathbf{E}}\left[{\mathbf{E}} \ge \lambda_{k}\right]$.
- *t*_1_≥…≥*t*_final_ indicates the list of gradually decreasing FISH distance thresholds, such that the *k*-th step in the optimization enforces distance values with a tolerance *t*_*k*_. All FISH distances are incorporated from the first optimization steps on, but their tolerances are gradually reduced with the number of optimization steps.
- *ρ*_1_≤…≤*ρ*_final_ indicates the SPRITE thresholds, such that the *k*-th step enforces clusters with a volume density *ρ*_*k*_. The volume density is related to the cluster radius, as detailed in the (Supplementary Information). All SPRITE clusters are incorporated from the beginning of the optimization, while their effective co-location density is gradually increased with each optimization step (from *ρ*_1_ to *ρ*_final_).

We used a nonzero final bound for each data type (that is, *θ*_final_, *λ*_final_, *t*_final_, *ρ*_final_ > 0) to reduce the chances of including experimental noise in the calculations (that is, data errors are expected to have very low probabilities). To reach convergence, multiple A/M iterations are typically required at a given optimization step, which is defined by a given combination of threshold values (Extended Data Fig. 2b,c). Only if the optimization in a given step is fully converged will the optimization proceed to the next step. All data sources are integrated simultaneously.

The IGM software, as introduced here, automatically performs the sequence of A/M iterations until full convergence is reached and a genome structure population is calculated that recapitulates all the input data (at a given tolerance; Extended Data Fig. 1).

#### Convergence

The optimization progress is monitored by tracking the agreement between model and target distances. As detailed in the Supplementary Information, each energy term introduced in the M-step to model the effect of genomic data is associated with a residual error *η* that monitors whether the corresponding target distance is satisfied or not: *η* > 0.05 indicates a discrepancy between target and model distances larger than 5%, and is considered a violation. A round of A/M iterations (for a given combination of threshold values) is successful when the cumulative fraction of all violations (from all data types) is smaller than 0.01%. Only then does the optimization move to the next step, and optimization thresholds are lowered and more data are added. Extended Data Fig. 2d shows the histogram of residual errors in population HDSF for the different data categories used as input (polymer and volume, Hi-C, lamina DamID, SPRITE and FISH).

#### IGM software

The IGM requires one input file for each data type and a configuration file, which lists all parameters controlling the pipeline, including nuclear shape, genome segmentation/base-pair resolution, nuclear radius, semiaxes and MD time step. The software automatically performs a preliminary statistical analysis of genome structures, including a report of the model quality using the correlation between prediction and experiments, and radial features such as the radial positions of individual chromatin domains in the nucleus.

We refer the interested reader to the documentation for implementation details. Here, we would like to discuss the design guidelines that were cornerstones to the development: flexibility, modularity and user-friendliness.

As for flexibility, the software is able to handle different types of genomes confined to either spherical or ellipsoidal nuclei and can use any combination of ensemble Hi-C, lamin B1 DamID, 3D FISH and SPRITE data points as input. Due to IGM’s modularity, the different parts of the code communicate in such a way that any data type can be added with minimal changes, as long as the data can be cast into an energy term, thus allowing for any data customization that users may require. Parallel computing can be deployed on different schedulers in a straightforward manner. Simulation and optimization setups can be adjusted by editing a text file, which lists all the configuration parameters.

A Python wrapper is available for interfacing the different building blocks and keeping track of the optimization status.

The optimization progress is monitored by a log file that prints all the details, from current iteration violation score to the specific values of thresholds associated with it.

The IGM optimization for a population of 1,000 whole diploid genome structures at 200-kb resolution using ensemble Hi-C, lamin B1 DamID, 3D FISH HIPMap and SPRITE data takes about 10–15 h of computing time, using a controller core with 4 GB of RAM communicating with 250 2-GB-RAM engine processors. The optimized coordinates after each iteration, that is, ***X***^*t*^, are saved in separate files, each ~350 Mb in size. The complete package (and its documentation) is available at https://github.com/alberlab/igm/. In particular, we refer the reader to the README.md file (https://github.com/alberlab/igm/blob/master/README.md/), which also guides the reader through installing and running the platform on a simple demo.

### Simulating structural observables from a population of genome structures

The same notation and variables are used here as in the description above (‘Data source representation’ and ‘Probabilistic formulation of maximum likelihood problem’) and in the Supplementary Information. ${{{\vec{\boldsymbol x}}}}_{is}=(x_{is},y_{is},z_{is})$ denotes the 3D coordinates of locus *i* in structure *s*, *i* and *i*^'^ indicate the two copies of genomic region *I*.

#### Genomic data used as input to IGM

##### Ensemble Hi-C

The Hi-C indicator tensor ***W*** = (*w*_*ijs*_) is computed as

$$w_{ijs} = \left\{ {\begin{array}{*{20}{l}} {1,\,if\,\left\| {{{{\vec{\boldsymbol x}}}}_{is} - {{{\vec{\boldsymbol x}}}}_{js}} \right\|_2 - 2\left( {R_i^{ex} + R_j^{ex}} \right) \le 0} \hfill \\ {0,\,\mathrm{otherwise}} \hfill \end{array}} \right.,$$

$R_i^{ex}$ being the excluded volume locus radius.

The simulated ***A*** = (*a*_*IJ*_) matrix is computed as

$$a_{IJ} = \frac{1}{S}\mathop {\sum}\limits_s {\mathop {\sum}\limits_{\left( {i,i\prime } \right) \in I} {\mathop {\sum}\limits_{\left( {j,j\prime } \right) \in J} {\frac{{w_{ijs}}}{{{{{\mathrm{min}}}}\left( {CN\left( I \right),CN\left( J \right)} \right)}}} } }$$

where *CN*(*I*) indicates the number of homologous copies associated with locus *I*.

##### Lamina DamID

The lamina DamID indicator tensor ***V*** = (*v*_*is*_) is computed as

$$v_{is} = \left\{ {\begin{array}{*{20}{l}} {1,\,if\,\frac{{x_{is}^2}}{{\left[ {a\left( {1 - c_r} \right) - r_0} \right]^2}} + \frac{{y_{is}^2}}{{\left[ {b\left( {1 - c_r} \right) - r_0} \right]^2}} + \frac{{z_{is}^2}}{{\left[ {b\left( {1 - c_r} \right) - r_0} \right]^2}} \ge 1} \hfill \\ {0,\,\mathrm{otherwise}} \hfill \end{array}} \right.$$

where (*a*, *b*, *c*) are the nuclear semiaxes, *r*_0_ is the domain radius in the model, and *c*_*r*_ is the contact range scalar (Supplementary Information). The simulated ***E*** = (*e*_*I*_) matrix is then computed as

$$e_I = \mathop {\sum }\limits_s \frac{1}{S}\mathop {\sum }\limits_{\left( {i,i\prime } \right) \in I} \frac{{v_{is}}}{{CN\left( I \right)}}$$

##### Radial distance distributions (radial 3D HIPMap)

We extract the ordered radial distance distribution of region *I* from the *S* structures in the population. Assuming *I* has two copies, we have the list of distances

$$Z_I = \left\{ {\left\| {{{{\vec{\boldsymbol x}}}}_{is}} \right\|_2,\left\| {{{{\vec{\boldsymbol x}}}}_{i\prime s}} \right\|_2|s = 1, \ldots S} \right\},domain\,I$$

We isolate the *S* maximal and *S* minimal distances, each defining a ‘maximal’ and ‘minimal’ distance distribution. We obtain the two distributions

$$Z_I^{max} = \left\{ {\max \left\{ {\left\| {{{{\vec{\boldsymbol x}}}}_{is}} \right\|_2,\left\| {{{{\vec{\boldsymbol x}}}}_{i\prime s}} \right\|_2} \right\}|s = 1, \ldots S} \right\},$$

$$Z_I^{min} = \left\{ {\min \left\{ {\left\| {{{{\vec{\boldsymbol x}}}}_{is}} \right\|_2,\left\| {{{{\vec{\boldsymbol x}}}}_{i\prime s}} \right\|_2} \right\}|s = 1, \ldots S} \right\}.$$

The collection of *Z* − distance distributions for different chromatin regions are cast into the ***U*** data variables (Supplementary Information) by binning the distances into appropriate ${{{\mathcal{B}}}}_q = \left[ {d_q,d_{q + 1}} \right)$ bins. In particular, if we use those distance distributions as input to an IGM calculation on a population also containing *S* structures (Fig. 5 and Extended Data Fig. 8), we use a straightforward approach whereby each distance in the distribution is the center of a distance bin ${{{\mathcal{B}}}}_q$ (Supplementary Information).

##### Pairwise distance distributions (pairwise 3D HIPMap)

We extract the ordered pairwise distance distribution of genomic pair *I* and *J* from the *S* structures in the population. Assuming *I* and *J* both have two copies, we have the list of distances

$$\begin{array}{l}Z_{IJ} = \left\{ {\left\| {{{{\vec{\boldsymbol x}}}}_{is} - {{{\vec{\boldsymbol x}}}}_{js}} \right\|_2,\left\| {{{{\vec{\boldsymbol x}}}}_{is} - {{{\vec{\boldsymbol x}}}}_{j\prime s}} \right\|_2\left\| {{{{\vec{\boldsymbol x}}}}_{i\prime s} - {{{\vec{\boldsymbol x}}}}_{js}} \right\|_2\left\| {{{{\vec{\boldsymbol x}}}}_{i\prime s} - {{{\vec{\boldsymbol x}}}}_{j\prime s}} \right\|_2|s = 1, \ldots S} \right\},\\{{{\mathrm{Pair}}}}\,I - J\end{array}$$

We isolate the *S* maximal and *S* minimal distances, each defining a ‘maximal’ and ‘minimal’ distance distribution. We obtain the two distributions

$$Z_{IJ}^{max} = \left\{ {\max \left\| {{{{\vec{\boldsymbol x}}}}_{is} - {{{\vec{\boldsymbol x}}}}_{js}} \right\|_2,\left\| {{{{\vec{\boldsymbol x}}}}_{is} - {{{\vec{\boldsymbol x}}}}_{j\prime s}} \right\|_2\left\| {{{{\vec{\boldsymbol x}}}}_{i\prime s} - {{{\vec{\boldsymbol x}}}}_{js}} \right\|_2\left\| {{{{\vec{\boldsymbol x}}}}_{i\prime s} - {{{\vec{\boldsymbol x}}}}_{j\prime s}} \right\|_2|s = 1, \ldots S} \right\}$$

$$Z_{IJ}^{min} = \left\{ {\min \left\| {{{{\vec{\boldsymbol x}}}}_{is} - {{{\vec{\boldsymbol x}}}}_{js}} \right\|_2,\left\| {{{{\vec{\boldsymbol x}}}}_{is} - {{{\vec{\boldsymbol x}}}}_{j\prime s}} \right\|_2\left\| {{{{\vec{\boldsymbol x}}}}_{i\prime s} - {{{\vec{\boldsymbol x}}}}_{js}} \right\|_2\left\| {{{{\vec{\boldsymbol x}}}}_{i\prime s} - {{{\vec{\boldsymbol x}}}}_{j\prime s}} \right\|_2|s = 1, \ldots S} \right\}$$

The collection of *Z* − distance distributions for different pairs of chromatin regions are cast into the ***M*** data variable (Supplementary Information) by binning the distances into appropriate ${{{\mathcal{B}}}}_q = \left[ {d_q,d_{q + 1}} \right)$ bins. In particular, if we use those distance distributions as input to an IGM calculation on a population also containing *S* structures (Fig. 5), we use a straightforward approach whereby each distance in the distribution is the center of a distance bin ${{{\mathcal{B}}}}_q$ (Supplementary Information).

##### Single-cell SPRITE clusters

For a given SPRITE cluster {*I*_1_,…,*I*_*n*_}, we followed the first step of the assignment procedure (Supplementary Information; SPRITE) and determined the optimal diploid representation $\tilde C_n$ for each structure; we computed the SPRITE residual error for all structures: if a structure has no violations, then the cluster is present in that structure, and $t_{I_1, \ldots, I_n} = 1$; If no structure has zero violations, the cluster is not present in the population, that is, $t_{I_1, \ldots, I_n} = 0$ (Fig. 2g).

#### Other structural features

A more detailed description of the following structural features is provided in ref. ^30^.

##### Distance of a locus to the nuclear center and to the lamina

The normalized radial distance of a locus *i* of coordinates (*x*_*is*_, *y*_*is*_, *z*_*is*_) to the nuclear center of an ellipsoidal nucleus (in population structure *s*) is computed as

$$r_{is}^2 = \left\| {{{{\vec{\boldsymbol x}}}}_i} \right\|_2^2 = \left( {\frac{{x_{is}}}{a}} \right)^2 + \left( {\frac{{y_{is}}}{b}} \right)^2 + \left( {\frac{{z_{is}}}{c}} \right)^2$$

that is, locus coordinates are scaled by the corresponding semiaxes. $\left\| {{{{\vec{\boldsymbol x}}}}_i} \right\|_2 = 0$ . 1, indicates that the region is located at the geometric center (nuclear lamina).

The normal distance to an ellipsoidal surface cannot be computed exactly, so we use the radial approximation for the distance to the lamina (NE)

$$d\left( {i,\mathrm{NE}} \right) = \left( {\frac{1}{{\sqrt {\kappa _i(a,b,c)} }} - 1} \right)\left\| {{{{\vec{\boldsymbol x}}}}_i} \right\|_2,\kappa _i(a,b,c) = \frac{{x_i^2}}{{a^2}} + \frac{{y_i^2}}{{b^2}} + \frac{{z_i^2}}{{c^2}}$$

##### Radius of gyration

The radius of gyration of a chromatin segment comprising *C* loci ${{{\mathcal{C}}}} = (i_1,i_2, \ldots ,i_C)$ in genome structure *s* is computed as

$$R_g^2\left[ {{{{\mathcal{C}}}},s} \right] = \frac{1}{C}\mathop {\sum }\limits_{j \in {{{\mathcal{C}}}}} \left( {{{{\vec{\boldsymbol x}}}}_{js} - {{{\vec{\boldsymbol x}}}}_{{{\mathcal{C}}}}^{\mathrm{CM}}} \right)^2$$

where ***x***_*js*_ are the coordinates of the *j*-th locus in the segment, and ${{{\boldsymbol{x}}}}_{{{\mathcal{C}}}}^{\mathrm{CM}}$ is the segment center of mass in structure *s*. The chromosomal radius of gyration is easily computed by replacing a chromatin segment with a whole chromosome.

##### Compartmentalization score

For the HFFc6 cell type, each locus is assigned to either A or B compartments using the ensemble Hi-C and the procedure used in ref. ^8^. For each structure, the compartmentalization score is computed as defined in ref. ^63^:

$$\begin{array}{l}T = N_{AA} + N_{AB} + N_{BB}P\left( A \right) = \frac{{2N_{AA} + N_{AB}}}{T}P\left( B \right) = \frac{{2N_{BB} + N_{AB}}}{T}\\ CompScore = log_2\frac{{2 \cdot P\left( A \right) \cdot P\left( B \right) \cdot T}}{{N_{AB}}}\end{array}$$

where *N*_*AA*_, *N*_*AB*_ and *N*_*BB*_ are the number of A–A, A–B and B–B contacts in the structure respectively. The A/B assignment for HFFc6 structures was downloaded from the 4DN portal^58^ under identifier 4DNFINQZ5JHV.

##### Average radial position

The mean radial position of a locus *I* in an autosome is $\overline {r_I} = \mathop {\sum}\nolimits_{s = 1}^S {\frac{{r_{is} + r_{i\prime s}}}{{2S}}}$, with *i*, *i*′ as the two homologous copies. *S* is the total number of structures in the population^30^.

##### Chromatin decompaction

The local compaction of the chromatin fiber at the location of a given locus is estimated by the radius of gyration for a 1-Mb region centered at the locus (that is, comprising +500 kb upstream and 500 kb downstream of the given locus). To estimate the radius of gyration values along an entire chromosome, we use a sliding-window approach over all chromatin regions in a chromosome, as described in ref. ^30^.

##### Cell-to-cell variability of structural features^30^

Cell-to-cell variability, *δ*, of any structural feature for a chromatin region, *i*, in chromosome *c*, is calculated as

$$\delta _i = log_2\frac{{\sigma _{c,i}}}{{\overline {\sigma _c} }}$$

where *σ*_*c*,*i*_ is the standard deviation of the feature value of region *i* across the population and $\overline {\sigma _c}$ is the mean standard deviation of the feature value calculated from all regions within the same chromosome, *c*. Positive *δ*_*i*_ values (*δ*_*i* _> 0) result from high cell-to-cell variability of the feature (for example, radial position), whereas negative values (*δ*_*I* _< 0) indicate low variability.

##### Interchromosomal interaction probability

For each chromatin region *I*, its interchromosomal interaction probability (ICP) is calculated as

$$\mathrm{ICP}[I] = \frac{{\mathop {\sum}\nolimits_s {n_{I\,\mathrm{inter}}^s} }}{{\mathop {\sum}\nolimits_s {\left( {n_{I,\mathrm{inter}}^s + n_{I,\mathrm{intra}}^s} \right)} }}$$

across the full population, where $n_{\mathrm{intra}}^s$ and $n_{\mathrm{inter}}^s$ are the number of *cis* and *trans* contacts in structure *s*, respectively.

##### Interior chromatin localization

For a given 200-kb region, the interior localization frequency (ILF) is calculated as

$$\mathrm{ILF}[I] = \frac{{n[r_I \le 0.5]}}{S}$$

where *n*[*r*_*I* _≤ 0.5] is the number of structures where either copy of the region *I* has a radial position lower than 0.5, for example, in the nuclear interior.

##### SON TSA-seq

We followed a procedure described in ref. ^30^. We first identified chromatin expected to have high speckle association: we selected 5% of chromatin regions with the lowest average radial positions and generated chromatin interaction networks (CINs)^66^ for the selected group of chromatin regions in each structure of the population. A CIN was calculated for the selected chromatin in each model as follows: Each vertex represents a 200-kb chromatin region. An edge between two vertices *i*, *j* is drawn if the corresponding chromatin regions are in physical contact in the model, if the spatial distance *d*_*ij* _≤ 4*r*_0_. Approximate speckle locations are then identified as the geometric center of the resulting spatial partitions identified by Markov clustering^67^ of the CINs.

To predict TSA-seq signals from our models, we use

$$\mathrm{Sig}_i = \frac{1}{S}\mathop {\sum }\limits_{s = 1}^S \mathop {\sum }\limits_{l = 1}^L e^{ - R_0\left\| {{{{\vec{\boldsymbol x}}}}_{is} - {{{\vec{\boldsymbol x}}}}_{ls}} \right\|_2}$$

where *S* is the number of models, *L* is the number of approximate speckle locations in structure *s*, $\left\| {{{{\vec{\boldsymbol x}}}}_{is} - {{{\vec{\boldsymbol x}}}}_{ls}} \right\|_2$ is the distance between the region *i* and the predicted nuclear body location *l* (in structure *s*), and *R*_0_ = 4 is the estimated decay constant in the TSA-seq experiment^57^. The normalized TSA-seq signal for region *i* then becomes:

$$\mathrm{Predicted}\,\mathrm{TSA}-\mathrm{seq}\,\mathrm{signal}_i = \mathrm{log}\left( {\frac{{\mathrm{sig}_i}}{{\overline {\mathrm{sig}}}}} \right)$$

where $\overline {sig}$ is the mean signal calculated from all regions in the genome. The predicted signal is averaged over copies for regions that have more than one copy in the genome.

##### Lamin B1 TSA-seq

We followed the procedure described in ref. ^30^. For lamin locations, we first identified regions with the highest 15% radial positions in each structure, determined spatial partitions of these regions and used centers of these spatial partitions as approximate locations of lamina-associated domains. Lamina TSA-seq signal was then calculated from these center locations using the decay function described in ‘SON TSA-seq’.

##### Speckle and lamina association frequencies^30^

For a given 200-kb chromatin region *I*, the SAF is calculated as

$$\mathrm{SAF}_I = \frac{{n_{d_i < d_t} + n_{d_{i\prime } < d_t}}}{{2S}}$$

where *S* is the number of structures in the population; $n_{d_i < d_t}$ and $n_{d_{i\prime } < d_t}$ are the number of structures, in which region *i* and its homologous copy *i*′ have a distance to a predicted speckle smaller than the association threshold, *d*_*t*_ (if the chromatin region is from a sex chromosome, there is only one copy and *i*^′^ = *i*). The *d*_*t*_ value is set to 1,000 nm. Distances to the speckles are computed using the predicted speckle partitions via Markov clustering.

For a given 200-kb chromatin region *I*, the LAF is calculated as

$$\mathrm{LAF}_I = \frac{{n_{r_i > 0.85} + n_{r_{i\prime } > 0.85}}}{{2S}}$$

where *S* is the number of structures in the population; *n*_*ri*>0.85_ and *n*_*ri*'>0.85_ are the number of structures, in which region *i* and its homologous copy *i*′ have a radial position larger than 0.85 (if the chromatin region is from a sex chromosome, there is only one copy and *i*′ = *i*). Both for SAF and LAF, we tried different distance thresholds, and the selected thresholds resulted in the best correlations with experimental data. The following experimental threshold distances were used for comparison with the experimental data from Su et al.^17^: SAF of 500 nm and LAF of 750 nm.

##### Median *trans* A/B ratio^17,30^

For each chromatin region *i*, we defined the *trans* neighborhood {*j*} if the center-to-center distances of other regions from other chromosomes to *i* are smaller than 500 nm, which can be expressed as a set; $Ne_i^t = \{ j:\mathrm{chrom}_i \ne \mathrm{chrom}_j,d_{ij} < 500\,\mathrm{nm}\}$. The *trans* A/B ratio is then calculated as

$$trans\,\mathrm{AB}\,\mathrm{ratio}_i = \frac{{n_A^t}}{{n_B^t}}$$

where $n_A^t$ and $n_B^t$ are the number of *trans* A and B regions in the set *Ne*_*i*_ for haploid region *i*. The median of the *trans* A/B ratios for a region is then calculated from all the *trans* A/B ratios of the homologous copies of the region observed in all the structures of the population. The values are then rescaled to have values between 0 and 1.

### Comparison of simulated structures with imaged single cells

#### Preprocessing of the DNA-MERFISH dataset^17^

We collected both homologous chromosome copies from each of the 3,029 single cells that contained at least 80% assigned imaged loci and where all chromosomes are imaged. There were 935 loci for 3,029 different single cells for the high-resolution chromosome 2 dataset and 1,041 loci for 4,555 different single cells for the low-resolution whole-genome-imaged dataset. If a locus is unidentified in an image, we used linear interpolation to approximate its coordinates within the image. For low-resolution chromosome 6 data, we filtered out those structures containing at least 75% of assigned loci.

#### Preprocessing of the IGS dataset^68^

We collected both copies from each single cell for the target chromosomes. Because the number of imaged loci varies per chromosome, we considered only chromosome structures with a coverage of at least ten genomic regions in a single cell to allow meaningful comparisons. At the end of the pipeline, there were 82 imaged single cells for chromosome 2 and 52 for chromosome 6.

#### Calculation and comparison of distance matrices

Chromosome structures were extracted from the images and imaged loci mapped to genomic bins at 200-k base-pair resolution. To compare structures from models and microscopy images, we only considered loci in the models that had been imaged in experiments.

We computed the distance matrix for each structure *s* as

$$D_s = \left( {d_{ijs}} \right) \in {\Bbb R}^{n \times n},d_{ijs} = \left\| {{{{\vec{\boldsymbol x}}}}_{is} - {{{\vec{\boldsymbol x}}}}_{js}} \right\|_2,$$

where *n* is the number of loci in the chromosome at 200-kb resolution and coordinates are from either one of the simulated or the imaged chromosomal structures.

The matching score between any two structures is the Pearson correlation coefficient between the corresponding minimum–maximum normalized (flattened) distance matrices. To search for matching structures, we iterated over all possible structure pairs, and identified for each structure in one set its best match in the other by selecting the one with the largest correlation score.

### Data analysis

#### Correlations

Unless otherwise specified, Pearson correlation was used to compare a given quantity across different populations. All Pearson correlation values are associated with a *P* value < 10^−8^ and we indicated that with ~0. The chromosomal stratum-adjusted correlation coefficients in Supplementary Table 3 were computed following the procedure detailed by Yang et al.^60^, using a smoothing parameter *h* = 0 and an upper-bound resolution of 50 Mb.

#### Goodness-of-fit test

We performed a chi-squared goodness-of-fit test on all four input data types (that is, Hi-C, lamin B1 DamID, 3D HIPMap FISH and single-cell SPRITE) of the HDSF population of structures. The test null hypothesis is that both the input data (from the experiment) and the output data (simulated from the structure population) are drawn from the same underlying distribution. We used a standard confidence value *α* = 0.05 for assessing the test results. For Hi-C and lamin B1 DamID data, the modeled and experimental cumulative distributions of probability of locus–locus contacts of a locus with another or the NE were compared, respectively. For 3D HIPMap data, the modeled and experimental cumulative pairwise distance distributions were compared. As for single-cell SPRITE data, we assigned a value of 1 or 0 to any of the 6,617 SPRITE clusters from the experiment that were or were not present in any of the structures of the population, by quantifying the SPRITE residual errors (Methods and Supplementary Information). The resulting distribution of binary values was then compared with the experimental distribution, which only contained values of 1. Large *P* values associated with the test statistics indicate that the initial null hypothesis can be rejected with great confidence; thus, it is reasonable to assume that input and output come from the same distribution (Extended Data Fig. 3).

#### Error bars

Error bars in Figs. 4, 5c,d and 6c and Extended Data Fig. 8b,c were computed by generating three independent population replicates for each modeling setup. Each replicate started from different random starting conditions. Any two replicates differ in the initial coordinate initialization ${{{\boldsymbol{X}}}}_i^0 \ne {{{\boldsymbol{X}}}}_j^0$, and undergo the same optimization procedure. Different random seeds were used each time to generate initial random chromosome positions within the nuclear volume. The average and standard deviation of the statistics from the three replicates are plotted in the figures.

#### Cross-Wasserstein distance

Let *Q* and *P* denote the cumulative probability distributions of distributions *q* and *p* of variable *y*, then the Wasserstein distance (WD)

$$\mathrm{WD}\left( {p,q} \right) = {\int} {|P - Q|dy}$$

is customarily used to estimate the amount of work required to transform one distribution into the other; ‘work’ measured as the amount of distribution weight to be moved, multiplied by the distance it has to be moved. We used the ordinary Wasserstein distance to compare two distributions within the same population.

When comparing probability distributions between two different genome populations or between one population and a set of experimental data, we used the notion of cross- (‘all versus all’) Wasserstein distance: we computed the set of all Wasserstein distance values for applicable distribution pairs within the same populations (cross-WD) and then computed a simple correlation between the two sets (score). Let us assume we want to compare the set of distance distributions of *n* pairs *C* = {(*i*_1_,*j*_1_),⋯,(*i*_*n*_,*j*_*n*_)} between population 1 and population 2 (either one could be an experimental distribution), then we will compute

$$\begin{array}{l}\mathrm{WD}_{score} = \mathrm{Pearson}\left[ {\mathrm{cross}\,\mathrm{WD}_1,\,\mathrm{cross}\,\mathrm{WD}_2} \right]\\ = \mathrm{Pearson}\left[ {\left\{ {\mathrm{WD}_1\left( {p_{ij}} \right)} \right\}_{\left( {i,j} \right) \in C},\left\{ {\mathrm{WD}_2\left( {p_{mn}} \right)} \right\}_{\left( {m,n} \right) \in C}} \right]\end{array}$$

which is the correlation between two sets of *n*(*n* − 1)/2 Wasserstein distance values. For a given haploid pair *I*−*J*, the four diploid pair distributions were concatenated, $p_{IJ} = p_{ij} \cup p_{ij\prime } \cup p_{i\prime j} \cup p_{i\prime j\prime }$. We use cross-Wasserstein distance to compare distance distributions in Fig. 2e, to compare radial, *cis* and *trans* pairwise distance distributions, and chromosomal radius of gyration in Figs. 5c and 6c and Extended Data Fig. 8b.

#### Data analysis

The codes used in our work are based on standard, publicly available software packages. Pre- and post-processing data and the generation of figures were performed using the Anaconda (v4.10) packages Matplotlib v3.4, Scikit Learn v1.0, Scipy v1.5 and NetworkX v2.3. Figures were then assembled using Adobe Illustrator. Chimera (v1.13)^69^ was used for visualization of the 3D structures generated.

### Reporting summary

Further information on research design is available in the Nature Research Reporting Summary linked to this article.

## Online content

Any methods, additional references, Nature Research reporting summaries, source data, extended data, supplementary information, acknowledgements, peer review information; details of author contributions and competing interests; and statements of data and code availability are available at 10.1038/s41592-022-01527-x.

## Supplementary information

Supplementary Information Supplementary Discussion and Supplementary Tables 1–3

Reporting Summary

## Data Availability

The following datasets were used to generate or validate the structures: ensemble Hi-C (4DN portal; accession code 4DNES2R6PUEK), lamin B1 DamID (4DN portal; accession code 4DNESXZ4FW4T), 3D HIPMap FISH (4DN portal; https://data.4dnucleome.org/publications/80007b23-7748-4492-9e49-c38400acbe60), single-cell SPRITE (4DN portal identifier: 4DNESJYGTI8S, private), SON TSA-seq (4DN portal; 4DNES85R9TIB), transcription data (ENCODE; accession code ENCSR735JKB). Super-resolution single-cell imaging data are available at the referenced papers. The pre-processed experimental inputs of different data sources (Hi-C, lamin B1 DamID, 3D HIPMap FISH and single-cell SPRITE) for the HFF cell line and the simulated HDSF population are available at 10.5281/zenodo.6540731. Other data (including configuration files and synthetic data input files) are available upon request. The configuration files and pre-processed data input files are sufficient to reproduce the structure populations with the IGM software.

## Code availability

The IGM platform is available at www.github.com/alberlab/igm/. This includes, but is not limited to, the source code, a README file detailing code installation and execution, accompanying documentation, and a demo that uses a reduced data input for users to familiarize with the input, expected outputs and execution steps.
