# Supplementary material for: Integrative genome modeling platform reveals essentiality of rare contact events in 3D genome organizations
Source: Nat Methods. 2022 Jul 11;19(8):938–49. doi: 10.1038/s41592-022-01527-x (PMC9349046; doi:10.1038/s41592-022-01527-x)
Supplement: Supplementary file 1 — Supplementary Discussion and Supplementary Tables 1–3 [file 41592_2022_1527_MOESM1_ESM.pdf]

nature portfolio

https://doi.org/10.1038/s41592-022-01527-x

Supplementary information

Integrative genome modeling platform reveals essentiality of rare contact events in 3D genome organizations

In the format provided by the authors and unedited

Supplementary Information:

Integrative Genome Modeling Platform reveals essentiality of rare contact events in 3D genome organizations

Lorenzo Boninsegna[1,2], Asli Yildirim[1,2], Guido Polles[1,2,3], Yuxiang Zhan[1,2,3], Sofia A. Quinodoz[4], Elizabeth H. Finn[5], Mitchell Guttman[4], Xianghong Jasmine Zhou[6], Frank Alber[1,2,3*]

[1]Institute of Quantitative and Computational Biosciences (QCBio), University of California Los Angeles, Los Angeles, CA 90095, USA

[2]Department of Microbiology, Immunology, and Molecular Genetics, University of California Los Angeles, 520 Boyer Hall, Los Angeles, CA 90095, USA

[3]Department of Quantitative and Computational Biology, University of Southern California, Los Angeles, CA 90089, USA

[4]Division of Biology and Biological Engineering, California Institute of Technology, Pasadena, CA 91125, USA

[5]National Cancer Institute, NIH, Bethesda, MD 20892, USA

[6]Department of Pathology, David Geffen School of Medicine, University of California Los Angeles, 10833 Le Conte Ave, Los Angeles, CA 90095, USA

* To whom correspondence should be addressed.

Tel: +1 310-267-0363

Email: falber@g.ucla.edu

Table of contents:

1. Probabilistic formulation of the maximum likelihood problem
2. A/M optimization: Assignment Step
    a. 3D radial HIPMap FISH data
    - Lamina DamID
    - 3D pairwise HIPMap FISH data
    - Ensemble Hi-C
    - SPRITE
3. A/M optimization: Modeling Step
    a. 3D radial HIPMap FISH data
    - Lamina DamID
    - 3D pairwise HIPMap FISH data
    - Ensemble Hi-C
    - SPRITE
    - Additional restraints: polymer and volume
4. Synthetic data simulations
5. Iterative contact refinement step
6. HFFc6 experimental data pre-processing:
    - Ensemble Hi-C
    - Lamina DamID
    - DNA SPRITE
    - 3D HIPMap FISH
    - SON TSA-seq
    - Transcription Data
7. Chromosomal Hi-C correlations

1. Probabilistic formulation of the maximum likelihood problem

We introduced a set of data variables $\{\mathcal{D}_k | k = 1, \dots 5\} = \{\boldsymbol{U}, \boldsymbol{E}, \boldsymbol{M}, \boldsymbol{A}, \boldsymbol{T}\}$ and a set of indicator tensors $\{\mathcal{D}_k^* | k = 1, \dots, 5\} = \{\boldsymbol{B}, \boldsymbol{V}, \boldsymbol{F}, \boldsymbol{W}, \boldsymbol{R}\}$ as latent variables that augment missing information in data variables to distinguish homologues chromatin domain copies and in single cells.

Data source	Data interpretation	Data type	Data variable: $\mathfrak{D}$	Latent variable: $\mathfrak{D}^*$
3D HIPMap radial FISH	Univariate radial distance distributions	univariate	$\mathcal{D}_1$: $\boldsymbol{U} = (u_{Iq})_{H \times Q}$	$\mathcal{D}_1^*$: $\boldsymbol{B} = (b_{iqs})_{N \times Q \times S}$
Lamina DamID	Lamina-chromatin contacts	univariate	$\mathcal{D}_2$: $E = (e_I)_H$	$\mathcal{D}_2^*$: $\boldsymbol{V} = (v_{is})_{N \times S}$
3D HIPMap pairwise FISH	Bivariate distance distributions	bivariate	$\mathcal{D}_3$: $\boldsymbol{M} = (m_{IJq})_{H \times H \times Q}$	$\mathcal{D}_3^*$: $\boldsymbol{F} = (f_{ijqs})_{N \times N \times Q \times S}$
Ensemble Hi-C	Pairwise chromatin contacts	bivariate	$\mathcal{D}_4$: $\boldsymbol{A} = (a_{IJ})_{H \times H}$	$\mathcal{D}_4^*$: $\boldsymbol{W} = (w_{ijs})_{N \times N \times S}$
Single cell SPRITE	Multi-way chromatin contacts	multivariate	$\mathcal{D}_5$: $\boldsymbol{T^n} = (t_{I_1,\dots I_n})_{H^n}$	$\mathcal{D}_5^*$: $\boldsymbol{R^n} = (r_{i_1,\dots,i_n s})_{N^n \times S}$

Table S1 List of the five data sources used in genome population modeling (Hi-C, lamina DamID, 3D HIPMap pairwise and radial FISH, and single cell SPRITE), see also **Fig. 1**. The k-th data source is associated with its interpretation, its data variable $\mathcal{D}_k$ and its latent variable $\mathcal{D}_k^*$. H indicates the number of domains, N $(> H)$ indicates the total number of genomic loci (two homologous copies are distinguished), Q is the number of distance bins and S is the total number of structures in the population.

Given $\{\mathcal{D}_k\}$, we aim to estimate the structure population model $\boldsymbol{X}$ such that the likelihood $P(\{\mathcal{D}_k\}, \{\mathcal{D}_k^*\} | \boldsymbol{X}) = P(\boldsymbol{U}, \boldsymbol{E}, \boldsymbol{M}, \boldsymbol{A}, \boldsymbol{T}, \boldsymbol{B}, \boldsymbol{V}, \boldsymbol{F}, \boldsymbol{W}, \boldsymbol{R} | \boldsymbol{X})$ is maximized. The statistical dependence

relationship between data sources and latent variables in an optimized structure population is $\boldsymbol{X} \to \mathcal{D}_k^* \to \mathcal{D}_k$, $\forall k$, because $\{\mathcal{D}_k^*\}$ is a detailed expansion of $\{\mathcal{D}_k\}$ at the diploid and single-structure representation of the data and $\boldsymbol{X}$ is the structure population consistent with to $\{\mathcal{D}_k^*\}$. Therefore, the likelihood $P(\{\mathcal{D}_k\},\{\mathcal{D}_k^*\}|\boldsymbol{X})$ can be expanded to $P(\{\mathcal{D}_k\}|\{\mathcal{D}_k^*\},\boldsymbol{X})P(\{\mathcal{D}_k^*\}|\boldsymbol{X})$ and therefore

$$P(\boldsymbol{U},E,\boldsymbol{M},\boldsymbol{A},\boldsymbol{T},\boldsymbol{B},\boldsymbol{V},\boldsymbol{F},\boldsymbol{W},\boldsymbol{R}|\boldsymbol{X}) = P(\boldsymbol{U},E,\boldsymbol{M},\boldsymbol{A},\boldsymbol{T}|\boldsymbol{B},\boldsymbol{V},\boldsymbol{F},\boldsymbol{W},\boldsymbol{R},\boldsymbol{X})P(\boldsymbol{B},\boldsymbol{V},\boldsymbol{F},\boldsymbol{W},\boldsymbol{R}|\boldsymbol{X})$$

We assume, as a first approximation, that $P(\{\mathcal{D}_k\}|\{\mathcal{D}_k^*\},\boldsymbol{X})P(\{\mathcal{D}_k^*\}|\boldsymbol{X}) = \prod_k P(\mathcal{D}_k|\mathcal{D}_k^*,\boldsymbol{X}) \cdot \prod_k P(\mathcal{D}_k^*|\boldsymbol{X})$ with k as the data source index, and $\mathcal{D}_k$ and $\mathcal{D}_k^*$ as the data source k (Supplementary **Table S1**) and its associated latent variable respectively. Subsequently the conditional probability function is expressed as:

$$\begin{aligned} &P(\boldsymbol{U},E,\boldsymbol{M},\boldsymbol{A},\boldsymbol{T},\boldsymbol{B},\boldsymbol{V},\boldsymbol{F},\boldsymbol{W},\boldsymbol{R}|\boldsymbol{X}) \\ &= P(\boldsymbol{U}|\boldsymbol{B},\boldsymbol{X})P(E|\boldsymbol{V},\boldsymbol{X})\boldsymbol{P}(\boldsymbol{M}|\boldsymbol{F},\boldsymbol{X})\boldsymbol{P}(\boldsymbol{A}|\boldsymbol{W},\boldsymbol{X})\boldsymbol{P}(\boldsymbol{T}|\boldsymbol{R},\boldsymbol{X})\boldsymbol{P}(\boldsymbol{B},\boldsymbol{V},\boldsymbol{F},\boldsymbol{W},\boldsymbol{R}|\boldsymbol{X}) \qquad (\mathbf{Eq.\,S1}) \end{aligned}$$

We aim to maximize the conditional probability function **Equation S1**: namely, we want to find the optimal structures and the optimal latent variables which satisfy:

$$\widehat{\boldsymbol{X}},\widehat{\mathfrak{D}^*} = \arg\max_{\mathbf{X},\mathcal{D}^*} P(\mathfrak{D},\mathfrak{D}^*|\boldsymbol{X})$$

$$\widehat{\boldsymbol{X}},\widehat{\boldsymbol{B}},\widehat{\boldsymbol{V}},\widehat{\boldsymbol{F}},\widehat{\boldsymbol{W}},\widehat{\boldsymbol{R}} = \arg\max_{\mathbf{X},\mathbf{V},\mathbf{B},\mathbf{W},\mathbf{F},\mathbf{R}} P(\boldsymbol{U},E,\boldsymbol{M},\boldsymbol{A},\boldsymbol{T},\boldsymbol{B},\boldsymbol{V},\boldsymbol{F},\boldsymbol{W},\boldsymbol{R}|\boldsymbol{X})$$

and thus

$$\begin{aligned} &\widehat{\boldsymbol{X}},\widehat{\boldsymbol{B}},\widehat{\boldsymbol{V}},\widehat{\boldsymbol{F}},\widehat{\boldsymbol{W}},\widehat{\boldsymbol{R}} \\ &= \arg\max_{\mathbf{X},\boldsymbol{B},\boldsymbol{V},\boldsymbol{F},\boldsymbol{W},\boldsymbol{R}} \boldsymbol{P}(\boldsymbol{U}|\boldsymbol{B},\boldsymbol{X})\boldsymbol{P}(E|\boldsymbol{V},\boldsymbol{X})\boldsymbol{P}(\boldsymbol{M}|\boldsymbol{F},\boldsymbol{X})\boldsymbol{P}(\boldsymbol{A}|\boldsymbol{W},\boldsymbol{X})\boldsymbol{P}(\boldsymbol{T}|\boldsymbol{R},\boldsymbol{X})\boldsymbol{P}(\boldsymbol{B},\boldsymbol{V},\boldsymbol{F},\boldsymbol{W},\boldsymbol{R}|\boldsymbol{X}) \\ &= \arg\max_{\mathbf{X},\mathcal{D}^*} \prod_k P(\mathcal{D}_k|\mathcal{D}_k^*,\boldsymbol{X}) \cdot \prod_k P(\mathcal{D}_k^*|\boldsymbol{X}) \end{aligned}$$

In the following section, we define the mathematical formulation of $P(\mathcal{D}_k^*|\boldsymbol{X})$ and $P(\mathcal{D}_k|\mathcal{D}_k^*,\boldsymbol{X})$ for all K data sources.

We will use the same notation from *Methods* and **Table S1**. $\vec{\boldsymbol{x}}_{is} = (x_{is},y_{is},z_{is}) \in \mathbb{R}^3$ is the coordinate of locus i in structure s of the population, and we use $\|\ldots\|_2$ to denote the Euclidean norm of a vector.

Note that capital letter indices, such as I and J, relate to genomic regions without distinguishing between two homologous copies, while lower letter indices i, i' and j, j' distinguish between the two copies.

Let us also assume that $q_{d=\bar{d}}$ is an integer labeling the bin into which distance $\bar{d}$ falls, e.g. $\bar{d} \in \mathcal{B}_{q_{d=\bar{d}}}$.

<u>Distributions of radial positions of genomic regions from 3D HIPMap FISH experiments</u>

<u>Expansion of $P(\boldsymbol{B}|\boldsymbol{X})$</u>

The binary valued latent tensor $\boldsymbol{B} = (b_{iqs})_{N\times Q\times S}$ indicates whether the i-th chromatin domain in structure s has a radial position in the range defined by bin $\mathcal{B}_q = [d_q, d_{q+1})$ ($b_{iqs} = 1$) or not ($b_{iqs} = 0$). Because data point from radial FISH data are independent from one another we can expand $P(\boldsymbol{B}|\boldsymbol{X})$ as $P(\boldsymbol{B}|\boldsymbol{X}) = \prod_{i,q,s} p(b_{iqs}|\vec{\boldsymbol{x}}_{is})$, with $\vec{\boldsymbol{x}}_{is}$ as the coordinate vector of chromatin domain i in structure s. Because b_{iqs} takes only binary values, the probabilities $p(b_{iqs}|\vec{\boldsymbol{x}}_{is})$ are Bernoulli distributed:

$$p(b_{iqs}|\vec{\boldsymbol{x}}_{is}) = p(b_{iqs} = 1|\vec{\boldsymbol{x}}_{is})^{b_{iqs}}\left[1 - p(b_{iqs} = 1|\vec{\boldsymbol{x}}_{is})\right]^{1-b_{iqs}}$$

We model the radial position of a chromatin domain $p(b_{iqs} = 1|\vec{\boldsymbol{x}}_{is})$ by a mixture of a constant function and two one-sided truncated Gaussian functions with small variance, which ensures a smooth differentiable representation of the probability outside the target range (see also Li *et al.*[1,2]).

$$p(b_{iqs} = 1|\vec{\boldsymbol{x}}_{is}) = \begin{cases} \exp\left[-\dfrac{\left(\|\vec{\boldsymbol{x}}_{is}\|_2 - d_q\right)^2}{2\sigma_b^2}\right], & if\ \|\vec{\boldsymbol{x}}_{is}\|_2 < d_q \\ 1, & if\ d_q \le \|\vec{\boldsymbol{x}}_{is}\|_2 < d_{q+1} \\ \exp\left[-\dfrac{\left(\|\vec{\boldsymbol{x}}_{is}\|_2 - d_{q+1}\right)^2}{2\sigma_b^2}\right], & if\ \|\vec{\boldsymbol{x}}_{is}\|_2 \ge d_{q+1} \end{cases}$$

Note that, without losing generalization, we use the origin (0,0,0) as the nuclear center, thus the Euclidian norm of the coordinate vector of chromatin $\|\vec{\boldsymbol{x}}_{is}\|_2$, is equivalent to its distance from the nuclear center. d_q and d_{q+1} are the lower and upper bound of radial distances that define the radial distance range in bin $\mathcal{B}_q$.

Expansion of $P(\boldsymbol{U}|\boldsymbol{B},\boldsymbol{X})$

Given $\boldsymbol{U} = (u_{Iq})_{H\times Q}$ as the probability of radial positions derived from 3D FISH HIPMap, $P(\boldsymbol{U}|\boldsymbol{B})$ can be expanded as $P(\boldsymbol{U}|\boldsymbol{B},\boldsymbol{X}) = \prod_{I,q} P(u_{Iq}|u'_{Iq})$, where u'_{Iq} is the probability of genomic region I to have a radial position q in the range defined by bin $\mathcal{B}_q = [d_q, d_{q+1})$.

u'_{Iq} is calculated from $\boldsymbol{B}$ as

$$u'_{Iq} = \frac{1}{CN(I)} \cdot \frac{1}{S} \sum_{s=1}^{S} \bar{b}_{Iqs}$$

where $\overline{\boldsymbol{B}} = (\bar{b}_{Iqs})_{H\times S\times Q}$ is the "projected radial position tensor", which is derived from $\boldsymbol{B}$ by projecting its representation (with homologous copies) to its counterpart without distinguishing between homologous copies. $CN(I)$ indicates the number of homologous chromatin domain copies for genomic region I, i.e., 2 for autosomal chromosomes and 1 otherwise. For instance, the projected tensor $\overline{\boldsymbol{B}} = (\bar{b}_{Iqs})_{H\times S}$ takes values $\bar{b}_{Iqs} \in \{0,1,2\}$ for genomic regions located in autosomes and $\bar{b}_{Iqs} \in \{0,1\}$ otherwise. An element $\bar{b}_{Iqs} = 1$ indicates that one copy of domain I has radial distance in bin $\mathcal{B}_q = [d_q, d_{q+1})$ in structure s, while $\bar{b}_{Iqs} = 2$ indicates that both diploid copies of domains I do.

We then model each u_{Iq} as $u_{Iq} = u'_{Iq} + \varepsilon_I$, where ε_I are independent and identical normally distributed random variables with mean zero and $\varepsilon_I =\propto \mathcal{N}(0, \sigma_I^2)$. (ε_I is effectively set to 0).

Probability of chromatin contacts with the lamina from Lamin B1 DamID data (univariate data)

Expansion of $P(\boldsymbol{V}|\boldsymbol{X})$

We introduced the binary valued latent tensor $\boldsymbol{V} = (v_{is})_{N\times S}$, which indicates whether the i-th chromatin domain is in contact with nuclear lamina in the s-th structure of the population ($v_{is} = 1$) or not ($v_{is} = 0$). Again:

$$P(\boldsymbol{V}|\boldsymbol{X}) = \prod_{i,s} p(v_{is}|\vec{\boldsymbol{x}}_{is})$$

$$p(v_{is}|\vec{\boldsymbol{x}}_{is}) = p(v_{is} = 1|\vec{\boldsymbol{x}}_{is})^{v_{is}}[1 - p(v_{is} = 1|\vec{\boldsymbol{x}}_{is})]^{1-v_{is}}$$

and

$$p(v_{is}=1|\vec{\boldsymbol{x}}_{is}) = \begin{cases} \exp\left[-\frac{\left(\left(\frac{1}{\sqrt{\kappa_{is}(r_0,c_r)}}-1\right)\|\vec{\boldsymbol{x}}_{is}\|_2\right)^2}{2\sigma_v^2}\right], & if\ \kappa_{is} \le 1 \\ 1, & otherwise \end{cases}$$

The numerator of the exponential approximates the distance of a genomic region to the nuclear envelope. $\kappa_{is}(r_0,c_r) = \frac{x_{is}^2}{[a(1-c_r)-r_0]^2} + \frac{y_{is}^2}{[b(1-c_r)-r_0]^2} + \frac{z_{is}^2}{[b(1-c_r)-r_0]^2}$, (a,b,c) are the nuclear ellipsoid semi axes and $c_r > 0$ is a threshold defining the interaction proximity of a genomic region with the nuclear lamina (see also "Simulate structural observables from a population of genome structures" and *Supporting Information* for more details). If the cell shape is spherical, such as in GM12878 cells, the calculation of the normal distance to the nuclear envelope can be performed exactly, and we would recover the probability distribution from Li *et al.*[2]

<u>Expansion of $P(E|\boldsymbol{V},\boldsymbol{X})$</u>

$P(E|\boldsymbol{V})$ can be expanded as $P(E|\boldsymbol{V},\boldsymbol{X}) = \prod_I P(e_I|e_I')$ where e_I' is the lamina-chromatin contact probability of the genomic region I computed from $\boldsymbol{V}$ as

$$e_I' = \frac{1}{CN(I)} \cdot \frac{1}{S}\sum_{s=1}^{S} \bar{v}_{Is}$$

where $\overline{\boldsymbol{V}} = (\bar{v}_{Is})_{H\times S}$ is the "*projected* lamina-chromatin contact tensor", which is derived from the latent lamina contact tensor $\boldsymbol{V}$ by projecting its representation (with homologues copies) to its counterpart without distinguishing between homologous copies. $CN(I)$ indicates the number of homologous chromatin domain copies for genomic region I, i.e., 2 for autosomal chromosomes and 1 otherwise. For instance, the projected tensor $\overline{\boldsymbol{V}} = (\bar{v}_{Is})_{H\times S}$ takes values $\bar{v}_{Is} \in \{0,1,2\}$ for genomic regions located in autosomes and $\bar{v}_{Is} \in \{0,1\}$ otherwise. An element $\bar{v}_{Is} = 1$ indicates that one copy of domain I is in contact with the lamina at the nuclear envelope in structure s, while $\bar{v}_{Is} = 2$ indicates that both diploid copies of domains I are in contact with the lamina in structure s (see also Li et al, [2]).

We then model each e_I as $e_I = e_I' + \varepsilon_I$, where ε_I are independent and identical normally distributed random variables with mean zero and $\varepsilon_I =\propto \mathcal{N}(0, \sigma_I^2)$. (ε_I is effectively set to 0).

Pairwise Distance distributions from 3D FISH HIPMap experiments (bivariate data)

Expansion of $P(\boldsymbol{F}|\boldsymbol{X})$

Pairwise distance distributions are cast in the form $\boldsymbol{M} = (m_{IJq})_{H\times H\times Q}$, with m_{IJq} being the probability that genomic loci I and J have a distance in the range defined by bin $\mathcal{B}_q = [d_q, d_{q+1})$. For each pair of genomic regions (I,J), the all-distance normalization $\sum_q m_{IJq} = 1$ holds. The corresponding latent variable is the indicator tensor $\boldsymbol{F} = (f_{ijqs})_{N\times N\times Q\times S}$ and $P(\boldsymbol{F}|\boldsymbol{X})$ is approximated as:

$$P(\boldsymbol{F}|\boldsymbol{X}) = \prod_{i,j,q,s} p\left(f_{ijqs}|\vec{\boldsymbol{x}}_{is}, \vec{\boldsymbol{x}}_{js}\right)$$

Which now depends on two different genomic loci i and j. The probability associated with entries of tensor $\boldsymbol{F}$ is then defined as:

$$p\left(f_{ijqs}|\vec{\boldsymbol{x}}_{is}, \vec{\boldsymbol{x}}_{js}\right) = p\left(f_{ijqs} = 1|\vec{\boldsymbol{x}}_{is}, \vec{\boldsymbol{x}}_{js}\right)^{f_{ijqs}}\left[1 - p\left(f_{ijqs} = 1|\vec{\boldsymbol{x}}_{is}, \vec{\boldsymbol{x}}_{js}\right)\right]^{1-f_{ijqs}}$$

$$p\left(f_{ijqs} = 1|\vec{\boldsymbol{x}}_{is}, \vec{\boldsymbol{x}}_{js}\right) = \begin{cases} \exp\left[-\dfrac{\left(\left\|\vec{\boldsymbol{x}}_{is} - \vec{\boldsymbol{x}}_{js}\right\|_2 - d_q\right)^2}{2\sigma_f^2}\right], & if\ \left\|\vec{\boldsymbol{x}}_{is} - \vec{\boldsymbol{x}}_{js}\right\|_2 < d_q \\ 1, & if\ d_q \le \left\|\vec{\boldsymbol{x}}_{is} - \vec{\boldsymbol{x}}_{js}\right\|_2 < d_{q+1} \\ \exp\left[-\dfrac{\left(\left\|\vec{\boldsymbol{x}}_{is} - \vec{\boldsymbol{x}}_{js}\right\|_2 - d_{q+1}\right)^2}{2\sigma_f^2}\right], & if\ \left\|\vec{\boldsymbol{x}}_{is} - \vec{\boldsymbol{x}}_{js}\right\|_2 \ge d_{q+1} \end{cases}$$

Expansion of $P(\boldsymbol{M}|\boldsymbol{F}, \boldsymbol{X})$

Given $\boldsymbol{M} = \left(m_{IJq}\right)_{H\times H\times Q}$ as the probability derived from 3D FISH HIPMap distance distributions, $P(\boldsymbol{M}|\boldsymbol{F})$ can be expanded as $P(\boldsymbol{M}|\boldsymbol{F}, \boldsymbol{X}) = \prod_{I,J,q} P\left(m_{IJq}|m_{IJq}'\right)$, where m_{IJq}' is the probability of the genomic regions I and J to have a distance q in the range defined by bin $\mathcal{B}_q = [d_q, d_{q+1})$.

m_{IJq}' is calculated from $\boldsymbol{F}$ as

$$m_{IJq}' = \frac{1}{\min\left(CN(I), CN(J)\right)} \cdot \frac{1}{S}\sum_{s=1}^{S} \bar{f}_{IJqs}$$

where $\bar{\boldsymbol{F}} = \left(\bar{f}_{IJqs}\right)_{H\times H\times S\times Q}$ is the "projected distance tensor", which is derived from $\boldsymbol{F}$ by projecting its representation (with homologues domain copies) to its counterpart without homologous domain distinction. $CN(I)$ and $CN(J)$ are the homologues copy numbers of domain I and J, respectively; 2 for autosomal chromosomes and 1 for sex chromosomes. Overall $\bar{f}_{IJsq}$ can take values $\{0,1\}$ or $\{0,1,2\}$ or $\{0,1,2,3,4\}$ depending on the total number of homologous domain pairs associated with (I, J) that are contact in the population. For instance, in the projected distance tensor $\bar{\boldsymbol{F}}$, each element $\bar{f}_{IJqs} = 1$ indicates that any one of two homologues copies of two domains I and J have a distance in the range defined by bin $\mathcal{B}_q = [d_q, d_{q+1})$ in structure s. while $\bar{f}_{IJqs} = 2$ indicates that two out of 4 possible pairs of homologues chromatin domains I and J have a distance in the range defined by bin $\mathcal{B}_q = [d_q, d_{q+1})$ in structure s.

We then model each m_{IJq} as $m_{IJq} = m'_{IJq} + \varepsilon_{IJq}$, where ε_{IJq} are independent and identical normally distributed random variables with mean zero and $\varepsilon_{IJq} =\propto \mathcal{N}(0, \sigma_{IJq}^2)$. (ε_{IJq} is effectively set to 0).

<u>Chromatin contact probabilities from Hi-C data (bivariate data)</u>

<u>Expansion of $P(\boldsymbol{W}|\boldsymbol{X})$</u>

We introduced the latent contact indicator tensor $\boldsymbol{W} = (w_{ijs})_{N\times N\times S}$ for complementing every single cell's contact information. $w_{ijs} = 1$ indicates that a contact is present between chromatin domains i and j in structure s; $w_{ijs} = 0$ otherwise.

$$p\left(w_{ijs}|\vec{\boldsymbol{x}}_{is}, \vec{\boldsymbol{x}}_{js}\right) = p\left(w_{ijs} = 1|\vec{\boldsymbol{x}}_{is}, \vec{\boldsymbol{x}}_{js}\right)^{w_{ijs}}\left[1 - p\left(w_{ijs} = 1|\vec{\boldsymbol{x}}_{is}, \vec{\boldsymbol{x}}_{js}\right)\right]^{1-w_{ijs}}$$

We approximate $P(\boldsymbol{W}|\boldsymbol{X})$ as

$$P(\boldsymbol{W}|\boldsymbol{X}) = \prod_{i,j,s} p\left(w_{ijs}|\vec{\boldsymbol{x}}_{is}, \vec{\boldsymbol{x}}_{js}\right)$$

We assume a pair of chromatin domains (i, j) have a contact in structure s, if and only if $\left\|\vec{\boldsymbol{x}}_{is} - \vec{\boldsymbol{x}}_{js}\right\|_2 \leq 2(R_i^{ex} + R_j^{ex})$. R_i^{ex} is the excluded volume radius of sphere i *(Supplementary Information)*. Following Tjong *et al.*[1] we have:

$$p(w_{ijs} = 1|\vec{\boldsymbol{x}}_{is}, \vec{\boldsymbol{x}}_{js}) = \begin{cases} \exp\left[-\frac{\left(\left\|\vec{\boldsymbol{x}}_{is} - \vec{\boldsymbol{x}}_{js}\right\|_2 - 2(R_i^{ex} + R_j^{ex})\right)^2}{2\sigma_w^2}\right], & if \left\|\vec{\boldsymbol{x}}_{is} - \vec{\boldsymbol{x}}_{js}\right\|_2 - 2(R_i^{ex} + R_j^{ex}) \geq 0 \\ 1, & otherwise \end{cases}$$

Expansion of $P(\boldsymbol{A}|\boldsymbol{W}, \boldsymbol{X})$

Given $\boldsymbol{A} = (a_{IJ})_{H\times H}$ as the contact probability derived from Hi-C data, also $P(\boldsymbol{A}|\boldsymbol{W})$ can be expanded as $P(\boldsymbol{A}|\boldsymbol{W}, \boldsymbol{X}) = \prod_{I,J} P(a_{IJ}|a'_{IJ})$, where a'_{IJ} is the contact probability of the genomic regions I and J computed from $\boldsymbol{W}$ as

$$a'_{IJ} = \frac{1}{\min\,(CN(I), CN(J))} \cdot \frac{1}{S}\sum_{s=1}^{S} \bar{w}_{IJs}$$

where $\overline{\boldsymbol{W}} = (\bar{w}_{IJs})_{H\times H\times S}$ is the “projected contact tensor”, which is derived from $\boldsymbol{W}$ by projecting its representation (with homologues domain copies) to its counterpart without homologous domain distinction. $CN(I)$ and $CN(J)$ are the homologues copy numbers of domain I and J, respectively; 2 for autosomal chromosomes and 1 if I or J are part of the sex chromosomes in a male cell. Overall $\bar{w}_{IJs}$ can take values $\{0,1\}$ or $\{0,1,2\}$ or $\{0,1,2,3,4\}$ depending on the total number of homologous chromatin domain pairs associated with (I, J). For instance, in the projected tensor $\overline{\boldsymbol{W}}$, each element $\bar{w}_{IJs} = 1$ indicates that any one of two homologues copies of two domains I and J have contact in structure s. $\bar{w}_{IJs} = 2$ indicates that two out of 4 possible pairs made by diploid homologues copies of two domains I and J have contacts in structure s.

We then model each a_{IJ} as $a_{IJ} = a'_{IJ} + \varepsilon_{IJ}$, where ε_{IJ} are independent and identical normally distributed random variables with mean zero and $\varepsilon_{IJ} =\propto \mathcal{N}(0, \sigma_{IJ}^2)$. (ε_{IJ} is effectively set to 0).

Chromatin multi-way contacts from single cell SPRITE data (multivariate data)

Expansion of $P(\boldsymbol{R}|\boldsymbol{X})$

We introduced the latent indicator tensor $\boldsymbol{R}^n = (r_{i_1,\dots,i_n,s})_{N^n\times S}$ to complement SPRITE single-cell multi-way colocalization of all clusters C_n of n loci. $r_{i_1,\dots,i_n,s}$ indicates whether chromatin domains $\{i_1, \dots, i_n\}$ are co-localized in structure s ($r_{i_1,\dots,i_n,s} = 1$) or not ($r_{i_1,\dots,i_n,s} = 0$).

Since in principle any data point is independent on others:

$$P(\boldsymbol{R}^n|\boldsymbol{X}) = \prod_{C_n}\prod_s p_n\left(r_{i_1,\dots,i_n,s}|\vec{\boldsymbol{x}}_{i_1s},\vec{\boldsymbol{x}}_{i_2s},\dots,\vec{\boldsymbol{x}}_{i_ns}\right)$$

The product $\prod_{C_n}$ runs over the different clusters with n loci. The probability of multi-way colocalization reads:

$$p_n\left(r_{i_1,\dots,i_n,s}|\vec{\boldsymbol{x}}_{i_1s},\vec{\boldsymbol{x}}_{i_2s},\dots,\vec{\boldsymbol{x}}_{i_ns}\right) = p_n\left(r_{i_1,\dots,i_n,s}|\vec{\boldsymbol{x}}_{i_1s},\vec{\boldsymbol{x}}_{i_2s},\dots,\vec{\boldsymbol{x}}_{i_ns}\right)^{r_{i_1,\dots,i_n,s}}\left[1-p_n\left(r_{i_1,\dots,i_n,s}|\vec{\boldsymbol{x}}_{i_1s},\vec{\boldsymbol{x}}_{i_2s},\dots,\vec{\boldsymbol{x}}_{i_ns}\right)\right]^{1-r_{i_1,\dots,i_n,s}}$$

The loci that co-localize define a cluster geometric center $\vec{\boldsymbol{x}}_{Gs} = \frac{1}{n}\sum_{g=1,\dots,n}\vec{\boldsymbol{x}}_{i_gs}$. We write the probability as:

$$p_n\left(r_{i_1,\dots,i_n,s}|\vec{\boldsymbol{x}}_{i_1s},\vec{\boldsymbol{x}}_{i_2s},\dots,\vec{\boldsymbol{x}}_{i_ns}\right) = \prod_{g=1,\dots,n}\begin{cases}\exp\left[-\dfrac{\left(\left\|\vec{\boldsymbol{x}}_{i_gs}-\vec{\boldsymbol{x}}_{Gs}\right\|_2 - C^{SPRITE}\right)^2}{2\sigma_r^2}\right], & if\ \left\|\vec{\boldsymbol{x}}_{i_gs}-\vec{\boldsymbol{x}}_G\right\|_2 \geq C^{SPRITE} \\ 1, & otherwise\end{cases}$$

C^{SPRITE} is the cluster radius, which we define in the *Supplementary Information*, and g runs over the participating loci. In our modeling, multi-way clusters are interpreted as a product of pairwise contacts between each of the participating loci and the cluster centroid.

Expansion of $P(\boldsymbol{T}^n|\boldsymbol{R}^n,\boldsymbol{X})$

Given $\boldsymbol{T}^n = \left(t_{I_1,\dots I_n}\right)_{H^n}$ as the multi-way contact probability of n-loci derived from single cell SPRITE data, we can write

$$P(\boldsymbol{T}^n|\boldsymbol{R}^n,\boldsymbol{X}) = \prod_{C_n}\prod_{I_1,\dots,I_n} p\left(t_{I_1,\dots,I_n}\middle|t'_{I_1,\dots,I_n}\right), \ ,$$

where $t'_{I_1,\dots,I_n}$ is the multi-way contact probability of the genomic regions $I_1,\dots,I_n$ computed from $\boldsymbol{R}^n$ as

$$t'_{I_1,\dots,I_n} = \frac{1}{\min\left(CN(I_1),\dots,CN(I_n)\right)}\frac{1}{S}\sum_s \bar{r}_{I_1,\dots,I_n,s}$$

where $\overline{\boldsymbol{R}}^n = \left(\bar{r}_{I_1,\dots,I_n,s}\right)_{H^n \times S}$ is the "projected multi-way contact tensor", which is derived from $\boldsymbol{R^n}$ by projecting its representation (with homologues domain copies) to its counterpart without homologous domain distinction. $CN(I_1)$ is the homologue copy number of domain I_1. Overall $\bar{r}_{I_1,\dots,I_n,s}$ can take a spectrum of integer values from 0 to 2^n depending on the total number of homologous chromatin domain pairs associated with $(I_1,\dots,I_n)$ which colocalize.

We then model each $t_{I_1,\dots I_n}$ as $t_{I_1,\dots I_n} = t'_{I_1,\dots I_n} + \varepsilon_{I_1,\dots I_n}$, where $\varepsilon_{I_1,\dots I_n}$ are independent and identical normally distributed random variables with mean zero and $\varepsilon_{I_1,\dots I_n} =\propto \mathcal{N}(0,\sigma^2_{I_1,\dots I_n})$ ($\varepsilon_{I_1,\dots I_n}$ is effectively set to 0).

<u>All clusters with arbitrary number of loci</u>

The formulation can be easily extended to account for clusters of different sizes n. Each $\boldsymbol{T}^n$ is associated with a $\boldsymbol{R}^n$, so for example:

$$P(\{\boldsymbol{R}^n\}|\boldsymbol{X}) = \prod_n \prod_{C_n} \prod_s p_n\left(r_{i_1,\dots,i_n,s}|\vec{\boldsymbol{x}}_{i_1s}, \vec{\boldsymbol{x}}_{i_2s}, \dots, \vec{\boldsymbol{x}}_{i_ns}\right)$$

$$P(\{\boldsymbol{T}^n\}|\{\boldsymbol{R}^n\},\boldsymbol{X}) = \prod_n \prod_{C_n} \prod_{I_1,\dots,I_n} p\left(t_{I_1,\dots,I_n}|t'_{I_1,\dots,I_n}\right),$$

where we multiply over the different cluster sizes. Overall, the notation $\prod_n \prod_{C_n}$ denotes the product over all individual clusters.

Gathering all the terms – formulation of the conditional probability function

With all probabilistic models in place we can now maximize the log-likelihood (see also **Equation 1**),

$$logP(\boldsymbol{U},E,\boldsymbol{M},\boldsymbol{A},\boldsymbol{T},\boldsymbol{B},\boldsymbol{V},\boldsymbol{F},\boldsymbol{W},\boldsymbol{R}|\boldsymbol{X}) = \log[P(\boldsymbol{U}|\boldsymbol{B},\boldsymbol{X})P(E|\boldsymbol{V},\boldsymbol{X})P(\boldsymbol{M}|\boldsymbol{F},\boldsymbol{X})P(\boldsymbol{A}|\boldsymbol{W},\boldsymbol{X})P(\boldsymbol{T}|\boldsymbol{R},\boldsymbol{X})P(\boldsymbol{B},\boldsymbol{V},\boldsymbol{F},\boldsymbol{W},\boldsymbol{R}|\boldsymbol{X})]$$

$$
\begin{aligned}
&logP(\boldsymbol{U}, E, \boldsymbol{M}, \boldsymbol{A}, \boldsymbol{T}, \boldsymbol{B}, \boldsymbol{V}, \boldsymbol{F}, \boldsymbol{W}, \boldsymbol{R} | \boldsymbol{X}) \\
&= \log[P(\boldsymbol{U}|\boldsymbol{B},\boldsymbol{X})P(E|\boldsymbol{V},\boldsymbol{X})P(\boldsymbol{M}|\boldsymbol{F},\boldsymbol{X})P(\boldsymbol{A}|\boldsymbol{W},\boldsymbol{X})P(\boldsymbol{T}|\boldsymbol{R},\boldsymbol{X})P(\boldsymbol{B},\boldsymbol{V},\boldsymbol{F},\boldsymbol{W},\boldsymbol{R}|\boldsymbol{X})] \\
&= \sum_{q=1}^{Q}\sum_{I=1}^{H}\log P\left(u_{Iq}|u'_{Iq}\right) + + \sum_{I=1}^{H}\log P(e_I|e'_I) + \sum_{q=1}^{Q}\sum_{I=1}^{H}\sum_{J>I}^{H}\log P(m_{IJq}|m'_{IJq}) \\
&+ \sum_{I=1}^{H}\sum_{J>I}^{H}\log P\left(a_{IJ}|a'_{IJ}\right) + \sum_{n}\sum_{C_n}\log P(t_{I_1,\dots,I_n}|t'_{I_1,\dots,I_n}) + \sum_{q=1}^{Q}\sum_{s=1}^{S}\sum_{I=1}^{N}\log p\left(b_{iqs}|\vec{\boldsymbol{x}}_{is}\right) \\
&+ \sum_{s=1}^{S}\sum_{i=1}^{N}\log p(v_{is}|\vec{\boldsymbol{x}}_{is}) + \sum_{q=1}^{Q}\sum_{s=1}^{S}\sum_{i=1}^{N}\sum_{j>i}^{N}\log p\left(f_{ijqs}|\vec{\boldsymbol{x}}_{is},\vec{\boldsymbol{x}}_{js}\right) + \\
&+ \sum_{s=1}^{S}\sum_{i=1}^{N}\sum_{j>i}^{N}\log p\left(w_{ijs}|\vec{\boldsymbol{x}}_{is},\vec{\boldsymbol{x}}_{js}\right) + \sum_{s}\sum_{n}\sum_{C_n}\log p_n\left(r_{i_1,\dots,i_n,s}|\vec{\boldsymbol{x}}_{i_1 s},\vec{\boldsymbol{x}}_{i_2 s},\dots,\vec{\boldsymbol{x}}_{i_n s}\right)
\end{aligned}
$$

(**Eq. S2**)

In addition to the 5 data sources from 4 experimental methods (Supplementary **Table S1**), we also include a set of spatial constraints based on additional information about the genome organization. These data are included in form of general spatial constraints acting on N chromatin domain domains: (i) a nuclear volume confinement restraint that forces all chromatin domains to be inside the nuclear volume, (ii) excluded volume restraints that prevent “hard core” overlap between any two chromatin domains and (iii) a polymer chain connectivity restraint between chromatin domain neighbors in a chromosome, which guarantees the structural integrity of the chromosomal chains. Additional information about these restraints is given in the *Supporting Information*.

In summary, the maximum likelihood problem is formally expressed as follows,

$$
\widehat{\boldsymbol{X}}, \widehat{\boldsymbol{B}}, \widehat{\boldsymbol{V}}, \widehat{\boldsymbol{F}}, \widehat{\boldsymbol{W}}, \widehat{\boldsymbol{R}} = \arg\max_{\mathbf{X,V,B,W,F,R}}\{\log P(\boldsymbol{U}, E, \boldsymbol{M}, \boldsymbol{A}, \boldsymbol{T}, \boldsymbol{B}, \boldsymbol{V}, \boldsymbol{F}, \boldsymbol{W}, \boldsymbol{R}|\boldsymbol{X})\} \quad \textbf{(Eq. S3)}
$$

$$
subject\ to \begin{cases} nuclear\ volume\ constraint \\ excluded\ volume\ constraint \\ chain\ connectivity\ restraint \end{cases}
$$

We will now detail how the optimization is performed by describing the Assignment and Modeling steps of the optimizations as implemented in the Integrated Genome Modeling platform for each data source.

2. A/M optimization: Assignment Step

Starting from the current population of structures $\boldsymbol{X}^{(t)}$, which resulted from a previous A/M optimization iteration, the optimal latent variables $\boldsymbol{B}^{t+1}, \boldsymbol{V}^{t+1}, \boldsymbol{F}^{t+1}, \boldsymbol{W}^{t+1}, \boldsymbol{R}^{t+1}$ for the following Modeling Step (see *Methods*) are fully determined by solving:

$$\boldsymbol{B}^{t+1}, \boldsymbol{V}^{t+1}, \boldsymbol{F}^{t+1}, \boldsymbol{W}^{t+1}, \boldsymbol{R}^{t+1}$$
$$= \arg max_{\boldsymbol{B},\boldsymbol{V},\boldsymbol{F},\boldsymbol{W},\boldsymbol{R}} \log[P(\boldsymbol{U}|\boldsymbol{B},\boldsymbol{X}^{t})P(E|\boldsymbol{V},\boldsymbol{X}^{t})P(\boldsymbol{M}|\boldsymbol{F},\boldsymbol{X}^{t})P(\boldsymbol{A}|\boldsymbol{W},\boldsymbol{X}^{\boldsymbol{t}})P(\boldsymbol{T}|\boldsymbol{R},\boldsymbol{X}^{t})P(\boldsymbol{B},\boldsymbol{V},\boldsymbol{F},\boldsymbol{W},\boldsymbol{R}|\boldsymbol{X}^{t})]$$

For the sake of generality, we will assume each genomic region I comes with two copies (i, i'), but the discussion can easily be adjusted for single copy loci.

Also, let $\sigma[k{:}K]$ indicate the ranking function that returns the rank of the element $k \in K$ when the t-uple K is sorted in ascending order.

3D radial HIPMap FISH data

Assume the distributions of radial distances have been already assigned into distance bins $\{\mathcal{B}_q\}$ and cast into the $\boldsymbol{U}_{H\times Q}$ variable. By using the information in the data $\boldsymbol{U}_{H\times Q}$ and the population $\boldsymbol{X}^{(t)}$, we determine the optimal latent variable $\boldsymbol{B}^{t+1}_{N\times Q\times S}$. (we will drop the $t+1$ superscript in the following, since there is no ambiguity).

Let us denote with U_I all the entries of matrix $\boldsymbol{U}$ that are associated with genomic locus I. If $\{x_s^I |\, s = 1, \dots, S\}$ denotes the set of discrete distance values (from experiments for example) that define the minimal radial distribution of region I (*Methods*), those distances are mapped onto bins that are identified by the integers $Q_U^I = \left\{q_{d=x_k^I}\right\}$, such that $x_1^I \le x_2^I \le \cdots$ (recall that the integer $q_{d=\bar{d}}$ labels the distance bin into which distance $\bar{d}$ falls, e.g. $\bar{d} \in \mathcal{B}_{q_{d=\bar{d}}}$). U_I is non-zero on those bins only.

We compute all the radial distances of both copies (i, i') in all structures of the population $\boldsymbol{X}^t$, and select the S minimal distances

$$Z_I^{min} = \{\min\{\|\vec{\boldsymbol{x}}_{is}\|_2, \|\vec{\boldsymbol{x}}_{i's}\|_2\}\ |s = 1, \dots S\}$$

and sort them in ascending order; we obtain a sequence of ordered (copy of domain I, structure index) pairs:

$H_I = \{(i_1, s_1), \cdots, (i_S, s_S)\}$, such that $\|\vec{\boldsymbol{x}}_{i_n s_n}\|_2 \leq \|\vec{\boldsymbol{x}}_{i_m s_m}\|_2, \forall\, n \leq m.$

The latent variable is then populated as follows:

$$b_{iqs} = \begin{cases} 1, \ \ if\ q \in Q_U^I, \ \ and\ \sigma[(i,s){:}\,H_I] = \sigma[q{:}\,Q_U^I] \\ \qquad\qquad 0, \qquad\qquad otherwise \end{cases}, \qquad \textbf{(Eq. S4)}$$

Basically, $b_{iqs} = 1$ only if the pair (i, s) has the same rank in the list of ordered pairs H_I as the qth distance bin does in the list of ordered distance bins Q_U^I . If ranks are different or q does not label a target distance bin (i.e., $q \notin Q_U^I, \}$), then $b_{iqs} = 0$.

The same procedure applies to assign distributions of radial maximal distances.

This maximizes the probability $\log P(\boldsymbol{U}, \boldsymbol{B}|\boldsymbol{X}) = \log P(\boldsymbol{U}|\boldsymbol{B}, \boldsymbol{X}) \log P(\boldsymbol{B}|\boldsymbol{X})$. Sorting both the distances in the models and in the input and running a one-to-one assignment makes sure that deviations from the target distances are smallest (maximizing $\log P(\boldsymbol{B}|\boldsymbol{X})$) and also makes u_{Iq} equal to u'_{Iq}, therefore maximizing $\log P(\boldsymbol{U}|\boldsymbol{B}, \boldsymbol{X})$ (see *Methods*).

Lamina DamID:

By using the information in the data $E_{H\times 1}$ and the population $\boldsymbol{X}^{(t)}$, we determine the optimal latent variable $\boldsymbol{V}_{N\times S}^{t+1}$.

We compute the distances of both copies of domain I to the nuclear envelope (NE) $\{d(\vec{\boldsymbol{x}}_{is}, NE), d(\vec{\boldsymbol{x}}_{i's}, NE) \mid s = 1, \ldots, S\})$ from population $\boldsymbol{X}^{(t)}$, and sort them in ascending order. The $(2S \cdot e_I)th$ ranked value is chosen to be a lamina activation distance d_I^{act}. The latent variable is then populated as follows:

$$v_{is} = \begin{cases} \ 1 \ \ if\ d(\vec{\boldsymbol{x}}_{is}, NE)\ \geq d_I^{act} \\ \ 0 \ \ \ otherwise \end{cases} \qquad \textbf{(Eq. S5)}$$

by counting the number of all pooled distances that are larger than the activation distance. Computing the distance to the lamina is detailed later in the “A/M optimization: Modeling Step” section.

This procedure maximizes $\log p(E, \boldsymbol{V}|\boldsymbol{X}) = \log P(E|\boldsymbol{V}, \boldsymbol{X}) \cdot P(\boldsymbol{V}|\boldsymbol{X})$. This is true for two reasons. It assigns contacts only to domains with shortest distances to the lamina, therefore maximizing

$P(\boldsymbol{V}|\boldsymbol{X})$; also, the distance threshold method heuristically maximizes the first term $\log P(E|\boldsymbol{V},\boldsymbol{X}) = \sum_I \log p(e_I, e_I')$ by making e_I equal to e_I'. (see also Li *et al.*[2])

3D pairwise HIPMap FISH data

Assume the distributions of pairwise distances have already been assigned into distance bins $\{\mathcal{B}_q\}$ and cast into the $\boldsymbol{M}_{H\times H\times Q}$ variable. By using the information in the data $\boldsymbol{M}_{H\times H\times Q}$ and the population $\boldsymbol{X}^{(t)}$, we determine the optimal latent variable $\boldsymbol{F}^{t+1}_{N\times N\times Q\times S}$.

Let us denote with M_{IJ} all the entries of matrix $\boldsymbol{M}$ that are associated with the genomic regions I and J (with homologue pairs (i,i') and (j,j') respectively). If $\{y_s^{IJ}|s = 1,\dots,S\}$ denotes the set of discrete distance values (from experiments for example) that define the minimal pairwise distance distribution of the pair (I,J), those distances are mapped onto bins that are identified by the integers $Q_M^{IJ} = \left\{q_{d=y_1^{IJ}}\right\}$, such that $y_1^{IJ} \le y_2^{IJ} \le \cdots$ (recall that the integer $q_{d=\bar{d}}$ labels the distance bin into which distance $\bar{d}$ falls, e.g. $\bar{d} \in \mathcal{B}_{q_{d=\bar{d}}}$). M_{IJ} is non-zero on those bins only.

We compute all the distances between pairs of loci $(i,j),(i,j'),(i',j)$ and (i',j') in all structures of the population $\boldsymbol{X}^t$, and select the minimal distances

$$Z_{IJ}^{min} = \left\{\min\left\{\|\vec{\boldsymbol{x}}_{is} - \vec{\boldsymbol{x}}_{js}\|_2, \|\vec{\boldsymbol{x}}_{is} - \vec{\boldsymbol{x}}_{j's}\|_2, \|\vec{\boldsymbol{x}}_{i's} - \vec{\boldsymbol{x}}_{js}\|_2, \|\vec{\boldsymbol{x}}_{i's} - \vec{\boldsymbol{x}}_{j's}\|_2\right\} \middle| s = 1,\dots S\right\}$$

and sort them in ascending order: we obtain a sequence of ordered (copy of region I, copy of region J, structure index) triplets:

$$H_{IJ} = \{(i_1,j_1,s_1),(i_2,j_2,s_2),\dots\} : \ \|\vec{\boldsymbol{x}}_{i_n s_n} - \vec{\boldsymbol{x}}_{j_n s_n}\|_2 \le \|\vec{\boldsymbol{x}}_{i_m s_m} - \vec{\boldsymbol{x}}_{j_m s_m}\|_2, \forall\, n \le m,$$

The latent variable is the populated as follows:

$$f_{ijqs} = \begin{cases} 1, & if\ q \in Q_M^{IJ},\ \ and\ \sigma\left[(i,j,s): H_{IJ}\right] = \sigma[q: Q_M^{IJ}] \\ 0, & otherwise \end{cases} \qquad \textbf{(Eq. S6)}$$

Basically, $f_{ijqs} = 1$ only if the triplet (i,j,s) has the same rank in the list of ordered triplets H_{IJ} as the qth distance bin does in the list of ordered distance bins Q_M^{IJ}. If ranks are different or q does not label a target distance bin (i.e., $q \notin Q_M^{IJ}$), then $f_{ijqs} = 0$.

This maximizes the probability $\log P(\boldsymbol{M},\boldsymbol{F}|\boldsymbol{X}) = \log P(\boldsymbol{M}|\boldsymbol{F},\boldsymbol{X}) \cdot \log P(\boldsymbol{F}|\boldsymbol{X})$. Sorting both the distances in the models and in the input and running a one-to-one assignment makes sure that deviations from the target distances are smallest (maximizing $\log P(\boldsymbol{F}|\boldsymbol{X})$) and also makes m_{IJq} equal to m'_{IJq}, see *Methods*.

Ensemble Hi-C

By using the information in the data $\boldsymbol{A}_{H\times H}$ and the population $\boldsymbol{X}^{(t)}$, we determine the optimal latent variable $\boldsymbol{W}_{N\times N\times S}$.

If I and J are intra-chromosomal domains, only (i,j) and (i',j') pairwise distances in each structure across the population $\boldsymbol{X}^{(t)}$ are computed; if I and J are inter-chromosomal domains, all the $(i,j),(i,j'),(i',j)$ and (i',j') pairwise distances in each structure across the population $\boldsymbol{X}^{(t)}$ are computed. We then compute and sort all those pairwise distances. The $(2S\cdot a_{IJ})$-th $((4S\cdot a_{IJ}) - th,)$ ranked distance value is chosen as contact activation distance d_{IJ}^{act}. The latent variable is populated as follows:

$$w_{ijs} = \begin{cases} 1, & if \ \|\vec{\boldsymbol{x}}_{is} - \vec{\boldsymbol{x}}_{js}\|_2 \le d_{IJ}^{act} \\ 0, & otherwise \end{cases} \qquad \textbf{(Eq. S7)}$$

by counting the number of all pooled distances that are shorter than the corresponding activation distance.

This procedure maximizes $\log p(\boldsymbol{A},\boldsymbol{W}|\boldsymbol{X}) = \log P(\boldsymbol{A}|\boldsymbol{W},\boldsymbol{X}) \cdot P(\boldsymbol{W}|\boldsymbol{X})$. This is true for two reasons. It assigns contacts only to locus pairs with shortest distances, therefore maximizing $P(\boldsymbol{W}|\boldsymbol{X})$; also, the distance threshold method heuristically maximizes the first term $\log P(\boldsymbol{A}|\boldsymbol{W},\boldsymbol{X}) = \sum_I \log p(a_{IJ}, a'_{IJ})$ by making a_{IJ} equal to a'_{IJ}. (See also Tjong *et al.*[1])

SPRITE

Using the information in the data $\boldsymbol{T}^n = \left(t_{I_1,\dots,I_n}\right)_{H^n}$ and the population $\boldsymbol{X}^{(t)}$, we determine the latent variables $\boldsymbol{R}^n = (r_{i_1,\dots,i_n,s})_{N^n\times S}$. For convenience, we will discuss Assignment for one cluster $C_n = (I_1,\dots,I_n)$. There are two steps: first, we determine which copies of the genomic regions make up the cluster, and then to which structure in the population the cluster is assigned.

Let us assume that the cluster involves $n_{chr} \leq n$ different chromosomes, and that all chromosomes come with two homologous copies. The two copies associated with domain I_1 will be uniquely identified as $(I_1, h = 1)$ and $(I_1, h = 2)$. To avoid complications due to number of all possible diploid representations of a size n cluster scaling as 2^n, we additionally assume that loci mapping to the same chromosome are also physically on the same copy. We then solve the minimization:

$$\begin{cases} \hat{h}_1, \ldots, \hat{h}_n = \arg \min_{h_1, \ldots, h_n} R_g^2\left[C_n^{dipl}, s\right] \\ s.t.\, h_k = h_p, if\ I_k\ and\ I_p\ from\ same\ chrom \end{cases}$$

Where h_k and h_p indicate the copy indexes. The optimal copy indexes are those which minimize the radius of gyration $R_g[C_n^{dipl}, s]$ of the cluster in structure s among all its possible diploid representations, with the additional restraint that chromatin regions on the same chromosomes are also physically on the same copy. Since the number of different configurations of indexes to explore ($2^{n_{chr}}$) is equal or smaller than 2^n, looking for the minimal radius of gyration occurs by exhaustively enumerating the different $(h_1, \ldots, h_n)$. The diploid representation of the cluster (in structure s) will then be $\tilde{C}_n = \{(I_1, \hat{h}_1), \ldots, (I_n, \hat{h}_n)\}$, see also **Table S2**.

	Chrom. number	First copy	Second copy
I_1	1	1	201
I_2	1	2	202
I_3	2	20	220

h_1	h_2	h_3	Diploid repr. of the cluster	$R_g\left[C_3^{dipl}\right]$ [a.u.]
1	1	1	{1,2,20}	102.6
1	1	2	{1,2,220}	33.2
2	2	1	{201,202,20}	155

2	2	2	$\{201,202,220\}$	300

Table S2 A two chromosome toy model is used to demonstrate how the Assignment goes from single cell cluster C_n to its diploid representation $\tilde{C}_n$. Assume $C_3 = \{I_1 = 1, I_2 = 2, I_3 = 20\}$, with domains mapping to chromosomes $\{1,1,2\}$ respectively. We then have six different loci in when copies are distinguished (top table). We compute the radius of gyration $R_g[C_3^{dipl}]$ for all combinations (bottom table) in a given structure, by forcing regions from the same chromosome to have the same copy index h. The minimal radius of gyration is obtained for $(\hat{h}_1 = 1, \hat{h}_2 = 1, \hat{h}_3 = 2)$, so the diploid representation of cluster C_3 in that structure is $\tilde{C}_3 = \{1,2,220\}$.

The Assignment step concludes with assigning the cluster to one structure in the population $\boldsymbol{X}^{(t)}$. We collect optimal diploid representations $\tilde{C}_n[s]$ of the cluster in each structure of the population. In order to avoid overloading a few structures with many clusters, we draw the optimal structure index from the Gibbs distribution:

$$P_{C_n}^{SPRITE}(s) = \frac{1}{Z}\exp\left[-\frac{R_g\left[\tilde{C}_n[s], s\right]}{kT} + \omega_s\right], \quad Z = \sum_s P_{C_n}^{SPRITE}(s)$$

$R_g[\tilde{C}_n[s], s]$ indicates the radius of gyration of the optimal diploid representation of the cluster in structure s, and kT is a normalization parameter. A penalization $\omega_s = \frac{\theta(occ_s - \langle n \rangle)}{\sqrt{\langle n \rangle}}$ is introduced, where occ_s is the integer number of clusters already assigned to current structure s ("structure occupancy"), θ is the Heaviside step function and $\langle n \rangle = S/n_{cl}$ is the ratio between number of structures in the population and the total number of clusters to be assigned. Note that a non-zero penalization is applied only when the current structure occupancy is larger than $\langle n \rangle$.

With all of this in mind, the SPRITE latent variable is finally populated as follows:

$$r_{i_1,\dots i_n,s} = \begin{cases} 1, & if\ \tilde{C}_n[s] = \{i_1, \dots i_n\}\ and\ s \sim P_{C_n}^{SPRITE}] \\ 0, & otherwise \end{cases} \quad \textbf{(Eq. S8)}$$

Basically, for each cluster of size n, $r_{i_1,\dots i_n,s} = 1$ only if the n-tuple $(i_1, \dots i_n)$ is the optimal diploid representation of the cluster (in the sense discussed above) in structure s, which is drawn from the probability distribution $P_n^{SPRITE}(s)$.

This assignment procedure heuristically maximizes the log likelihood $\log p(\boldsymbol{T}^n, \boldsymbol{R}^n|\boldsymbol{X}) = \log P(\boldsymbol{T^n}|\boldsymbol{R}^n, \boldsymbol{X}) \cdot P(\boldsymbol{R^n}|\boldsymbol{X})$, see *Methods*.

3. A/M optimization: Modeling Step

Starting from the current latent variables $\boldsymbol{B}^{t+1}, \boldsymbol{V}^{t+1}, \boldsymbol{F}^{t+1}, \boldsymbol{W}^{t+1}, \boldsymbol{R}^{t+1}$ which resulted from a previous A-step (see **Equations S4-S8**), the optimal coordinates $\boldsymbol{X}^{t+1} = (\boldsymbol{X}_1^{t+1}, \dots, \boldsymbol{X}_s^{t+1})$ of all the diploid genome structures for the following A/M iteration are fully determined by solving:

$$\boldsymbol{X}^{t+1} = \arg\max_{\mathbf{x}} \log[P(\boldsymbol{U}|\boldsymbol{B}^{t+1}, \boldsymbol{X})P(E|\boldsymbol{V}^{t+1}, \boldsymbol{X})P(\boldsymbol{M}|\boldsymbol{F}^{t+1}, \boldsymbol{X})P(\boldsymbol{A}|\boldsymbol{W}^{t+1}, \boldsymbol{X})P(\boldsymbol{T}|\boldsymbol{R}^{t+1}, \boldsymbol{X})P(\boldsymbol{B}^{t+1}, \boldsymbol{V}^{t+1}, \boldsymbol{F}^{t+1}, \boldsymbol{W}^{t+1}, \boldsymbol{R}^{t+1}|\boldsymbol{X})]$$

The effect of each non-zero latent variable entry is modeled as an appropriate energy term (i.e., restraint), which is added to the genome energy function, and which is compatible with the probability representation of the data sources given in the *Methods*. Each energy term involves one (univariate), two (bivariate) or more (multivariate) chromatin loci, and actively constrains their distances; each interaction is associated with a positive scalar (residual error) η which monitors to what extent a given restraint is violated. Specifically, $\eta > 0.05$ indicates that a given distance in the model is off by more than 5% with respect to the expected distance from the input: we will call that a violation in the model. We now detail the form of the energy terms we use to model each data source.

<u>3D radial HIPMap FISH data</u>

$b_{iqs} = 1$ indicates that locus i must have radial distance from nuclear center in distance bin $[d_q, d_{q+1})$ far from the center in structure s. We enforce that by a combination of truncated harmonic potentials with a tolerance t (*Methods*, **Extended Data Figures 1** and **2**):

$$\mathcal{U}_{iqs}^b = \frac{k^b}{2}\begin{cases} [\|\vec{\boldsymbol{x}}_{is}\|_2 - (d_q - t)]^2, & if\ \|\vec{\boldsymbol{x}}_{is}\|_2 \le d_q - t \\ 0, & if\ \ d_q - t \le \|\vec{\boldsymbol{x}}_{is}\|_2 \le d_q + t \\ [\|\vec{\boldsymbol{x}}_{is}\|_2 - (d_q + t)]^2, & if\ \|\vec{\boldsymbol{x}}_{is}\|_2 \ge d_q + t \end{cases}$$

The residual error ($0 \le \eta \le\ 1$) is defined as a ratio of distances:

$$\eta_{iqs}^{b}=\begin{cases}\frac{\|\vec{\boldsymbol{x}}_{is}\|_2}{d_q-t}-1\,, & if\ \|\vec{\boldsymbol{x}}_{is}\|_2\le d_q-t\\ 0 & if\ \ d_q-t\le\|\vec{\boldsymbol{x}}_{is}\|_2\le d_q+t\\ \frac{\|\vec{\boldsymbol{x}}_{is}\|_2}{d_q+t}-1, & if\ \|\vec{\boldsymbol{x}}_{is}\|_2\ge d_q+t\end{cases}$$

<u>Lamina DamID</u>

$v_{is}=1$ indicates that locus i is in contact with the lamina in structure s. So, we apply an ellipsoidal lower harmonic bound (*Methods*, see also volumetric confinement below):

$$\mathcal{U}_{is}^{v}=\begin{cases}\frac{k^v}{2}\left(\frac{1}{\sqrt{\kappa_{is}(r_0,c_r)}}-1\right)^2\|\vec{\boldsymbol{x}}_{is}\|_2{}^2, & if\ \kappa_{is}\le 1\\ 0 & otherwise\end{cases},$$

$$\kappa_{is}(r,c_r)=\frac{x_{is}^2}{[a(1-c_r)-r_0]^2}+\frac{y_{is}^2}{[b(1-c_r)-r_0]^2}+\frac{z_{is}^2}{[b(1-c_r)-r_0]^2}$$

c_r being a positive scalar (this is our approximation to the lamina contact threshold, see *Methods*): $c_r=0$ implies that contact is only formed when the genome locus is physically in contact with the envelope.

The residual error is here evaluated as the ratio between the distance of a locus to the lamina and its radial distance from the center:

$$\eta_{is}^{v}=\begin{cases}\left(1-\sqrt{\kappa_{is}}\right), if\ \kappa_{is}<1\\ 0\end{cases}$$

and is non-zero when the locus is not in the outer contact shell.

<u>3D pairwise HIPMap FISH data</u>

$f_{ijqs}=1$ indicates that the pairwise distance of loci i and j in structure s is in distance bin $[d_q,d_{q+1})$. We enforce that by a combination of truncated harmonic potentials with a tolerance t (*Methods*, **Extended Data Figures 1** and **2**):

$$\mathcal{U}_{ijqs}^{f} = \frac{k^f}{2}\begin{cases} \left[\left\|\vec{\boldsymbol{x}}_{is} - \vec{\boldsymbol{x}}_{js}\right\|_2 - \left(d_q - t\right)\right]^2, if\ \left\|\vec{\boldsymbol{x}}_{is} - \vec{\boldsymbol{x}}_{js}\right\|_2 \leq d_q - t \\ 0, \quad if\ d_q - t \leq \left\|\vec{\boldsymbol{x}}_{is} - \vec{\boldsymbol{x}}_{js}\right\|_2 \leq d_q + t \\ \left[\left\|\vec{\boldsymbol{x}}_{is} - \vec{\boldsymbol{x}}_{js}\right\|_2 - \left(d_q + t\right)\right]^2, if\ \left\|\vec{\boldsymbol{x}}_{is} - \vec{\boldsymbol{x}}_{js}\right\|_2 \geq d_q + t \end{cases}$$

The residual error ($0 \leq \eta \leq 1$) is defined as:

$$\eta_{ijqs}^{f} = \begin{cases} \frac{\left\|\vec{\boldsymbol{x}}_{is} - \vec{\boldsymbol{x}}_{js}\right\|_2}{d_q - t} - 1, if\ \left\|\vec{\boldsymbol{x}}_{is} - \vec{\boldsymbol{x}}_{js}\right\|_2 \leq d_q - t \\ 0, \quad if\ d_q - t \leq \left\|\vec{\boldsymbol{x}}_{is} - \vec{\boldsymbol{x}}_{js}\right\|_2 \leq d_q + t \\ \frac{\left\|\vec{\boldsymbol{x}}_{is} - \vec{\boldsymbol{x}}_{js}\right\|_2}{d_q + t} - 1, if\ \left\|\vec{\boldsymbol{x}}_{is} - \vec{\boldsymbol{x}}_{js}\right\|_2 \geq d_q + t \end{cases}$$

Ensemble Hi-C

The latent variable $w_{ijs} = 1$ indicates that loci i and j form a contact in structure s. We then only apply an upper harmonic bound:

$$\mathcal{U}_{ijs}^{w} = \begin{cases} \frac{k_{HiC}}{2}\left[\left\|\vec{\boldsymbol{x}}_{is} - \vec{\boldsymbol{x}}_{js}\right\|_2 - 2(R_i^{ex} + R_j^{ex})\right]^2, if\ \left\|\vec{\boldsymbol{x}}_{is} - \vec{\boldsymbol{x}}_{js}\right\|_2 \geq 2(R_i^{ex} + R_j^{ex}) \\ 0, \quad otherwise \end{cases}, \quad R_j^{ex} = 2r_0.$$

$$\eta_{ijs}^{w} = \begin{cases} \frac{\left\|\vec{\boldsymbol{x}}_{is} - \vec{\boldsymbol{x}}_{js}\right\|_2}{C} - 1, \quad if\ \left\|\vec{\boldsymbol{x}}_{is} - \vec{\boldsymbol{x}}_{js}\right\|_2 \geq 2(R_i^{ex} + R_j^{ex}) \\ 0, \quad otherwise \end{cases}$$

SPRITE

$r_{i_1,\ldots,i_n,s} = 1$ indicates that loci $i_1, \ldots, i_n$ colocalize in structure s. We introduce a massless particle (centroid, no excluded volume effect) in structure s located in the cluster geometric center, $\vec{\boldsymbol{x}}_{Gs} = \frac{1}{n}\sum_{g\in\{1,\ldots,n\}}\vec{\boldsymbol{x}}_{i_g s}$. We introduce the following spatial restraint:

$$\mathcal{U}_{i_1,\ldots,i_{n,s}}^{r} = \begin{cases} \frac{k_{SPRITE}}{2}\sum_{g\in\{1,\ldots,n\}}\left[\left\|\vec{\boldsymbol{x}}_{i_g s} - \vec{\boldsymbol{x}}_{Gs}\right\|_2 - C^{SPRITE})\right]^2 \quad if\ \left\|\vec{\boldsymbol{x}}_{i_g s} - \vec{\boldsymbol{x}}_{Gs}\right\|_2 \geq C^{SPRITE} \\ 0 \quad otherwise \end{cases},$$

Where $C^{SPRITE} = r_n - r_0$ (*Methods*) and $r_n = r\sqrt[3]{n/\rho}$ indicates the chosen radius associated with a cluster of n loci and ρ volumetric density (*Methods*, **Extended Data Figures 1** and **2**), and loci are harmonically restrained to the centroid in order to promote geometric integrity. Each of the terms in the summation is associated with a residual error (see also "Ensemble Hi-C").

$$\eta^r_{i,\dots,i_{n,s}} = \left\{\eta^w_{i_g s}\right\}_{g\in\{1,\dots,n\}}, \qquad \eta^w_{i_g} = \begin{cases} \dfrac{\left\|\vec{\boldsymbol{x}}_{i_g s} - \vec{\boldsymbol{x}}_{Gs}\right\|_2}{r_n - r_0} - 1, & if\ \left\|\vec{\boldsymbol{x}}_{i_g s} - \vec{\boldsymbol{x}}_{Gs}\right\|_2 \geq r_n - r_0 \\ 0 & otherwise \end{cases}$$

Tracking the residual errors of all particles enables to assess whether a single cell SPRITE cluster is present in the population or not. If $\eta^r_{i_k} = 0, \forall g = 1, \dots, n$, then the cluster is there.

Additional restraints: polymer and volume

Polymer restraints Each genome chromosome is represented as a chain of 200k base-pair resolution coarse-grained loci, subjected to connectivity and steric restraints, i.e.

$$\begin{aligned} U_{polymer} &= U_{steric} + U_{chain} \\ &= \sum_{i\neq j} A^{chain}_{ij}\left[1 + \cos\left(\frac{\pi\left\|\vec{\boldsymbol{x}}_{is} - \vec{\boldsymbol{x}}_{js}\right\|_2}{R_C}\right)\right] + \sum_{i,i+1} \frac{K_b}{2}\left[\left\|\vec{\boldsymbol{x}}_{is} - \vec{\boldsymbol{x}}_{js}\right\|_2 - 2(R^{ex}_i + R^{ex}_j)\right]^2 \end{aligned}$$

$$R_c = 2r_0, \qquad A^{chain}_{ij} = \left(\frac{2r_0}{\pi}\right)^2,$$

Steric interactions are modeled using a soft potential with cutoff R_c and interaction amplitude A^{chain}_{ij} and are bivariate, i.e. act between two loci. The interconnectivity term between neighboring loci (within the same chain) is a harmonic upper interaction (i.e., only acts when the distance exceeds the contact distance l^C) of elastic constant K_b. The associated residual errors read:

$$\eta_{steric,s} = \begin{cases} 0, & if\ \left\|\vec{\boldsymbol{x}}_{is} - \vec{\boldsymbol{x}}_{js}\right\|_2 \geq R_c \\ \dfrac{\left\|\vec{\boldsymbol{x}}_{is} - \vec{\boldsymbol{x}}_{js}\right\|_2}{R_c} - 1, & otherwise \end{cases},$$

$$\eta_{chain,s} = \begin{cases} \dfrac{\left\|\vec{\boldsymbol{x}}_{is} - \vec{\boldsymbol{x}}_{js}\right\|_2}{2(R_i^{ex} + R_j^{ex})} - 1, & if\ \left\|\vec{\boldsymbol{x}}_{is} - \vec{\boldsymbol{x}}_{js}\right\|_2 \geq 2(R_i^{ex} + R_j^{ex}) \\ 0, & otherwise \end{cases}$$

Nuclear confinement A lamina DamID restraint with a negative (attractive) elastic constant efficiently models the effects of the volume confinement of the nuclear lamina (*Methods*):

$$U_{vol,s} = \begin{cases} -\dfrac{|k_{DamID}|}{2}\left(\dfrac{1}{\sqrt{\kappa_{is}(r,0)}} - 1\right)^2 \|\vec{\boldsymbol{x}}_{is}\|^2, & if\ |\kappa_{is}| \geq 1 \\ 0 & otherwise \end{cases}$$

In this specific case, the contact range c_r is set to zero: the restraint is applied any time the geometric center of a locus is outside of the lamina. The residual error is:

$$\eta_{vol,s} = \begin{cases} \left(1 - \sqrt{1/k_{is}}\right), & if\ \ \kappa_i > 1 \\ 0 & otherwise \end{cases}$$

The cumulative Hamiltonian is then defined as follows:

$$\begin{aligned} U_{tot} = & \sum_s \sum_{chr} U_{polymer} \\ & + \sum_s U_{vol,s} \\ & + \sum_s \sum_i \sum_q \mathcal{U}_{iqs}^b + \sum_s \sum_i \mathcal{U}_{is}^v + \sum_s \sum_{i<j} \sum_q \mathcal{U}_{ijqs}^f + \sum_s \sum_{i<j} \mathcal{U}_{ijs}^w + \sum_s \sum_n \sum_{C_n} \mathcal{U}_{i_1,\ldots,i_n,s}^r \\ & = \sum_s U_{tot,s} \end{aligned}$$

Such cumulative polymer-and-restraint-Hamiltonian is then relaxed. The problem can be decomposed into S independent sub-problems, since any two structures are independent of one another ($U_{tot} = \sum_s U_{tot,s}$), which makes this step heavily parallelizable from a computational standpoint. Multiple iterations of Simulated Annealing MD[3] are used to efficiently relax structures into a stable state, by minimizing constraints violations. In the first, the volume confinement and steric interaction terms are scaled down when chromosome configurations are random to facilitate optimization when the genome structure is far from the global optimum[4]. A final round of conjugate

gradient[5] is performed to adjust local structures in order the reach the optimum with zero constraints violations. Both simulated annealing MD and conjugate gradient calculations are implemented in an efficient parallel platform using the LAMMPS[6] kernel.

4. Synthetic data simulations

Simulated Hi-C, lamina DamID and FISH (both radial and pairwise) data were extracted from the ground truth population as detailed in the *Methods* section. In particular, FISH probes I were selected randomly across all chromosomes, FISH pairs (I, J) were selected by first downsampling the 15453 haploid loci using a uniform stride of 5, building all the pairs out of those, and then sampling randomly across this data set. A couple of technical remarks are in order. First, the same exact set of simulation parameters was used to generate all populations, in order to ensure consistency: eventually, all simulated populations satisfy more than 99.995% of the imposed restraints.

Second, data are incorporated into a population in a fashion similar to the steps adopted for real data, by resorting to decreasing optimization thresholds (see also **Extended Data Figures 1-2**). In particular, non-zero final threshold values are used here too, which represent the highest accuracy we are confident the models could achieve. This reproduces the standard scenario where data are only known within a confidence range, and probability values that are too small could be misconstrued as noise or a perturbation.

5. Iterative contact refinement step

Since loci that are not expected to form a contact are not explicitly being restrained from doing that, excess contacts cannot be prevented, which are shown to lead to excessively compact structures that cannot be relaxed easily. A heuristic method has been put it place to compensate for such an effect, by allowing to assess the actual portion of expected contacts that are available for allocation, which then affects the way the activation distance d_{act}^{IJ} is computed in Hi-C and DamID assignment steps. The empirical procedure relies on the predicament that when a population expresses more contacts than it should, we reduce the assignment probability: a lower probability (with fewer restraints) should be equally effective.

Assume the expected number of contacts Sp_{ij}^{input} is the sum of a number of effective contacts that are actually imposed, N_{eff}, and a number of incidental contacts (that are also expressed but not imposed), N_{inc}:

$$Sp_{ij}^{input} = N_{eff} + N_{inc} = N_{eff} + \eta(S - N_{eff})$$

The number of incidental contacts is here expressed as a fraction of the number of non-applied (non-enforced) contacts. The latter term can originate from cooperative effects, which automatically bring loci closer without an explicit bonding term operating. We can solve for probability $p_0 = N_{eff}/S$: $p_0 = \frac{p^{input}-\eta}{1-\eta}$. This is an effective probability which controls the number of restraints to be enforced. Now, we need an estimate for the scalar η.

Let us to compare the factual number of contacts in the population (expressed by the tensor $A_{ij}^{X} = \sum_{s=1}^{S} \boldsymbol{W}_{ijs}^{(k-1),\mathrm{X}}$) with the predicted number of contacts from the previous assignment step ($A_{ij}^{assig} = \sum_{s=1}^{S} \boldsymbol{W}_{ijs}^{(k-1),\mathrm{assig}}$):

$$\sum_{s=1}^{S} \boldsymbol{W}_{ijs}^{(k-1),\mathrm{X}} = \sum_{s=1}^{S} \boldsymbol{W}_{ijs}^{(k-1),\mathrm{assig}} + \left(S - \sum_{s-1}^{S} \boldsymbol{W}_{ijs}^{(k-1),\mathrm{assig}}\right)\eta_{ij}$$

We can solve for η_{ij}:

$$\eta_{ij} = \frac{\sum_{s=1}^{S} \boldsymbol{W}_{ijs}^{(k-1),\mathrm{X}} - \sum_{s=1}^{S} \boldsymbol{W}_{ijs}^{(k-1),\mathrm{X}}}{S - \sum_{s=1}^{S} \boldsymbol{W}_{ijs}^{(k-1),\mathrm{assig}}} = \frac{A_{ij}^{X} - A_{ij}^{assig}}{1 - A_{ij}^{assig}},$$

which can then be plugged into the previous equation to find a corrected assignment probability $p_0 = \frac{p^{input}-\eta}{1-\eta}$, which is then used to update the activation distance d_{ij}^{act}. Please note that the correction is only implemented if there is a contact excess.

6. HFFc6 experimental data pre-processing

Ensemble Hi-C data[7]

4DN portal identifier: 4DNES2R6PUEK

We used *in situ* Hi-C datasets from HFFc6 cell line (reference genome hg38)[7]. Similar to the protocol by ref.[8], low bin sequence coverage 3% regions were discarded during the normalization process. For data normalization, we adopted the same Knight-Ruiz normalization method used in ref.[9], leading to a normalized contact frequency matrix $\boldsymbol{G} = (g_{ij})_{K\times K}$ at 20 kb resolution.

Contact frequencies at 20kb resolution are converted to contact probabilities by scaling frequencies with a normalization factor $\mathrm{f}^{\max}$, which is chosen to represent the contact frequency value at which two bins have a 100% probability to form a contact.

$$p_{ij} = min\,(\frac{g_{ij}}{f^{max}}, 1),$$

with $\boldsymbol{P} = (p_{ij})_{K\times K}$ as the contact probability matrix at 20kb resolution. The value of $\mathrm{f}^{\max}$ is chosen so that the average number of contacts per 200 kb genomic regions is not larger than the maximum possible packing density based on the known genome occupancy, bead radius and contact distance tolerance of 2 times a bead radius. Our experience and geometric considerations show that the maximum average number of contacts is 24, which is average number of contacts a domain has at saturation level, i.e. a value at which no more contact restraints can be satisfied. These values produce optimal results.

Generating the input probability matrix $\boldsymbol{A} = \left(a_{IJ}\right) \in \mathbb{R}^{(\mathrm{H}\times\mathrm{H})}$ at 200kbp resolution is then defined as follows. If $b(I)$ is the set of all 20kb bins in 200kb genomic region I and $b(J)$ is the set of all 20kb bins in 200kb genomic region J, then $\mathrm{p}_{\alpha\beta}$ is the contact probability set of all pairwise combinations between bins in $\mathrm{b(I)}$ and $\mathrm{b(J)}$, and the contact probability matrix $\boldsymbol{A} = (a_{IJ})$ at 200kb resolution is calculated as:

$$a_{IJ} = mean(\,top10\%\,\{p_{\alpha\beta}\colon\ \alpha \in b(I), \beta \in b(J)\}\,),$$

which is the average value of the top 10% ranked 20kb level contact probabilities $\mathrm{p}_{\alpha\beta}$. In the case that some contacts are extremely higher than the surrounding contacts, these contacts were identified as outliers by $\{\mathrm{p}\colon \mathrm{p} > \mu + 1.5\mathrm{IQR}\,\}$, where $\mathrm{p} \in \{\mathrm{p}_{\alpha\beta}\colon \alpha \in \mathrm{b(I)}, \beta \in \mathrm{b(J)}\}$ and $\mu = \mathrm{mean}\{\mathrm{p}_{\alpha\beta}\colon \alpha \in \mathrm{b(I)}, \beta \in \mathrm{b(J)}\}$. The IQR refers to the interquartile range of $\{\mathrm{p}_{\alpha\beta}\}$. These outliers were excluded from the calculation of a_{IJ}.

The joint determination for the optimal $\mathrm{f}^{\max}$ and 200kb mapping a_{IJ} is performed iteratively until the resulting average number of contacts per 200-kb genomic region is maximally ~24 (within a certain tolerance).

After obtaining the contact probability matrix at 200-kb resolution, we identified bins that have spurious and isolated inter-chromosomal interaction probabilities higher than 0.2 (only 50 out of ~187 million possible inter chromosomal interactions), and removed the corresponding bins in the 20-kb raw matrix (290 out of 154,423 bins).

We then repeated the entire process, including KR normalization at 20kb resolution, and regenerated the 200-kb contact probability matrix where no inter-chromosomal contact probability is higher than 0.2 following the same procedure explained above.

Finally, probabilities for nearest neighbors are set to 1 to ensure chain integrity.

This hierarchical procedure for generating 200kb probability matrices ensures that two contact maps at different resolutions are consistent with one another, by default.

We performed extensive benchmarking by generating multiple populations with different setups and assessed the quality of each setup by predicting orthogonal experimental data. Our analysis revealed that the best setup requires an up-scaling of all the inter-chromosomal interactions by a factor of 1.5. We observed that an up-scaling of inter-chromosomal contact probabilities can be be circumvented by integrating other data modalities, which confirmed our results from the synthetic calculation section, which showed that data biases can be corrected through data integration.

Lamina DamID[10]

4DN portal identifier: 4DNESXZ4FW4T

Mapping data to 200-kb resolution

Each region (5-kb) in the Lamina DamID data was mapped to 200-kb regions with an overlap of 50% or higher. After mapping, the signals of the multiple 5-kb regions mapped to each 200-kb region were averaged (we first took the inverse log2 of the signals, then averaged and took the log2 of the average value).

Estimating lamina contact probabilities from Lamina DamID signals

For Lamina DamID data processing we adapt our established protocol as described in Li *et al.*[2] We convert the experimental lamin B1 DamID signals[10] (indicated as sig_I for genome domain I)

into an array of lamina contact probabilities e_I. Chromatin regions with a baseline signal larger than 1 ($sig_I \geq 1.0$, Dam-LaminB1/Dam ratio) would have a lamina contact probability larger than 0, therefore we set all signals lower than 1 to 0. Then, lamin contact probabilities e_I are computed as follows:

$$e_I = \kappa \frac{sig_I}{\overline{sig}}$$

Where $\overline{sig}$ is the genome-wide average of DamID signal[10] and κ is a conversion factor to be determined. To do that, we use data from single-cell lamin DamID experiments (Kind *et al.*)[11] ; if $\{e_{Kind,i} | i = 1, \dots, N_{Kind}\}$ are the contact probabilities from single cell-lamin DamID data, then we use their average contact probability $\bar{e}_{Kind}$ as a conversion factor:

$$\kappa = \bar{e}_{Kind} = \frac{1}{N_{Kind}} \sum_i e_{Kind,i} = 0.29$$

For the sake of completeness, we also used different conversion factors κ ranging from 0.15 to 0.40 to generate different sets of lamina contact probabilities $E = \{e_I | I = 1, \dots, H\}$; each data set was then used to generate a Hi-C + lamina DamID populations, and assess the accuracy of predicted lamin B1 DamID, SON TSA-seq and 3D HIPMap FISH data by comparing with available experimental data (see below for preprocessing of those data). This screening eventually confirmed that a conversion factor $\kappa = \bar{e}_{Kind} = 0.29$ performs best. So, the preprocessed lamina DamID probabilities used to generate HD, HDS and HDSF populations (see **Figure 2**, **3** and **4**) are computed from the raw data[10] as follows:

$$e_I = \frac{sig_I}{\overline{sig}} 0.29$$

3D HIPMap data[12]

4DN portal identifiers: https://data.4dnucleome.org/publications/80007b23-7748-4492-9e49-c38400acbe60/

We looked into the summary table accompanying the raw data and only retained those pairs passing the accuracy test[12]. Each FISH probe was mapped onto a 200kb resolution in our model. For each pair, all distances provided in the raw data were extracted and ordered to obtain a

discrete cumulative distribution of distances. We used a polynomial Savoy-Golay filter[13] for interpolating each cumulative distribution and drew 1000 uniformly sampled distance values from that. The resulting set of 1000 distances per distribution are the target distances that are then restrained in the simulation. Specifically, those distances we use as minimal FISH distances (see *Assignment*). In the simulations we only included those pairs separated by at least 10Mb genomic distance (resulting to 51 pairs).

DNA SPRITE data[14]

4DN portal identifier: 4DNESJYGTI8S

We first filtered out mitochondrial chromatin and outliers. We then mapped colocalized loci to 200kb resolution and selected fully inter-chromosomal clusters involving different loci only, up to 6 chromosomes (overall, 6617 clusters). We generated the appropriate family of tensors $\boldsymbol{T}_n = (t_{I_1,\dots I_n})$ (see *Methods*) and used it as input to IGM calculations.

DNA SPRITE data for HFFc6 was kindly provided by the Guttman lab at CALTECH, and are not yet publicly accessible.

SON TSA-seq[15]

4DN portal identifier: 4DNES85R9TIB

Each region in the TSA-seq data was mapped to 200-kb regions with an overlap of 50% or higher. After mapping, each 200-kb region had multiple TSA-seq regions, therefore the signals mapped to each 200-kb region were averaged (we first took the inverse log2 of the signals, then averaged and took the log2 of the average value).

Transcription data[16]

ENCODE accession number: ENCSR735JKB

RNA-seq read counts at plus and minus strands were summed up for each 200-kb region for each replicate in the experiment, then the average total reads is calculated from the replicates. Two

regions are defined: T10: Top 10% of the regions with the highest mapped RNA-seq reads, B10: Bottom 10% of the regions with the lowest mapped RNA-seq reads. Regions with zero reads are discarded before identifying T10/B10 regions.

7. Chromosomal Hi-C correlations

Chromosome	SCC	Pearson
chr1	0.90	0.97
chr2	0.90	0.98
chr3	0.91	0.98
chr4	0.89	0.99
chr5	0.89	0.99
chr6	0.90	0.98
chr7	0.91	0.98
chr8	0.89	0.98
chr9	0.86	0.96
chr10	0.89	0.98
chr11	0.91	0.98
chr12	0.90	0.98
chr13	0.92	0.96
chr14	0.91	0.96
chr15	0.86	0.95
chr16	0.85	0.96
chr17	0.84	0.97
chr18	0.91	0.98
chr19	0.84	0.97
chr20	0.87	0.97
chr21	0.91	0.96
chr22	0.85	0.94

chrX	0.91	0.98
chrY	0.60	0.91

Table S3 Stratum adjusted correlation coefficients (SCC)[17] (see *Methods*) and standard Pearson's correlation coefficients between experimental input and output chromosomal Hi-C maps from structures in the HDSF population (see **Figure 2**).

References

1. Tjong, H. *et al.* Population-based 3D genome structure analysis reveals driving forces in spatial genome organization. *Proc. Natl. Acad. Sci. U. S. A.* (2016) doi:10.1073/pnas.1512577113.
2. Li, Q. *et al.* The three-dimensional genome organization of Drosophila melanogaster through data integration. *Genome Biol.* **18**, 145 (2017).
3. Kirkpatrick, S., Gelatt, C. D. & Vecchi, M. P. Optimization by Simulated Annealing. *Science* **220**, 671–680 (1983).
4. Hua, N. *et al.* Producing genome structure populations with the dynamic and automated PGS software. *Nat. Protoc.* **13**, 915–926 (2018).
5. Hestenes, M. R. & Stiefel, E. Methods of conjugate gradients for solving linear systems. *J Res Natl Bur Stand* **49**, 409–436 (1952).
6. Plimpton, S. Fast Parallel Algorithms for Short-Range Molecular Dynamics. *J. Comput. Phys.* **117**, 1–19 (1995).
7. Krietenstein, N. *et al.* Ultrastructural Details of Mammalian Chromosome Architecture. *Mol. Cell* **78**, 554-565.e7 (2020).
8. Rao, S. S. P. *et al.* A 3D map of the human genome at kilobase resolution reveals principles of chromatin looping. *Cell* **159**, 1665–1680 (2014).
9. Imakaev, M. *et al.* Iterative correction of Hi-C data reveals hallmarks of chromosome organization. *Nat. Methods* **9**, 999–1003 (2012).
10. Wang, Y. *et al.* SPIN reveals genome-wide landscape of nuclear compartmentalization. *Genome Biol.* **22**, 36 (2021).
11. Kind, J. *et al.* Genome-wide Maps of Nuclear Lamina Interactions in Single Human Cells. *Cell* **163**, 134–147 (2015).
12. Finn, E. H. *et al.* Extensive Heterogeneity and Intrinsic Variation in Spatial Genome Organization. *Cell* **176**, 1502-1515.e10 (2019).
13. Savitzky, Abraham. & Golay, M. J. E. Smoothing and Differentiation of Data by Simplified Least Squares Procedures. *Anal. Chem.* **36**, 1627–1639 (1964).
14. Quinodoz, S. A. *et al.* Higher-Order Inter-chromosomal Hubs Shape 3D Genome Organization in the Nucleus. *Cell* **174**, 744-757.e24 (2018).
15. Zhang, L. *et al.* TSA-seq reveals a largely conserved genome organization relative to nuclear speckles with small position changes tightly correlated with gene expression changes. *Genome Res.* **31**, 251–264 (2021).

16. Dunham, I. *et al.* An integrated encyclopedia of DNA elements in the human genome. *Nature* **489**, 57–74 (2012).
17. Yang, T. *et al.* HiCRep: assessing the reproducibility of Hi-C data using a stratum-adjusted correlation coefficient. *Genome Res.* **27**, 1939–1949 (2017).
