# Supplementary material for: Integrative genome modeling platform reveals essentiality of rare contact events in 3D genome organizations
Source: Nat Methods. 2022 Jul 11;19(8):938–49. doi: 10.1038/s41592-022-01527-x (PMC9349046; doi:10.1038/s41592-022-01527-x)
Supplement: Supplementary file 2 — Reporting Summary [file 41592_2022_1527_MOESM2_ESM.pdf]

nature research

Corresponding author(s): Prof. Frank Alber

Last updated by author(s): Mar 8, 2022

Reporting Summary

Nature Research wishes to improve the reproducibility of the work that we publish. This form provides structure for consistency and transparency in reporting. For further information on Nature Research policies, see our Editorial Policies and the Editorial Policy Checklist.

Statistics

For all statistical analyses, confirm that the following items are present in the figure legend, table legend, main text, or Methods section.

n/a	Confirmed	
☐	☒	The exact sample size (*n*) for each experimental group/condition, given as a discrete number and unit of measurement
☒	☐	A statement on whether measurements were taken from distinct samples or whether the same sample was measured repeatedly
☐	☒	The statistical test(s) used AND whether they are one- or two-sided<br>*Only common tests should be described solely by name; describe more complex techniques in the Methods section.*
☒	☐	A description of all covariates tested
☒	☐	A description of any assumptions or corrections, such as tests of normality and adjustment for multiple comparisons
☐	☒	A full description of the statistical parameters including central tendency (e.g. means) or other basic estimates (e.g. regression coefficient) AND variation (e.g. standard deviation) or associated estimates of uncertainty (e.g. confidence intervals)
☐	☒	For null hypothesis testing, the test statistic (e.g. *F*, *t*, *r*) with confidence intervals, effect sizes, degrees of freedom and *P* value noted<br>*Give P values as exact values whenever suitable.*
☒	☐	For Bayesian analysis, information on the choice of priors and Markov chain Monte Carlo settings
☒	☐	For hierarchical and complex designs, identification of the appropriate level for tests and full reporting of outcomes
☐	☒	Estimates of effect sizes (e.g. Cohen's *d*, Pearson's *r*), indicating how they were calculated

Our web collection on statistics for biologists contains articles on many of the points above.

Software and code

Policy information about availability of computer code

Data collection	Populations of diploid genome structures were generated using our Integrative Genome Modeling (IGM) platform, which is deposited on GitHub (www.github.com/alberlab/igm).
Data analysis	The codes used in our work are based on standard, publicly available software packages. Data pre- and post-processing and generation of figure panels were performed using the Anacondav4.10 package (matplotlibv3.4, Scikit-learnv1.0, scipyv1.5 and networkxv2.3). Panels were then assembled using Adobe Illustrator. The software Chimera1.13 was used for visualization of the 3D structures generated.

For manuscripts utilizing custom algorithms or software that are central to the research but not yet described in published literature, software must be made available to editors and reviewers. We strongly encourage code deposition in a community repository (e.g. GitHub). See the Nature Research guidelines for submitting code & software for further information.

Data

Policy information about availability of data

All manuscripts must include a data availability statement. This statement should provide the following information, where applicable:

- Accession codes, unique identifiers, or web links for publicly available datasets
- A list of figures that have associated raw data
- A description of any restrictions on data availability

The following datasets were used in this study to generate or validate the structures: ensemble Hi-C (4DN portal, accession code 4DNES2R6PUEK), laminB1 DamID (4DN portal, accession code 4DNESXZ4FW4T), 3D HIPMap FISH (4DN portal, https://data.4dnucleome.org/publications/80007b23-7748-4492-9e49-c38400acbe60), single-cell SPRITE (4DN portal, accession code 4DNESJYGTI8S, private), SON TSA-seq (4DN portal, 4DNES85R9TIB), transcription data (ENCODE, accession number ENCSR735JKB). Super-resolution single cell imaging data are available at the referenced papers in the manuscript. The data that support the findings of this study

(including structure populations, configuration files and pre-processed data input files) are available from the corresponding author upon request. The configuration files and pre-processed data input files are sufficient to reproduce the structure populations with the IGM software.

Field-specific reporting

Please select the one below that is the best fit for your research. If you are not sure, read the appropriate sections before making your selection.

☒ Life sciences ☐ Behavioural & social sciences ☐ Ecological, evolutionary & environmental sciences

For a reference copy of the document with all sections, see nature.com/documents/nr-reporting-summary-flat.pdf

Life sciences study design

All studies must disclose on these points even when the disclosure is negative.

Sample size	Genome populations with N=1000 structures have been used. Previous studies have indicated that N=1000 is sufficient to robustly predict structural features (see also https://www.biorxiv.org/content/10.1101/2021.07.11.451976v1.full). Populations with a larger number of structures provide only incremental and not substantial increases in accuracy (if any), which takes much longer to generate.
Data exclusions	No data points were excluded from the analysis.
Replication	Three independent population replicates were generated for each simulation setup. When applicable, standard deviations of predictions across the three replicates were added to the mean prediction as error bars.
Randomization	All our population calculations start out with fully randomized genome configurations.
Blinding	Blinding was effectively performed as identical analysis scripts were applied for all structure populations with no human intervention.

Reporting for specific materials, systems and methods

We require information from authors about some types of materials, experimental systems and methods used in many studies. Here, indicate whether each material, system or method listed is relevant to your study. If you are not sure if a list item applies to your research, read the appropriate section before selecting a response.

Materials & experimental systems

n/a	Involved in the study
☒	☐ Antibodies
☒	☐ Eukaryotic cell lines
☒	☐ Palaeontology and archaeology
☒	☐ Animals and other organisms
☒	☐ Human research participants
☒	☐ Clinical data
☒	☐ Dual use research of concern

Methods

n/a	Involved in the study
☒	☐ ChIP-seq
☒	☐ Flow cytometry
☒	☐ MRI-based neuroimaging
